# GWAS analysis reveals distinct pathogenicity profiles of Australian *Parastagonospora nodorum* isolates and identification of marker-trait-associations to septoria nodorum blotch

**DOI:** 10.1038/s41598-021-87829-0

**Published:** 2021-05-12

**Authors:** Huyen T. T. Phan, Eiko Furuki, Lukas Hunziker, Kasia Rybak, Kar-Chun Tan

**Affiliations:** Centre for Crop and Disease Management, School of Molecular and Life Sciences, Curtin University, Perth, WA Australia

**Keywords:** Plant breeding, Plant genetics, Plant stress responses

## Abstract

The fungus *Parastagonospora nodorum* is the causal agent of septoria nodorum leaf blotch (SNB) and glume blotch which are common in many wheat growing regions in the world. The disease is complex and could be explained by multiple interactions between necrotrophic effectors secreted by the pathogen and matching susceptibility genes in wheat. An Australian *P. nodorum* population was clustered into five groups with contrasting properties. This study was set to identify their pathogenicity profiles using a diverse wheat panel of 134 accessions which are insensitive to SnToxA and SnTox1 in both in vitro and in vivo conditions. SNB seedling resistance/susceptibility to five representative isolates from the five clusters, responses to crude culture-filtrates (CFs) of three isolates and sensitivity to SnTox3 semi-purified effector together with 11,455 SNP markers have been used for linkage disequilibrium (LD) and association analyses. While quantitative trait loci (QTL) on 1D, 2A, 2B, 4B, 5B, 6A, 6B, 7A, 7D chromosomes were consistently detected across isolates and conditions, distinct patterns and isolate specific QTL were also observed among these isolates. In this study, SnTox3–*Snn3-B1* interaction for the first time in Australia and SnTox3–*Snn3-D1* interaction for the first time in bread wheat were found active using wild-type isolates. These findings could be due to new SnTox3 haplotype/isoform and exotic CIMMYT/ICARDA and Vavilov germplasm used, respectively. This study could provide useful information for dissecting novel and different SNB disease components, helping to prioritise research targets and contributing valuable information on genetic loci/markers for marker-assisted selection in SNB resistance wheat breeding programme.

## Introduction

*Parastagonospora* (syn. *Stagonospora*; *Phaeosphaeria*, *Septoria*) *nodorum* (Berk.) Quaedvlieg, Verkley & Crous is a haploid necrotrophic fungal pathogen which causes septoria nodorum blotch (SNB) of wheat (*Triticum aestivum*)^1–3^. Leaf blotch caused by the pathogen results in significant damage to leaves and glumes of wheat leading to reduced photosynthesis, therefore lower growth and yield and adversely affect grain quality. Yield losses due to SNB are typically reported to be 5–15% in Western Australia^4^, but up to ~ 30% was also observed^5^. The disease occurs due to multiple interactions between proteinaceous necrotrophic effectors (NEs) secreted by *P. nodorum* and corresponding dominant susceptibility genes in the host (*Snn*)^6,7^. Effects from these interactions are normally additive^8–10^, however, epistasis between interactions has been frequently observed^10–14^.

To date, eight such effectors and nine matching host dominant susceptibility genes have been identified and characterised to different extents. This includes SnToxA–*Tsn1*^9,15–19^, SnTox1–*Snn1*^20–23^, SnTox2–*Snn2*^9,19,24^, SnTox3–*Snn3-B1*^11,25^, SnTox3–*Snn3-D1*^26^, SnTox4–*Snn4*^27^, SnTox5–*Snn5*^10^, SnTox6–*Snn6*^13^, and SnTox7–*Snn7*^28^. Evidence from these studies have shown that the interactions play important roles in inducing SNB at all plant development stages.

Of these eight effectors, only three have been cloned and fully characterised for their contribution to SNB. SnToxA is a small protein which induces necrosis typical of NE and its sensitivity was elicited by the presence of a dominant susceptibility gene, *Tsn1*, residing on wheat chromosome 5BL^18^. Twenty-two haplotypes of SnToxA encoding 14 unique protein isoforms have been identified globally^29,30^. Another small but extremely cysteine-rich NE protein was designated as SnTox1 with a chitin binding-like motif at the carboxyl terminus. Disulphide bonds between cysteine pairs are considered to be crucial for the stability of many fungal effector proteins once secreted, and the eight cysteine pairs of SnTox1 were shown to be highly conserved among isoforms^23^. The host receptor of SnTox1 was mapped as a major quantitative trait locus (QTL) to chromosome 1BS^21,23^. Twenty-two nucleotide haplotypes of SnTox1 have been reported which encode 17 different isoforms^31^. Sensitivity to SnTox3, another small cysteine-rich protein, requires the expression of either the host sensitivity gene *Snn3-B1* or *Snn3-D1* located on wheat chromosome 5BS and 5DS, respectively^26^. The receptor *Snn3-D1* locus which was found in the diploid wild wheat progenitor *Aegilops tauschii* was assumed to be a homoeologue of *Snn3-B1*^26^. To date, this *Snn3-D1* receptor has never been identified on the D sub-genome of hexaploid wheat with the exception of some synthetic hexaploid wheats and SnTox3–*Snn3-B1* interaction has never been detected in Australian conditions. The study by Ghaderi et al*.*^31^ reported 13 nucleotide haplotypes of SnTox3. In addition, mounting evidence from biochemical and genetic analyses indicates that *P. nodorum* possesses several undiscovered NEs that may also function as inverse gene-for-gene determinants in SNB^12^.

SNB disease management currently relies on chemical control. Although this is an important measure in the integrated disease control program, the deployment of genetic resistance offers a more economically and environmentally sustainable disease-controlling means in wheat production. However, in most cases SNB resistance/susceptibility was shown to be inherited quantitatively and controlled by small additive effects and significant genotype by environment interactions^8^. In addition, there are very limited SNB resistance sources available in commercial cultivars^32^. The approach of growing cultivars with increased durable SNB resistance requires information on resistance/susceptibility alleles available from existing and novel sources. QTL mapping has been widely used for this purpose. For SNB seedling resistance/susceptibility, numerous QTL identified on 19 of the 21 wheat chromosomes by genetic mapping have been reported^33^. A similar number of SNB resistance QTL were also detected at adult stage for both leaf blotch and glume blotch^33^. The QTL mapping studies not only help to identify novel SNB associated QTL/interactions but have also provided evidence for the roles that NE-*Snn* interactions play in SNB resistance in the field^14,19,34^.

QTL mapping also provided valuable information on cloning of *Tsn1*^35^, *Snn1*^36^ and a marker for *Snn3-B1*^37^. However, QTL mapping suffers from limited power in detecting small QTL effects^38^, limited variation which exists between the two parental lines, as well as large QTL intervals which depend on the population sizes and recombination frequencies^39^. Genome-wide association studies (GWAS) can overcome the constraints by effectively identifying many natural allelic variations in a large set of unrelated individuals^40^.

For disease resistance to be durable and sustainable, it is important to identify resistance sources which withstand a wide range of pathotypes or genetically diverse isolates of the pathogen of interest. The Australian *P. nodorum* population has been characterised and consists of five genetically distinct sub-groups with distinct properties^41^. The five groups classified into core and transient sub-populations. While the former has high genetic diversity and was found abundantly and over a long time period, the latter was low in genetic variation, detected at one or very few locations and exists for a limited time frame. To facilitate the SNB resistance breeding processes and avoid laborious and time-consuming phenotyping, molecular markers linked to resistance or susceptibility genes/QTL or strong marker-trait-associations (MTAs) detected from GWAS analysis should be used for marker-assisted selection and gene pyramiding. Susceptibility QTL/genes should be ‘bred out’ whereas ones which confer SNB resistance will be ‘bred in’ elite lines. The objectives of the current study are to evaluate the pathogenicity profiles of recent Australian *P. nodorum* representatives from the five identified groups and to identify resistance/susceptibility sources from a wheat panel selected from CIMMYT/ICARDA and Vavilov collections^42^ which are insensitive to ToxA and SnTox1. In addition, information on markers tightly linked to the identified resistance will be obtained for further evaluation of their uses in marker-assisted selection programs.

## Results

### Phenotypic evaluation

#### Disease responses

**Figure 1:**
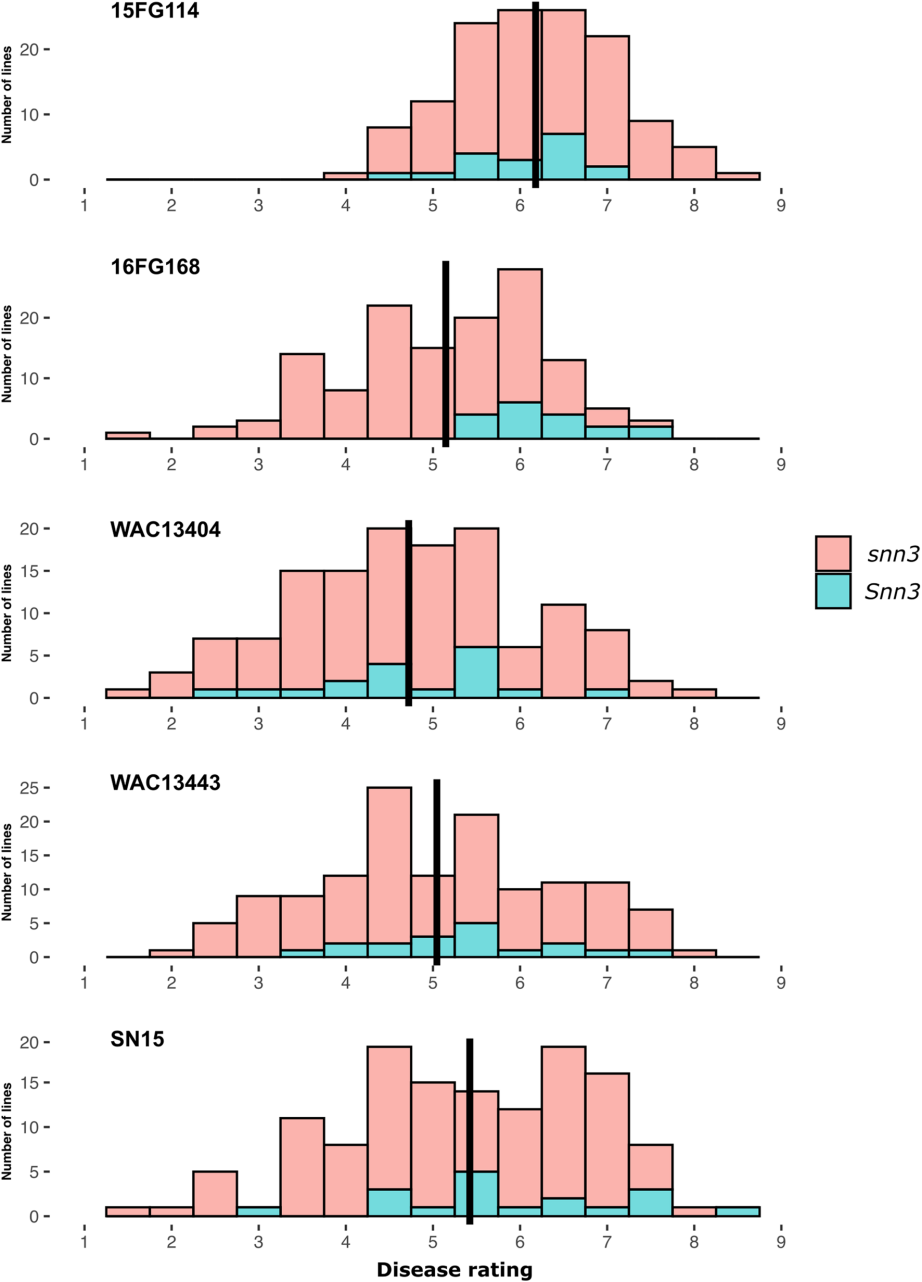
Histograms showing Septoria nodorum blotch ratings (1–9) of 134 wheat lines against five different isolates of *Parastagonospora nodorum* as noted on the top left of each graph. The vertical black bar indicates the mean of the disease rating from the respective isolate. Cyan colour indicates the proportion of lines carrying *Snn3*. This Figure was produced using ggplot2 version 3.3.3 (2020-12-30) in R^43^.

Pathogenicity profiles of five isolates each from the five Australian *P. nodorum* sub-populations (Table 1) were studied by inoculating them on a panel of 134 wheat lines (Supplementary Table S1). A diverse response to SNB was observed among the tested lines (Fig. 1; P < 2e − 16). Average disease responses varied greatly from 4.00 to 8.33 (average 6.18) with 15FG114, while they ranged from 1.67 to 7.83 (average 4.72) with WAC13404 (Supplementary Fig. S1). Significant differences were found in disease severity caused by these isolates (P < 2e^−16^). A least significant difference (LSD) t test on the disease reactions to the isolates showed that they form three groups: the most pathogenic was 15FG114, the least one was WAC13404; SN15, 16FG168 and WAC13443 make the intermediate group where 16FG168 is slightly more pathogenic than the others (Fig. 1, Supplementary Fig. S1).

**Table 1:** Australian *Parastagonospora nodorum* isolates (Phan et al. 2020) used in this study.

Isolate	Year	Source	ToxA		Tox1		Tox3		DAPC group assignment
			Haplotype	Isoform	Haplotype	Isoform	Haplotype	Isoform	
WAC13443	2011	DPIRD	H10	IH10	H02	IH02	H01	IH01	1
15FG114	2014	This study	H10	IH10	H12	IH02	H01	IH01	2
SN15	2001	This study	H10	IH10	H01	IH01	H01	IH01	3
WAC13404	2011	DPIRD	H01	IH01	H01	IH01	H05	IH01	4
16FG168	2016	This study	H10	IH10	H01	IH01	H02	IH02	5

#### Culture filtrate

SNB occurrence and severity is determined by multiple interactions between effectors secreted by the pathogen and receptors from the host. Depending on various factors, a number of effectors can be produced in in vitro conditions^44,45^. Three of the five isolates (SN15, 15FG114 and 16FG168) were chosen for effector profiling of their crude CFs in in vitro conditions. The isolates were selected due to their strong responses obtained from initial CF screening of all five isolates (data not shown). For the evaluation of correlations between an observed SNB phenotype and damage induced by any effector present in crude culture filtrate from three selected isolates, single leaves of seedlings were infiltrated and scored. Here, significantly higher chlorosis and necrosis scores were found with isolate 16FG168 compared to that of SN15 and 15FG114 (Supplementary Fig. S1).

### Strong population structure in the wheat panel

**Figure 2:**
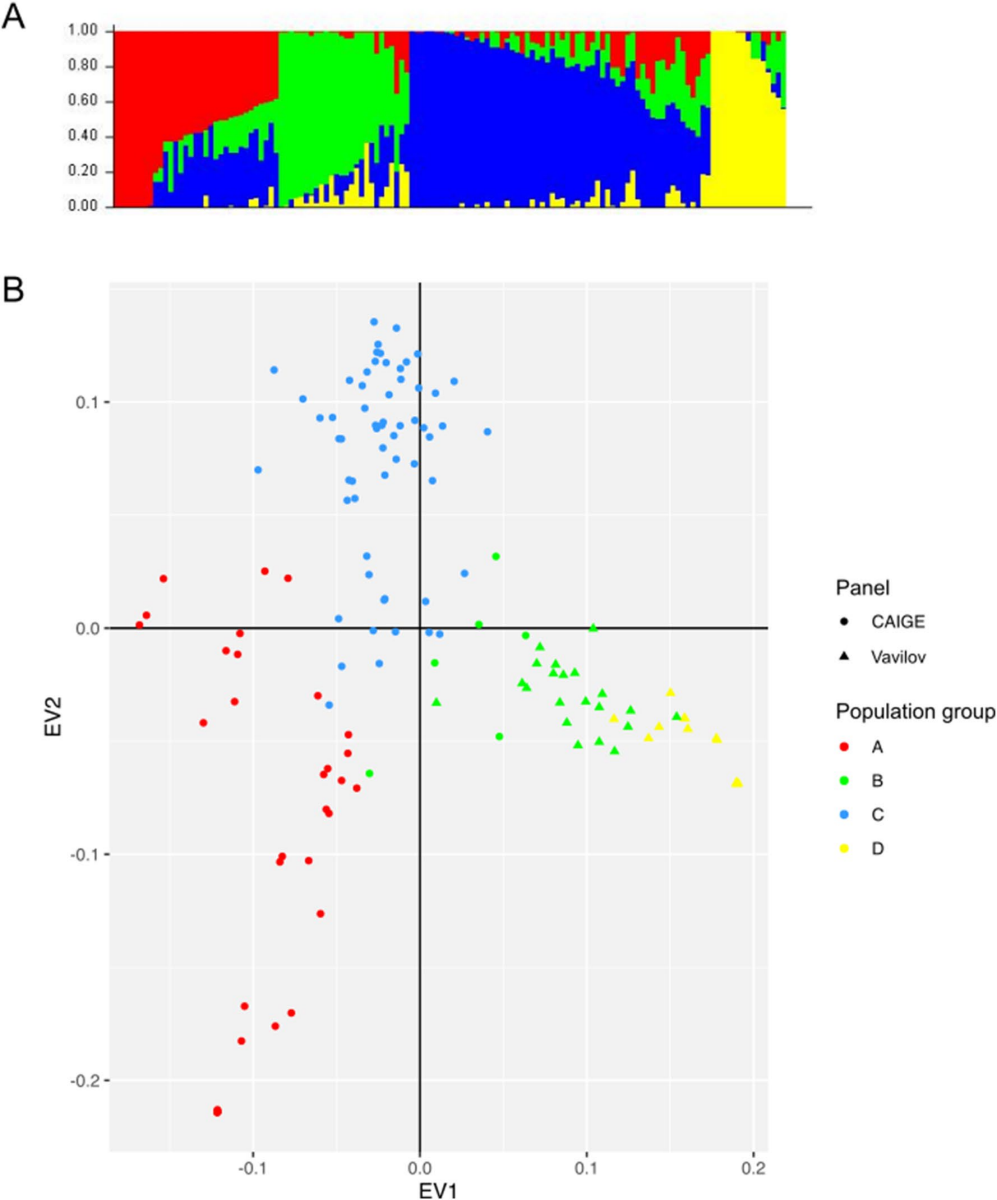
Principal component analysis of 134 wheat lines based on a ~ 6500 SNP. (**A**) STRUCTURE analysis with the optimal *K* value of 4. (**B**) Lines in the PCA plot are colour-coded according to the grouping. (**A**) was produced by a built-in function in STRUCTURE, (**B**) was created by using ggplot2 version 3.3.3 (2020-12-30) in R^43^.

Ten initial runs in STRUCTURE each with K from 1 to 10 identified the best fit number of K is 4 for our wheat panel (Fig. 2A). This is consistent with PCA analysis (Fig. 2B) where three separate and an admixed group were apparent. 9.29% of variance existing in the wheat collection was explained by PC1, while PC2 and PC3 accounted for 7.44% and 5.68% of the total variance (Fig. 2).

**Figure 3:**
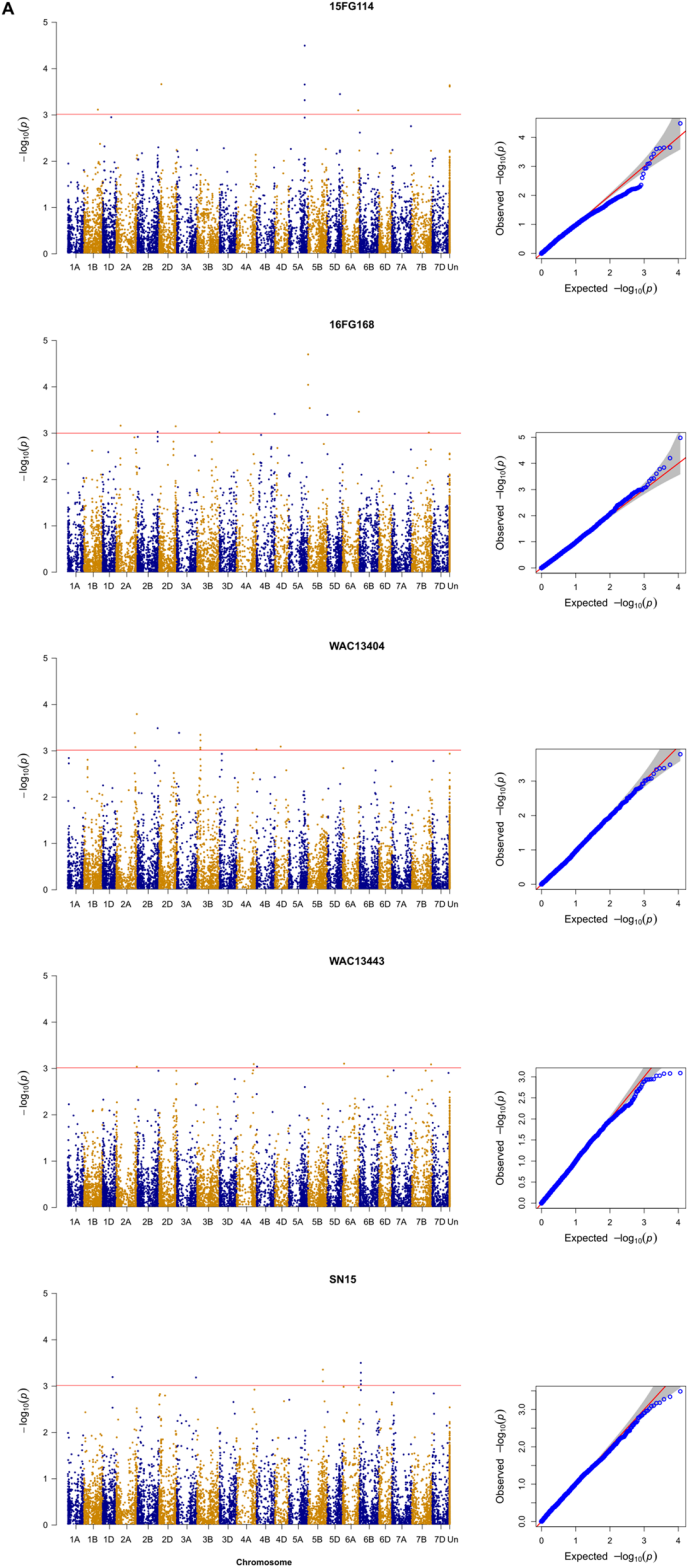

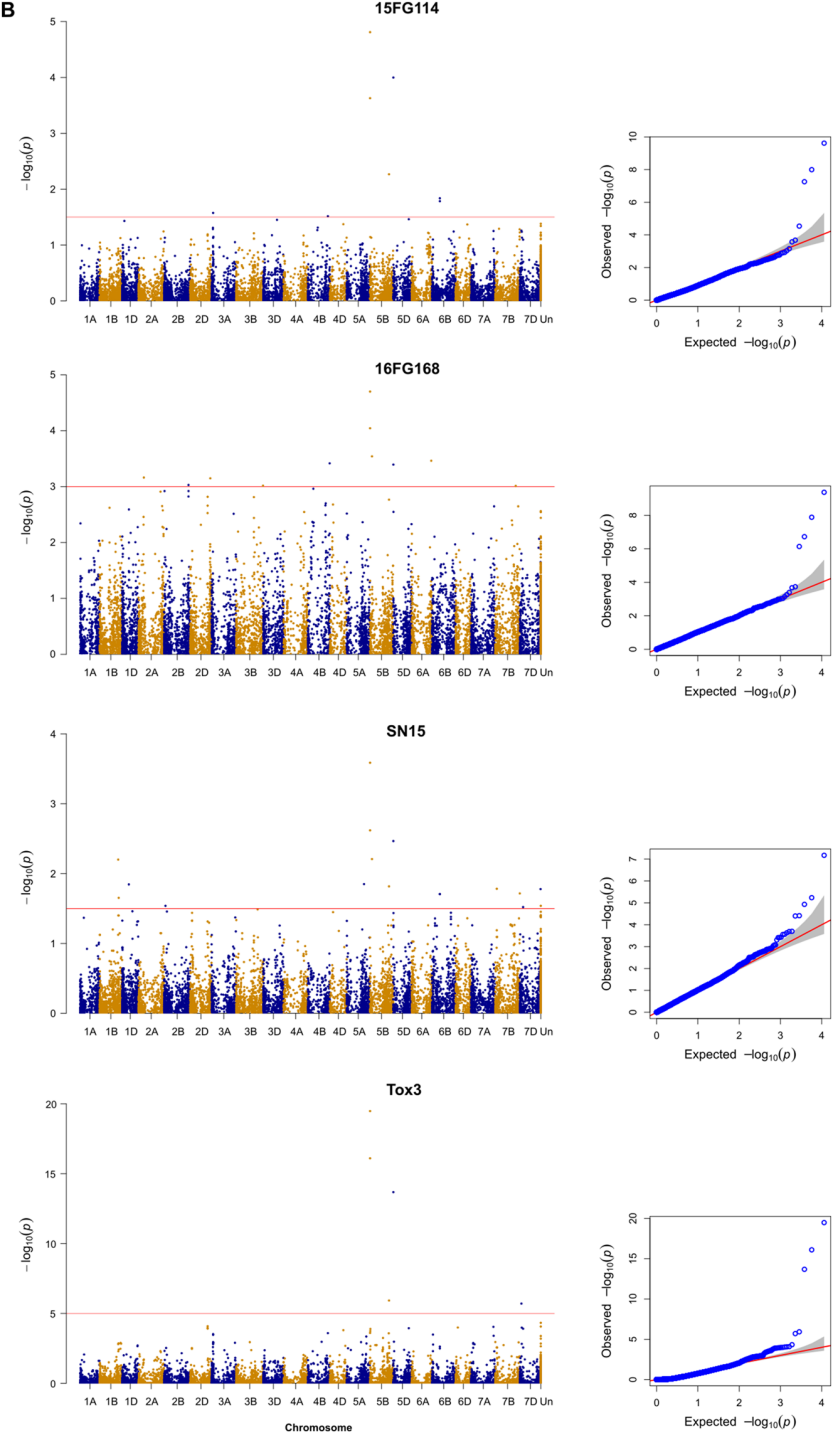
(Manhattan QQ): Genome-wide association scan. Manhattan plots based on Compressed Mixed Linear Model (CMLM) represent − log10(*P* value) for SNPs distributed across all chromosomes (x axis); Un, unknown. The red horizontal line indicates a significance threshold for SNP markers, corresponding to *P* < 0.001, with the exception of Tox3 infiltration (P < 1e−5). (**A**) Whole plant spray datasets from isolates as indicated. (**B**) (facing page): Culture filtrate dataset from isolates as indicated and Tox3 protein only. Quantile–Quantile (QQ) plots to right of each model show the expected null distribution of *P* values vs. observed *P* values. The Manhattan plots were produced by R ‘qqman’ package (Stephen Turner, https://cran.r-project.org/web/packages/qqman/qqman.pdf). The QQ plots were produced by GAPIT package in R^47^.

#### GWAS analysis: marker trait associations (MTAs)

GWAS analysis was performed to find associations between 11,455 high quality SNP markers and five traits of SNB reactions, three CF responses and SnTox3 sensitivity (Table 2, Fig. 3). The best result was obtained using GAPIT with PCA and kinship. Overall, the analysis revealed 30 putative genomic regions associated with resistance to SNB from nine different treatments including five disease, three CF and one effector responses (Table 2). The Q–Q plots in Fig. 3 showed the compressed mixed linear model (CMLMs)^46^ used was a suitable analytical model for the association analysis. The number of MTAs ranged from 4 for SNB of isolate 15FG114, SN15 and WAC13443 to 11 for SN15 CF (Table 2).

**Table 2:** Significant associations between single nucleotide polymorphism (SNP) markers (marker trait association—MTA) and different *Parastagonospora nodorum* culture filtrate (CF) infiltrations or infections (whole plant spray, WPS).

Treatment	SNP marker	Allele	Chr^a^	Position (Mbp)	Genomic region ID	*P*-value	Effect	R^2^
Tox3	*AX.95632742*	A/G	2A	675.90	2A1	4.72E−05	1.13	0.80
	*AX.94610974*	C/T	5BS*	6.65	5B1	3.31E−20	1.95	0.98
	*AX.94457982*	A/C	5BL	584.07	5B4	1.19E−06	− 1.32	0.82
	*AX.95126753*	C/T	5DS*	4.34	5D	2.09E−14	− 1.39	0.90
	*AX.94978939*	C/T	7A	652.61	7A	1.98E−06	1.20	0.82
CF-16FG168	*AX.94774467*	G/T	2B	213.25	2B2	4.37E−04	0.62	0.44
	*AX.95165387*	A/C	2B	747.82	2B3	7.50E−04	0.57	0.44
	*AX.94753171*	A/G	2D	612.46	2D	6.13E−04	1.14	0.44
	*AX.94828722*	A/G	4B	665.35	4B	3.83E−04	− 1.05	0.45
	*AX.94610974*	C/T	5BS*	6.65	5B1	1.99E−05	0.86	0.47
	*AX.95126753*	C/T	5DS*	4.34	5D	4.03E−04	− 0.65	0.44
	*AX.95019005*	A/G	6A	595.18	6A2	2.92E−04	− 0.43	0.45
	*AX.94681771*	A/T	7A	657.09	7A	9.69E−04	0.56	0.40
CF-15FG114	*AX.94429024*	A/T	3A	47.82	3A1	7.07E−04	0.83	0.49
	*AX.94531841*	A/C	4B	611.51	4B	7.76E−04	− 1.82	0.49
	*AX.94610974*	C/T	5BS*	6.65	5B1	2.40E−10	1.45	0.64
	*AX.94457982*	A/C	5BL	584.07	5B4	2.93E−05	− 1.33	0.52
	*AX.95126753*	C/T	5DS*	4.34	5D	1.01E−08	− 1.23	0.58
	*AX.94407285*	C/T	6B	239.87	6B2	2.12E−04	− 1.81	0.51
CF-Sn15	*AX.94950274*	C/T	1B	566.84	1B	3.97E−05	− 0.95	0.40
	*AX.95253275*	G/T	1D	203.32	1D2	2.04E−04	− 0.81	0.39
	*AX.95171319*	G/T	2A	761.76	2A2	8.37E−04	1.05	0.37
	*AX.94610974*	C/T	5BS*	6.65	5B1	6.70E−08	0.97	0.48
	*AX.95010548*	C/G	5B	64.63	5B2	3.84E−05	1.20	0.40
	*AX.94457982*	A/C	5BL	584.07	5B4	2.32E−04	− 0.89	0.39
	*AX.95126753*	C/T	5DS*	4.34	5D	4.85E−06	− 0.72	0.42
	*AX.94459459*	C/T	6A	555.58	6A2	8.38E−04	2.45	0.37
	*AX.94407285*	C/T	6B	239.87	6B2	3.86E−04	− 1.36	0.38
	*AX.94535772*	A/G	7B	47.00	7B	2.72E−04	− 1.24	0.38
	*AX.94860625*	A/G	7D	632.34	7D	2.78E−04	0.58	0.38
WPS_WAC13404	*AX.94935613*	C/T	2A	758.39	2A2	1.66E−04	1.25	0.23
	*AX.95255981*	C/T	2B	744.69	2B3	3.35E−04	0.99	0.21
	*AX.94526408*	A/G	3B	117.59	3B	3.68E−04	− 0.66	0.22
	*AX.94948233*	C/T	3D	480.83	3D	7.06E−04	− 1.08	0.21
	*AX.94428968*	C/T	4A	736.15	4A	9.62E−04	1.02	0.16
WPS_16FG168	*AX.94694976*	C/T	1D	7.61	1D1	9.07E−04	− 1.28	0.19
	*AX.95632742*	A/G	2A	675.90	2A1	8.56E−04	1.13	0.49
	*AX.94935613*	C/T	2A	758.39	2A2	3.76E−04	0.87	0.26
	*AX.95133018*	C/G	2D	636.07	2D	6.28E−05	0.49	0.28
	*AX.94792356*	C/T	4D	50.64	4D	2.49E−04	0.76	0.21
	*AX.94610974*	C/T	5BS*	6.65	5B1	1.05E−05	0.79	0.31
	*AX.95629214*	A/G	6A	544.48	6A2	4.65E−04	0.50	0.20
	*AX.94436382*	C/T	7D	632.01	7D	1.44E−04	− 0.89	0.21
WPS_15FG114	*AX.94427355*	A/C	1D	379.59	1D3	7.93E−04	− 0.83	0.17
	*AX.95007069*	A/G	2B	134.04	2B1	2.24E−04	0.42	0.19
	*AX.94699167*	C/T	5A	586.60	5A	3.30E−05	− 0.83	0.21
	*AX.94575839*	C/T	6A	570.00	6A2	8.09E–−04	− 0.58	0.17
				0.00				
WPS_Sn15	*AX.94723571*	C/T	1D	362.01	1D3	6.59E−04	− 1.40	0.14
	*AX.94954416*	A/G	3A	705.31	3A2	6.74E−04	− 1.27	0.14
	*AX.94804165*	A/G	5B	548.33	5B3	4.55E−04	− 1.41	0.14
	*AX.95097189*	C/T	6B	47.14	6B1	3.26E−04	− 1.58	0.15
WPS_WAC13443	*AX.94935613*	C/T	2A	758.39	2A2	7.70E−04	1.06	0.17
	*AX.94772629*	A/G	4A	636.97	4A	8.32E−04	0.88	0.17
	*AX.95086097*	C/T	6A	50.00	6A1	7.18E−04	1.16	0.17
	*AX.94750259*	C/G	7D	613.62	7D	7.88E-04	0.94	0.17

^a^Chromosome; asterisk indicates that this marker corresponds to Snn3-5B (5BS) or Snn3-5D (5DS).

There are common QTL or MTAs among these treatments such as *Snn3-B1* (5B1) on chromosome 5BS (*AX.94610974*) which were detected in five different conditions and *Snn3-D1* (5D, *AX.95126753*) in four (Table 2). These MTAs are receptors of SnTox3 as reported before^11,26^. Apart from the SnTox3–*Snn3-B1/D1* interactions, other common MTAs across different isolates and experimental conditions including 1D3, 2A1, 2A2, 2B3, 2D, 4A, 4B, 5B4, 6A2, 6B2, 7A and 7D were also identified (Table 2, Fig. 3). While 14 of the 30 MTAs were detected multiple times, 16 were specific to particular isolates or conditions (Table 2).

For isolate 16FG168, three common QTL (2D, 5B1 and 6A2; Table 2, Fig. 3) were observed in both in vitro and in vivo conditions suggesting that effectors interacting with these receptor loci could be secreted in vitro. However, distinct MTA profiles were detected between the conditions for isolates 15FG114 and SN15. While 10 MTA/QTL regions (1B, 1D2, 3A1, 4B, 5B2, 5B4, 5D, 6B2, 7A, 7B) were detected exclusively in vitro conditions, 11 (1D1, 1D3, 2B2, 3A2, 3B, 3D, 4A, 4D, 5A, 5B3, 6A1) were only found in planta (Table 2, Fig. 3). There were various MTAs which were detected in CF of one isolate and SNB in another/others confirming their existence (Table 2, Fig. 3).

### In silico gene annotation of the QTL regions

Of the 20 genomic regions which were significantly correlated with either crude CF or SNB disease severity, a total of 405 plant defence related genes were found (Supplementary Table S2). They ranging from 2 at 4A (6370 Mb) to 156 defence-related genes within interval 4A (736 Mb) with an average of 13.4 genes per interval (Supplementary Table S2). These predicted defence-related genes include major families like LRR (Leucine-rich repeat), NB-ARC (NB-ARC domain), cytochrome P450, and Pkinase (Protein kinase). In addition, other proteins such as sugar transporter protein, peroxidase, ABC transporter, mitochondrial carrier protein and acidic chitinase were also observed (Supplementary Table S2).

### Effect of SnTox3–*Snn3-B1/D1* interactions

Histograms of the disease responses and analysis of variance by the isolates (Fig. 1, Supplementary Fig. 1) revealed the significant correlation between increased disease reactions and the presence of SnTox3 by isolate 16FG168 (P = 6.05e^−06^). The other four isolates did not show any correlation between disease reactions and the presence of SnTox3 (Fig. 1, Supplementary Fig. 1).

Two known MTAs/QTL responding to SnTox3 located on 5BS (5B1, *Snn3-B1*, 6.654 Mb) and 5DS (5D, *Snn3-D1*, 4.343 Mb) were detected from sensitivity to SnTox3 semi-purified infiltrations and CFs from three *P. nodorum* chosen isolates for CF analysis. These three isolates and other two (WAC13443 and WAC13404) used in this study all carry *SnTox3*. While SN15, 15FG114 and WAC13443 harbour *SnTox3* haplotype 1, WAC13404 and 16FG168 possess haplotype 5 and 2, respectively (Table 1, Phan et al*.*^41^). The SnTox3 haplotype 1 and 5 encode the same SnTox3 protein isoform I, haplotype 2 encodes SnTox3 isoform II with a 3 aa difference (Supplementary Fig. S3). However, in planta the MTA/QTL or SnTox3–*Snn3-B1* interaction was only found significantly associated with SNB caused by isolate 16FG168 (Table 2).

### Tox3 expressions in planta of SN15 and 16FG168

To test whether the expression of Tox3 is different between isolate SN15 carrying Tox3 isoform I and 16FG168 possessing Tox3 isoform II, which could explain the ability to detect the SnTox3–*Snn3-B1* interaction in SNB using isolate 16FG168, the two isolates were inoculated in two SnTox3 sensitive lines (Halberd and Wyalkatchem) and infected leaf tissue was collected at 48 h post inoculation. SnTox3 expression was not significantly different between cultivars nor isolates but wheat line x isolate interaction was highly significant. Tox3 expression was highest with Sn15 and Wyalkatchem and significantly different to the other three treatments (*P* = 0.0002, Supplementary Fig. S2).

## Discussion

SNB is a complex and thus difficult to manage fungal disease which is economically important in many wheat growing parts of the world^5,33^. In Australia, $108 million has been lost to the disease annually^48^. To be able to control the disease effectively, information on pathogenicity profiles of the pathogen which exist within a local population is invaluable. Our previous study on Australian *P. nodorum* population genetic diversity has revealed a five-group structure with contrasting properties^41^. Infection assays indicate that newly emerged isolates were more pathogenic, demonstrating evidence of selection. The aim of this study was to gain knowledge on pathogenicity potential and their disease profile’s components of the five groups on exotic wheat lines derived from CIMMYT and the Vavilov collection. This information will help to create management strategies and to breed for durable SNB resistance.

SNB inoculation assays carried out with five representative isolates from the five sub-populations found in Australian *P. nodorum* population on a wheat panel showed that they are different in pathogenicity potential. The panel has been chosen so that ToxA–*Tsn1* and SnTox1–*Snn1* interactions were removed to reduce disease complexity and increase chances of detecting novel interactions. Marker-trait association analysis revealed distinct QTL/MTA profiles that consist of different genomic regions associated with their disease severity (Table 2). Isolates from core group 1 (15FG114, Table 1) followed by transient groups 5 and 3 (16FG168 and SN15, Supplementary Fig. 1) were the most pathogenic. QTL profiles of these three isolates were almost distinct except 1D and 6A (Table 2) which were shared between 15FG114 and SN15 and 16FG168 and 15FG114, respectively. This suggests that these isolates may deploy different mechanisms for their infection strategies.

Two sub-groups (core group 1 and transient group 5) are contemporary and among the most pathogenic, which is consistent with the finding in Phan et al*.*^41^. Group 3 isolate was as aggressive as group 5 suggesting that this panel consists of wheat lines of different genetic backgrounds compared to the commercial cultivars used in Phan et al*.*^41^ study where group 5 was the most virulent. More pathogenic isolates found in more temporal groups suggests that these virulent isolates adapted to more resistant local wheat lines and this further strengthens our hypothesis that local cultivars drive local *P. nodorum* evolution.

*Snn3-D1* (genomic region 5D) was believed to be a homoeologue of *Snn3-B1* and is another receptor of SnTox3^26^. To date, this SnTox3–*Snn3-D1* interaction was only detected in *Aegilops tauschii*, a diploid progenitor of modern wheat. This is the first time the SnTox3–*Snn3-D1* was observed in a bread wheat panel. The *Snn3-D1* linked marker *AX.95126753* (CS-5D: 4342851) located in close proximity to marker *Xcdf18* (CS-5D: 5597846) which is only 0.75 cM away from the *Snn3-D1* gene^26^. Among 17 lines carrying this *Snn3-5D1* allele, fifteen and two were originated from the CAIGE project which originally stem from a CYMMIT and Vavilov collections, respectively. Three of the CAIGE lines are synthetic wheat based on information available to us (Supplementary Table S3). Synthetic hexaploid wheat is created by artificially combining genetic resources from tetraploid and diploid relatives of wheat into current elite cultivars. This suggests that the *Snn3-D1* allele may have been introduced to bread wheat long ago.

While both SnTox3–*Snn3-B1* and SnTox3–*Snn3-D1* interactions were active in all three crude CFs tested (Table 2), which confirmed that SnTox3 is produced in in vitro conditions, only the SnTox3–*Snn3-B1* interaction was detected and thus contributed to SNB severity in the infection assay by isolate 16FG168 (5B1, Table 2). The SnTox3–*Snn3-B1/D1* undetectable interaction in SNB assessments by four other isolates was not surprising and an explanation for the phenomenon was proposed in Phan et al. 2016. This fact was possibly due to an epistasis effect where other interactions in this pathosystem such as SnToxA–*Tsn1*^12^, SnTox2–*Snn2*^11^, SnTox5–*Snn5*^10^, SnTox6–*Snn6*^13^ and SnTox1–*Snn1*^14^ can mask the expression of SnTox3–*Snn3-B1* during infection. The identifiable and biggest disease contributing component of the SnTox3–*Snn3-B1* interaction induced by isolate 16FG168 could be owed to differences in SnTox3 expression between the isolates, however we found no such differences (Supplementary Fig. S2). Another possible explanation is subtle differences in the SnTox3 protein, as 16FG168 carries SnTox3 isoform II (Table 1). It has been indicated at least for biotrophic and hemibiotrophic pathogens that point mutations can affect effector protein structure in such a way that the interaction with the corresponding host protein is abolished^49,50^. Although the SnTox3–*Snn3-B1* interaction was never found in SNB trials in Australia, it was quite commonly observed in SNB studies by USA or European isolates^51–53^. Keeping all this in mind, studying functional variation of different SnTox3 isoforms in the future will shed light on the phenomenon.

The detectable SnTox3–*Snn3-B1* interaction and other specific QTL to 16FG168 is a possible explanation for the more virulence of isolate 16FG168 in this study and isolates of group 5 in Phan et al*.*^41^. Continual maintenance of *Snn3* in WA wheat varieties could have led to *P. nodorum* being selected for carrying an active SnTox3 version since *Tsn1* and *Snn1* are progressively being removed through tsn1snn1 Mace and Scepter cultivars which accounted for ~ 70% of wheat growing areas in WA (https://www.agric.wa.gov.au/sites/gateway/files/2019%20Wheat%20Variety%20Guide-web.pdf).

In few cases, different MTA/QTL profiles were observed between CF and disease GWAS analysis which were originated from one isolate (Table 2). This could indicate that different suites of effectors were secreted by the isolates in different environments. Numerous studies on effector gene regulation in other fungal species have shown distinct waves of concerted expression at certain stages of plant infection and in different environments/conditions^44,45^. One example of that is ToxA. While SnToxA is not expressed in vitro, PtrToxA is expressed abundantly in that same condition and both SnToxA and PtrToxA are highly active in planta^54^. On the other hand, this study also found a few disease or CF associated genomic regions including *Snn3-B1*, 2A, 2B, 6A and 7D which are common across different tested isolates and environments. Significant effector–receptor interactions found in planta would be most suitable and relevant for pathogenicity study and SNB resistance breeding, whereas those detected from CFs (in vitro condition) would greatly facilitate the identification and cloning of pathogen effectors and receptors in the host. Our data help to establish important interactions and/or suitable research approaches for future research targets. Interactions found in both conditions would be given a priority.

It would also be interesting to decipher and understand the roles of isolate specific interactions. Twelve such specific interactions were identified in disease inoculation assays alone while six were detected with CFs of three tested isolates (Table 2). The obtained information would elucidate the role of the isolate’s adaptation to particular predominantly grown wheat cultivars over time.

Apart from the *Snn3-B1* and *Snn3-D1* loci which have been confirmed to be receptors of SnTox3 before, other QTL/MTA loci significantly associated with SNB in this study have also been reported. Namely, *Snn7* which interacts with SnTox7 resides in 2DL^28^. Two markers flanking *Snn7*, *Xcfd44* and *Xgwm349*^28^, span a physical interval from 608.63 to 629.65 Mb of 2D in Chinese Spring. Another 2D QTL was also reported recently by Lin et al.^52^ as QSnb.niab-2D.2 spanning a genomic interval from 635.95 to 637.64 Mb of the 2D reference chromosome. This study discovered a 2D QTL linked to CF and SNB disease caused by isolate 16FG168 covering a region of 612.45 to 636.06 Mb (Table 2). With this overlap, the *Snn7* locus on 2DL has a more stringent confirmation. Similarly, two genomic regions both found associated with CF and disease severity on chromosome 2A at positions 675.90 Mb and 758.39 Mb (Table 2) were described before by Lin et al.^52^ and Phan et al.^14^. These 2A QTL were found in both SNB and tan spot pathosystems^33,55^. Likewise, the 5AL QTL at physical position 586.60 Mb in this paper was co-located with a common QTL of the two diseases found in many previous studies^56–59^. These areas may encode different biological mechanisms that contribute to the disease outcomes which need to be further investigated. If they were found to confer broad range resistance or susceptibility, they would be the primary targets for incorporating into or excluding from wheat breeding materials as they play a role in both SNB and tan spot diseases of wheat.

With two cloned receptors (*Tsn1*^35^ and *Snn1*^60^), it was revealed that sequences and structure of the two genes prominently resembled those of classic plant disease resistance genes. *Tsn1* possesses three major domains of typical resistance (R) genes, protein kinase, nucleotide-binding-site, and leucine-rich repeat (NBS-LRR^35^). *Snn1* harbours a wall-associated kinase (WAK), a class of receptors which are known to activate downstream pathways typical of biotrophic pathogen resistance, a calcium binding epidermal growth factor, transmembrane, and serine/threonine protein kinase (S/TPK) domains^60^. These similar genomic regions consistently come up from various studies on different genomic backgrounds and in different environmental conditions should be of future research focus.

Searching for defense-related genes which could be our QTL candidate genes in the annotated QTL regions, we found numerous NB-ARC and NBS-LRR type genes in every significantly associated genomic region (Supplementary Table S2). The cytochrome P450 superfamily which includes key players in plant development and defence^61^ was also found abundantly in these regions. Other important components of PTI and ETI such as production of reactive oxygen species^62^, plant chitinase production^63^, serine/threonine-protein kinase^64^, ABC transporter^65^, F-box-like domain superfamily^66^ were also present in the annotated QTL regions associated with SNB responses (Supplementary Table S2). If these defence related genes in the CS genomic regions are found in homologous regions of the wheat lines used in the current study, they could help to explain the associations we observed here.

The current study has again shown the complexity of the *P. nodorum*-wheat pathosystem. It provided insights into distinct pathogenicity profiles of five *P. nodorum* sub-groups found in the Australian population structure. This information helps to understand their adaptation to different wheat cultivars and will assist in formulating breeding strategies as well as research objectives in the future.

## Materials and methods

### Plant and fungal materials

#### Wheat panel

A wheat panel which includes 99 and 35 wheat lines selected from CIMMYT Australian ICARDA Germplasm Evaluation (CAIGE, https://www.caigeproject.org.au/) and Vavilov^42^ collections, respectively, was used for the study (Supplementary Table S1). This selection was based on how sensitive they are to the three known *P. nodorum* effectors SnToxA, SnTox3 and SnTox1. Information on effector sensitivity of the Vavilov lines was obtained from our previous work^67^. The effector profiles of the CAIGE lines were obtained by subjecting 99 CAIGE lines used in this study (Supplementary Table S1) to effector infiltration screening as described in Phan et al.^68^. All wheat lines were grown in 12 cm pots containing perlite and vermiculite (The Perlite and Vermiculite Factory, Australia) at 21 °C under a 12 h light and dark cycle for 2 weeks prior to manipulation. While 18 of the 134 wheat lines in the selected panel are only sensitive to SnTox3, the majority (116) of the lines are insensitive to all the three effectors. A small proportion of Tox3 sensitive lines were included as they are important lines in our breeding program and because to date the Tox3–*Snn3-B1* interaction has never been identified in Australian conditions.

#### Wild-type *P. nodorum* isolates

Five isolates (SN15, WAC13443, 15FG114, WAC13404, 16FG168; Table 1) used for this study were from Phan et al.^41^. They are representative isolates from five sub-groups found in the *P. nodorum* Australian population structure as described that study (Table 1). The isolates were maintained on V8-PDA agar at 21 °C under a 12 h light and dark cycle for 2 weeks prior to manipulation^14^. Of these, three (SN15, 15FG114 and 16FG168) were chosen for effector characterisation of their crude CFs in intro conditions.

### Whole plant infection assay

The whole-plant spray assay technique as described in Phan et al*.*^14^ was used for seedling infections. Pycnidiospore inoculum was prepared to a concentration of 1 × 10^6^ spores/ml in 0.5% (w/v) gelatine. Tested wheat lines were planted in a completely randomised design in three replicates. Three to five seedlings of each line were grown in a 12 cm pot and considered as a repeat. Two‐week‐old wheat seedlings were sprayed with the inoculum preparation using a hand-held sprayer until runoff. Plants were placed in 100% relative humidity for 72 h, then 4 days at 90% humidity at 21 °C under a 12 h photoperiod prior to scoring. A score of 1–9 was used where 1 indicates that no disease symptoms were observed and a score of 9 signifies a fully necrotised plant as described in Phan et al*.*^68^.

### Effector and crude culture filtrate expression and infiltration

SnTox3 protein (SNOG_08981; GenBank acc; XP_001799284) was produced in *Pichia pastoris* using the pGAPzA expression system (Thermo Fisher Scientific, MA, USA)^23,25^. The *P. pastoris* culture filtrate (CF) containing the Tox3 expressed protein was harvested and desalted with 10 mm sodium phosphate buffer pH 7.0 as previously described^25^. Crude CFs containing necrosis‐inducing factors produced by the five wild-type *P. nodorum* isolates (Table 1) were generated in Fries 3 broth as previously described^21,69^. The CFs were filter sterilised prior to plant infiltration.

A simple leaf infiltration technique as described in Oliver et al*.*^70^ was used for SnTox3 and the crude CFs. A needleless 1 ml plastic syringe was used to infiltrate the expressed proteins into the first leaf of 2-week-old wheat seedlings. Infiltrated plants were kept in a Conviron growth chamber for 4 days for SnTox3-induced necrosis and 7 days for crude CFs from wild-type isolates under a 12 h photoperiod prior to scoring. Sensitivity was visually evaluated using a scale of 0–4, where a score of 0 indicates no observable reactions; 1, mild chlorosis; 2, chlorosis; 3, chlorosis with mild necrosis; 4, necrosis^71^. All infiltrations were carried out in biological triplicates.

### Curation of the genotyping data

The wheat panel of 134 lines was genotyped with the Axiom Wheat Genotyping Breeders’ Array platform which contains 35 k SNP markers^72^ and carried out by University of Bristol in collaboration with Thermo Fisher Scientific (Waltham, MA USA). Allele calling was carried out using the Affymetrix proprietary software package GTC, following the Axiom Best Practices Genotyping Workflow (http://tools.thermofisher.com/content/sfs/manuals/axiom_genotyping_solution_analysis_guide.pdf). All monomorphic markers among the 134 wheat lines were removed first, then SNPs with minor allele frequency (MAF) < 0.05 and a missing value of > 10% were filtered. The low proportion of missing marker data in the cleaned dataset was imputed using the missForest v1.4 package in R for missing data points^73^. Co-segregating markers were removed to produce a final data set of 11,455 high quality markers for GWAS analysis. For population structure adjustment and kinship analysis only, additional filtering steps discarded markers with more than 0.8 correlation coefficient with any other markers to create a smaller data set of 6523 SNP markers. The genetic positions of selected SNPs were obtained from the online database CerealsDB (http://www.cerealsdb.uk.net/cerealgenomics/CerealsDB/indexNEW.php).

### Population structure analysis

A smaller data set was generated for principal component analysis (PCA) and population structure within the wheat panel using SNPRelate package in R^74,75^ and STRUCTURE version 2.3.4^76,77^. The data set comprises of 6523 SNP markers which are totally in linkage equilibrium using LD-based SNP pruning function in SNPRelate package (ld.threshold = 0.2). Bayesian model-based clustering method in-built in STRUCTURE version 2.3.4 was used for the population structure analysis. The following settings for STRUCTURE runs were used for the initial stage to identify number of sub-populations: admixture model with correlated allele frequencies from K = 1–10 with 10 independent runs each, burn-in/MCMC of 10,000, reps 10,000. Five replicated runs were carried out each with number of populations assumed was between one and ten. The final K number, which was the best fit number of clusters in the studying panel, was determined using the Evanno method^78^ as implemented in STRUCTURE HARVESTER^79^. Grouping of each individual was done by running a simulation in STRUCTURE with best fit number of sub-populations at burn-in of 500,000, reps 750,000 for MCMC.

### Genome‐wide association studies

To adjust for population structure in GWAS analysis of the panel of 134 chosen wheat lines, information on kinship among the wheat lines was used. The kinship matrix was obtained from the R package SNPRelate^74^. A phenotype data set of eight qualitative and one quantitative trait (SnTox3 sensitivity) tested in the GWAS panel and their genotype data set of 11,455 SNPs were used to identify MTA using the compressed mixed linear model (CMLMs) analysis^46^ accounting for the population structure and kinship as fixed effects using the package GAPIT in R^47^. Marker-trait significant associations were declared using two thresholds depending on different phenotyping methods. The Bonferroni corrected P = 0.05 [− log10(P) = 5.49] was used as significant threshold for sensitivity to SnTox3 semi-purified effector where a single inverse gene-for-gene interaction was expected. For whole-plant disease infection or responses to crude culture filtrate where multiple interactions contributed to observed phenotypes, an arbitrary threshold of − log10(P) > 3 was applied, similar to the approach adopted by Halder et al*.*^80^. Each of these genomic areas was given an ID (Genomic region ID, Table2) since there could be more than one detected in the same chromosome. Manhattan plots were constructed to display the whole genome MTAs using R ‘qqman’ package (Stephen Turner, https://cran.r-project.org/web/packages/qqman/qqman.pdf).

### Candidate gene annotation in QTL regions

Candidate genes associated with resistance to SNB were identified following the method in Halder et al*.*^80^. Candidate regions flanking the most significant markers at each genomic ‘hot spot’ were defined which span a 5 megabase pairs (Mb) region (2.5 Mb up- and downstream from the chosen SNP markers). Any genes involved in plant defence mechanisms within the candidate regions were identified using the CS high confidence gene annotation RefSeq version 1.1 at T3/Wheat database (Ensembl Plant release 45, IWGSC RefSeq v1.0, Sept 2019; https://triticeaetoolbox.org/wheat/genes/).

### Tox3 expression in planta

SN15 and 16FG168 isolates which carry Tox3 isoform I and II, respectively, were chosen for a Tox3 in planta expression experiment using the wheat lines Wyalkatchem and Halberd. The wheat lines are both sensitive to Tox3. Six treatments were compared, that is, SN15/16FG168/gelatine control each on Wyalkatchem and Halberd. Total RNA from each sample was extracted using PureLink RNA Mini kit (Invitrogen by Thermo Fisher Scientific Australia, 5791 Van Allen Way, Carlsbad, CA 92008 USA). cDNAs were created using iScript cDNA Synthesis Kit (BIO-RAD Australia, Bio-Rad Laboratories 2000 Alfred Nobel Drive, Hercules, CA 94547). There were three replicates for each treatment and three technical qPCR replicates for each cDNA sample. The infection process was the same as above and samples were collected at 48 h post inoculation (hpi). Fold changes in SnTox3 expression relative to expression of actin as an endogenous (house-keeping) gene and Tox3 expression in gelatine control samples were calculated for each replicate (Supplementary Fig. S2). They were used to for Student’s t-test for significant differences in the SnTox3 expression of the samples (Supplementary Fig. S2).

### Statistical analysis

Analysis of variance (ANOVA) in SNB disease scores between isolates was done in R using basic variance analysis functions built in R version 4.0.2 (2020-06-22) (https://www.r-project.org/foundation/) and the “agricolae” package (https://cran.r-project.org/web/packages/agricolae/index.html).

All plant-related methods were performed in accordance with the guidelines and regulations applicable to Australia.

## Supplementary Information

Supplementary Figure S1–S3.

Supplementary Table S1.

Supplementary Table S2.

Supplementary Table S3.

## Data Availability

The datasets generated for this study can be found as Supplementary data.

## References

[1] Quaedvlieg W (2013). Sizing up septoria. Stud. Mycol..

[2] Solomon PS, Lowe RG, Tan K-C, Waters OD, Oliver RP (2006). *Stagonospora nodorum*: Cause of *Stagonospora nodorum* blotch of wheat. Mol. Plant Pathol..

[3] Eyal Z (1987). The Septoria Diseases of Wheat: Concepts and Methods of Disease Management.

[4] Brennan, J. P. & Murray, G. M. (NSW Agriculture, 1998) ‘Economic importance of wheat diseases in Australia.’ (NSW Agriculture: Wagga Wagga).

[5] Bhathal JS, Loughman R, Speijers J (2003). Yield reduction in wheat in relation to leaf disease from yellow (tan) spot and septoria nodorum blotch. Eur. J. Plant Pathol..

[6] Friesen TL, Faris JD (2010). Characterization of the wheat-*Stagonospora nodorum* disease system: What is the molecular basis of this quantitative necrotrophic disease interaction?. Can. J. Plant Pathol..

[7] Oliver RP, Friesen TL, Faris JD, Solomon PS (2012). *Stagonospora nodorum*: From pathology to genomics and host resistance. Annu. Rev. Phytopathol..

[8] Xu SS, Friesen TL, Mujeeb-Kazi A (2004). Seedling resistance to tan spot and *Stagonospora nodorum* blotch in synthetic hexaploid wheats. Crop Sci..

[9] Zhang Z, Friesen TL, Simons KJ, Xu SS, Faris JD (2009). Development, identification, and validation of markers for marker-assisted selection against the *Stagonospora nodorum* toxin sensitivity genes *Tsn1* and *Snn2* in wheat. Mol. Breed..

[10] Friesen TL, Chu C, Xu SS, Faris JD (2012). SnTox5–Snn5: A novel *Stagonospora nodorum* effector-wheat gene interaction and its relationship with the *SnToxA–Tsn1* and *SnTox3–Snn3-B1* interactions. Mol. Plant Pathol..

[11] Friesen TL, Zhang Z, Solomon PS, Oliver RP, Faris JD (2008). Characterization of the interaction of a novel *Stagonospora nodorum* host-selective toxin with a wheat susceptibility gene. Plant Physiol..

[12] Friesen TL, Faris JD, Solomon PS, Oliver RP (2008). Host-specific toxins: Effectors of necrotrophic pathogenicity. Cell. Microbiol..

[13] Gao Y (2015). Identification and characterization of the *SnTox6–Snn6* interaction in the *Parastagonospora nodorum*-wheat pathosystem. Mol. Plant Microbe Interact..

[14] Phan HTT (2016). Differential effector gene expression underpins epistasis in a plant fungal disease. Plant J..

[15] Liu Z (2006). The Tsn1–ToxA interaction in the wheat–*Stagonospora nodorum* pathosystem parallels that of the wheat–tan spot system. Genome.

[16] Faris JD, Friesen TL (2009). Reevaluation of a tetraploid wheat population indicates that the Tsn1–ToxA interaction is the only factor governing *Stagonospora nodorum* blotch susceptibility. Phytopathology.

[17] Faris JD (2010). Pathogen hijacking of disease resistance mechanisms in wheat. Phytopathology.

[18] Friesen TL (2006). Emergence of a new disease as a result of interspecific virulence gene transfer. Nat. Genet..

[19] Friesen TL (2009). Genetics of the *Stagonospora nodorum*-wheat interaction—evidence for a complex, toxin-based inverse gene-for-gene system. Can. J. Plant Pathol. Revue Canadienne De Phytopathologie.

[20] Liu ZH (2004). Genetic and physical mapping of a gene conditioning sensitivity in wheat to a partially purified host-selective toxin produced by *Stagonospora nodorum*. Phytopathology.

[21] Liu ZH (2004). Quantitative trait loci analysis and mapping of seedling resistance to *Stagonospora nodorum* leaf blotch in wheat. Phytopathology.

[22] Reddy L, Friesen TL, Meinhardt SW, Chao S, Faris JD (2008). Genomic analysis of the *Snn1* locus on wheat chromosome arm 1BS and the identification of candidate genes. Plant Genome.

[23] Liu Z (2012). The cysteine rich necrotrophic effector SnTox1 produced by *Stagonospora nodorum* triggers susceptibility of wheat lines harboring *Snn1*. PLoS Pathog..

[24] Friesen TL, Meinhardt SW, Faris JD (2007). The *Stagonospora nodorum*-wheat pathosystem involves multiple proteinaceous host-selective toxins and corresponding host sensitivity genes that interact in an inverse gene-for-gene manner. Plant J..

[25] Liu Z (2009). SnTox3 acts in effector triggered susceptibility to induce disease on wheat carrying the *Snn3* gene. PLoS Pathog..

[26] Zhang Z (2011). Two putatively homoeologous wheat genes mediate recognition of SnTox3 to confer effector-triggered susceptibility to *Stagonospora nodorum*. Plant J..

[27] Abeysekara NS, Friesen TL, Keller B, Faris JD (2009). Identification and characterization of a novel host-toxin interaction in the wheat-*Stagonospora nodorum* pathosystem. Theor. Appl. Genet..

[28] Shi G (2015). The wheat Snn7 gene confers susceptibility on recognition of the *Parastagonospora nodorum* necrotrophic effector SnTox7. Plant Genome..

[29] McDonald MC, Oliver RP, Friesen TL, Brunner PC, McDonald BA (2013). Global diversity and distribution of three necrotrophic effectors in *Phaeosphaeria nodorum* and related species. New Phytol..

[30] Hafez M (2020). *Parastagonospora nodorum* and related species in Western Canada: Genetic variability and effector genes. Phytopathology.

[31] Ghaderi F, Sharifnabi B, Javan-Nikkhah M, Brunner PC, McDonald BA (2020). *SnToxA*, *SnTox1*, and *SnTox3* originated in *Parastagonospora nodorum* in the Fertile Crescent. Plant. Pathol..

[32] Adhikari TB, Jackson EW, Gurung S, Hansen JM, Bonman JM (2011). Association mapping of quantitative resistance to *Phaeosphaeria nodorum* in spring wheat landraces from the USDA National Small Grains Collection. Phytopathology.

[33] Downie R (2020). Genetically Dissect Disease Interactions between *Parastagonospora nodorum* and Wheat (*Triticum aestivum* L.).

[34] Ruud AK, Windju S, Belova T, Friesen TL, Lillemo M (2017). Mapping of SnTox3–Snn3 as a major determinant of field susceptibility to *Septoria nodorum* leaf blotch in the SHA3/CBRD × Naxos population. Theor. Appl. Genet..

[35] Faris JD (2010). A unique wheat disease resistance-like gene governs effector-triggered susceptibility to necrotrophic pathogens. Proc. Natl. Acad. Sci. U.S.A..

[36] Shi G (2016). Marker development, saturation mapping, and high-resolution mapping of the *Septoria nodorum* blotch susceptibility gene *Snn3-B1* in wheat. Mol. Genet. Genom..

[37] Downie RC (2018). Assessing European wheat sensitivities to *Parastagonospora nodorum* necrotrophic effectors and fine-mapping the *Snn3-B1* locus conferring sensitivity to the effector SnTox3. Front. Plant Sci..

[38] Korte A, Farlow A (2013). The advantages and limitations of trait analysis with GWAS: A review. Plant Methods.

[39] Vales M (2005). Effect of population size on the estimation of QTL: A test using resistance to barley stripe rust. Theor. Appl. Genet..

[40] Huang X, Han B (2014). Natural variations and genome-wide association studies in crop plants. Annu. Rev. Plant Biol..

[41] Phan HTT (2020). Low amplitude boom-and-bust cycles define the *Septoria nodorum* blotch interaction. Front. Plant Sci..

[42] Riaz A (2017). Into the vault of the Vavilov wheats: Old diversity for new alleles. Genet. Resour. Crop Evol..

[43] Wickham H (2016). ggplot2: Elegant Graphics for Data Analysis.

[44] Tan K-C, Oliver RP (2017). Regulation of proteinaceous effector expression in phytopathogenic fungi. PLoS Pathog..

[45] Soyer JL (2014). Epigenetic control of effector gene expression in the plant pathogenic fungus *Leptosphaeria maculans*. PLoS Genet..

[46] Zhang Z (2010). Mixed linear model approach adapted for genome-wide association studies. Nat. Genet..

[47] Lipka AE (2012). GAPIT: Genome association and prediction integrated tool. Bioinformatics.

[48] Murray GM, Brennan JP (2009). Estimating disease losses to the Australian wheat industry. Australas. Plant Pathol..

[49] Mesarich CH (2016). A conserved proline residue in Dothideomycete Avr4 effector proteins is required to trigger a Cf-4-dependent hypersensitive response. Mol. Plant Pathol..

[50] Di X (2017). Structure–function analysis of the *Fusarium oxysporum* Avr2 effector allows uncoupling of its immune-suppressing activity from recognition. New Phytol..

[51] Richards JK (2019). Local adaptation drives the diversification of effectors in the fungal wheat pathogen *Parastagonospora nodorum* in the United States. PLoS Genet..

[52] Lin M (2020). Genetic mapping using a wheat multi-founder population reveals a locus on chromosome 2A controlling resistance to both leaf and glume blotch caused by the necrotrophic fungal pathogen *Parastagonospora nodorum*. Theor. Appl. Genet..

[53] Haugrud ARP, Zhang Z, Richards JK, Friesen TL, Faris JD (2019). Genetics of variable disease expression conferred by inverse gene-for-gene interactions in the wheat-*Parastagonospora nodorum* pathosystem. Plant Physiol..

[54] Jones DAB (2019). A specific fungal transcription factor controls effector gene expression and orchestrates the establishment of the necrotrophic pathogen lifestyle on wheat. Sci. Rep..

[55] Shankar M (2017). Loci on chromosomes 1A and 2A affect resistance to tan (yellow) spot in wheat populations not segregating for *tsn1*. Theor. Appl. Genet..

[56] Chu C-G, Friesen T, Xu S, Faris J, Kolmer J (2009). Identification of novel QTLs for seedling and adult plant leaf rust resistance in a wheat doubled haploid population. Theor. Appl. Genet..

[57] Friesen TL (2009). Host-selective toxins produced by *Stagonospora nodorum* confer disease susceptibility in adult wheat plants under field conditions. Theor. Appl. Genet..

[58] Kariyawasam GK (2016). Genetic relationships between race-nonspecific and race-specific interactions in the wheat–*Pyrenophora tritici-repentis* pathosystem. Theor. Appl. Genet..

[59] Hu W (2019). A wheat chromosome 5AL region confers seedling resistance to both tan spot and *Septoria nodorum* blotch in two mapping populations. Crop J..

[60] Shi G (2016). The hijacking of a receptor kinase-driven pathway by a wheat fungal pathogen leads to disease. Sci. Adv..

[61] Jun X, Wang X-Y, Guo W-Z (2015). The cytochrome P450 superfamily: Key players in plant development and defense. J. Integr. Agric..

[62] O’Brien JA, Daudi A, Butt VS, Bolwell GP (2012). Reactive oxygen species and their role in plant defence and cell wall metabolism. Planta.

[63] Punja ZK, Zhang Y-Y (1993). Plant chitinases and their roles in resistance to fungal diseases. J. Nematol..

[64] Zhou J, Loh Y-T, Bressan RA, Martin GB (1995). The tomato gene *Pti1* encodes a serine/threonine kinase that is phosphorylated by Pto and is involved in the hypersensitive response. Cell.

[65] Borghi L, Kang J, de Brito Francisco R (2019). Filling the gap: Functional clustering of ABC proteins for the investigation of hormonal transport in planta. Front. Plant Sci..

[66] van den Burg HA (2008). The F-box protein ACRE189/ACIF1 regulates cell death and defense responses activated during pathogen recognition in tobacco and tomato. Plant Cell.

[67] Dinglasan EG (2018). Vavilov wheat accessions provide useful sources of resistance to tan spot (syn. yellow spot) of wheat. Plant Pathol..

[68] Phan HTT (2018). Novel sources of resistance to *Septoria nodorum* blotch in the Vavilov wheat collection identified by genome-wide association studies. Theor. Appl. Genet..

[69] Tan K-C (2015). Functional redundancy of necrotrophic effectors—consequences for exploitation for breeding. Front. Plant Sci..

[70] Oliver RP, Rybak K, Solomon PS, Ferguson-Hunt M (2009). Prevalence of ToxA-sensitive alleles of the wheat gene *Tsn1* in Australian and Chinese wheat cultivars. Crop Pasture Sci..

[71] Tan K-C (2012). Quantitative variation in effector activity of ToxA isoforms from *Stagonospora nodorum* and *Pyrenophora tritici-repentis*. Mol. Plant Microbe Interact..

[72] Allen AM (2017). Characterization of a Wheat Breeders’ Array suitable for high-throughput SNP genotyping of global accessions of hexaploid bread wheat (*Triticum aestivum*). Plant Biotechnol. J..

[73] Stekhoven DJ, Bühlmann P (2012). MissForest—Non-parametric missing value imputation for mixed-type data. Bioinformatics.

[74] Zheng X (2012). A high-performance computing toolset for relatedness and principal component analysis of SNP data. Bioinformatics.

[75] R Core Team (2020). R: A Language and Environment for Statistical Computing.

[76] Pritchard JK, Stephens M, Donnelly P (2000). Inference of population structure using multilocus genotype data. Genetics.

[77] Pritchard, J. K., Wen, W. & Falush, D. Documentation for STRUCTURE software: Version 2. (2003).

[78] Evanno G, Regnaut S, Goudet J (2005). Detecting the number of clusters of individuals using the software STRUCTURE: A simulation study. Mol. Ecol..

[79] Earl DA (2012). STRUCTURE HARVESTER: A website and program for visualizing STRUCTURE output and implementing the Evanno method. Conserv. Genet. Resour..

[80] Halder J (2019). Mining and genomic characterization of resistance to tan spot, *Stagonospora nodorum* blotch (SNB), and Fusarium head blight in Watkins core collection of wheat landraces. BMC Plant Biol..

