# Supplementary material for: GWAS analysis reveals distinct pathogenicity profiles of Australian Parastagonospora nodorum isolates and identification of marker-trait-associations to septoria nodorum blotch
Source: Sci Rep. 2021 May 12;11:10085. doi: 10.1038/s41598-021-87829-0 (PMC8115087; doi:10.1038/s41598-021-87829-0)
Supplement: Supplementary file 1 — Supplementary Figure S1–S3. [file 41598_2021_87829_MOESM1_ESM.pdf]

Supplementary figures to "GWAS analysis reveals distinct pathogenicity profiles of Australian *Parastagonospora nodorum* isolates and identification of marker-trait-associations to septoria nodorum blotch"

Phan *et al*. 2021

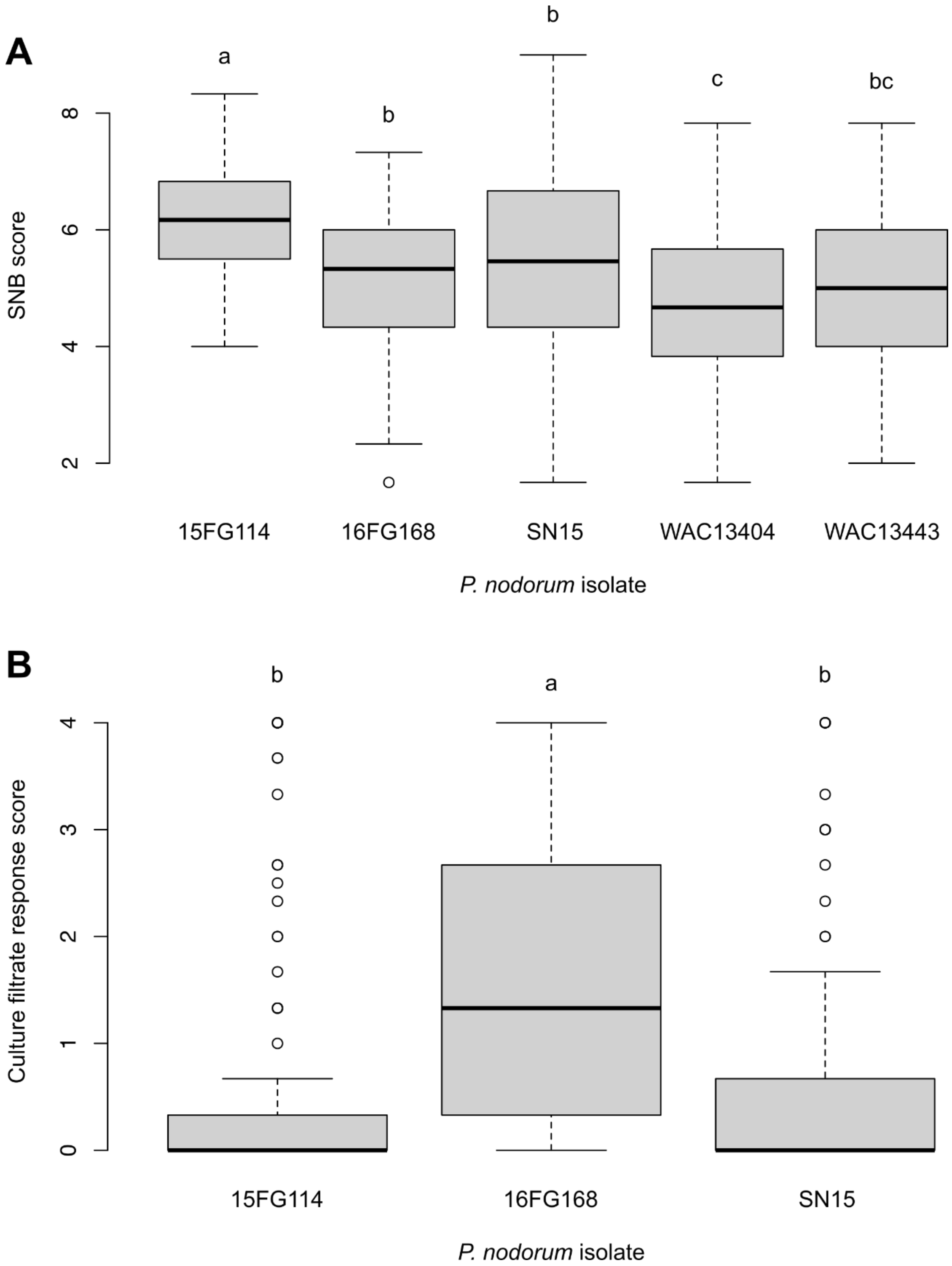

Supplementary Figure S1: Box plots illustrating variation in disease severity among five different isolates of *Parastagonospora nodorum* or their culture filtrates. A) Septoria nodorum blotch ratings. B) Culture filtrate responses. The lower and upper hinges correspond to the first and third quartiles and the upper (lower) whiskers extend from the hinge to the largest (smallest) value that is within 1.5x the interquartile range of the hinge. Data beyond the ends of the whiskers are outliers. Treatments with the same letters are not significantly different (Least Significant Difference t test). This Figure was produced using ggplot2 version 3.3.3 (2020-12-30) in R[80].

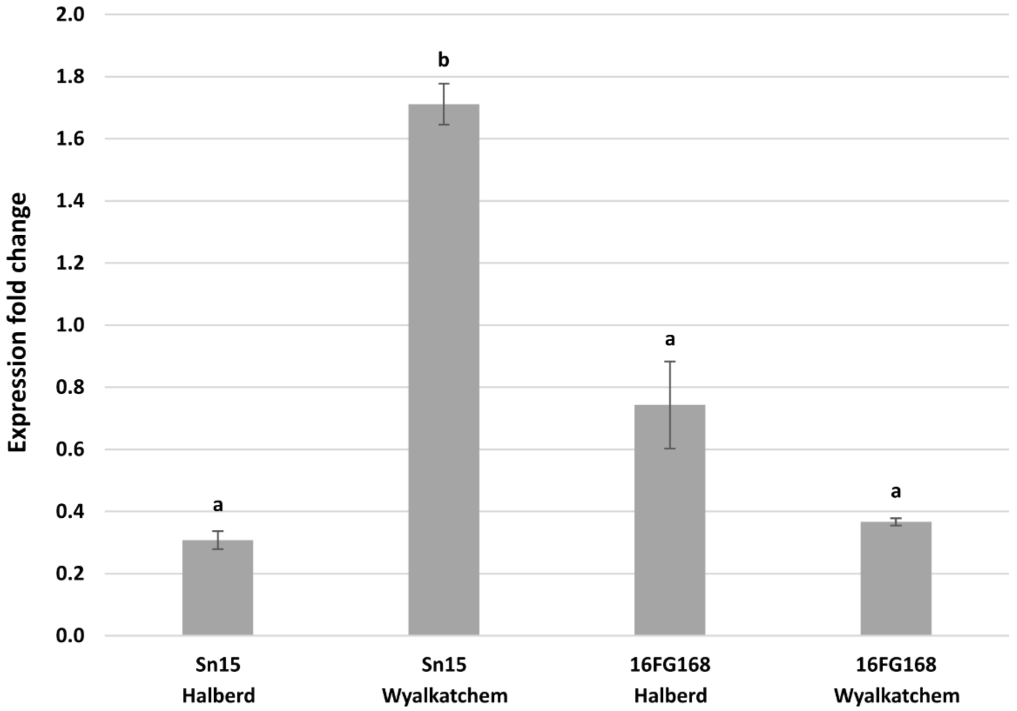

Supplementary Figure S2: Relative expression of SnTox3 in different conditions. Fold changes of SnTox3 transcript abundance compared to the negative control (gelatine spray) normalised with actin in the respective treatment (wheat line and isolate indicated) based on quantitative PCR using the ΔΔCt method. a and b denote significant expression differences.

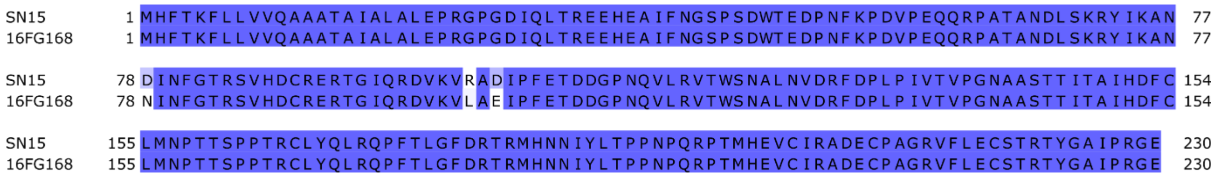

Supplementary Figure S3: Protein sequence isoforms of SnTox3 in five *Parastagonospora nodorum* isolates. SN15 represents isoform I which is also shared by WAC13404, WAC13443, and 15FG114. 16FG168 represents isoform II). This Figure was produced using Geneious Prime version 2020.2.4 (https://www.geneious.com).
