# Supplementary material for: GWAS analysis reveals distinct pathogenicity profiles of Australian Parastagonospora nodorum isolates and identification of marker-trait-associations to septoria nodorum blotch
Source: Sci Rep. 2021 May 12;11:10085. doi: 10.1038/s41598-021-87829-0 (PMC8115087; doi:10.1038/s41598-021-87829-0)
Supplement: Supplementary file 2 — Supplementary Table S1. [file 41598_2021_87829_MOESM2_ESM.pdf]

Supplemental Table S1: Wheat lines used in this study.

GWAS analysis reveals distinct pathogenicity profiles of Australian *Parastagonospora nodorum* isolates and identification of marker-trait-associations to septoria nodorum blotch

Huyen T.T Phan, Eiko Furuki, Lukas Hunziker, Kasia Rybak, Kar-Chun Tan

Supplementary Table S1: Wheat lines used in this study - CAIGE collection.

Qcode	Collection	GID	Pedigree
100:ZIF14	CAIGE 2016	400011823	QAFZAH-25/4/KAUZ'S'/3/TX62A4793-7/CD809//VEE'S'
100:ZIZ11	CAIGE 2013	4907584	CHORIZO//GH'S'/ANZA
103:ZIF14	CAIGE 2016	400001042	JAWAHIR-20 (CHECK)
104:ZIF14	CAIGE 2016	400011239	SEKSAKA-6/IZAZ-8
105:ZIF14	CAIGE 2016	400011238	SEKSAKA-6/IZAZ-8
105:ZIZ13	CAIGE 2015	400009811	HAMAM-5/OUKAB-2//TAZA-1
105:ZWW11	CAIGE 2013	6280059	EGA BONNIE ROCK/6/CPI8/GEDIZ/3/GOO//ALB/CRA/4/AE.SQUARROSA (208)/5/2*WESTONIA
106:ZIF14	CAIGE 2016	400011237	SERI.1B*2/3/KAUZ*2/BOW//KAUZ/4/KAUZ/FLORKWA-1
107:ZIF14	CAIGE 2016	400011236	SERI.1B*2/3/KAUZ*2/BOW//KAUZ/4/KAUZ/FLORKWA-1
108:ZWB11	CAIGE 2013	6178997	QUAIU/5/FRET2*2/4/SNI/TRAP#1/3/KAUZ*2/TRAP//KAUZ
10:ZIF14	CAIGE 2016	400011634	91-142 a 139//TAM200/KAUZ
111:ZIZ13	CAIGE 2015	400009030	ATENA-1/GAMDOW-3/3/MON'S'/ALD'S'//ALDAN'S'/IAS58
115:ZWW11	CAIGE 2013	6280103	PBW343*2/KUKUNA//WBLL1*2/KUKUNA
121:ZWB11	CAIGE 2013	6174903	ALTAR 84/AE.SQUARROSA (221)//3*BORL95/3/URES/JUN//KAUZ/4/WBLL1/5/MILAN/S87230//BAV92
122:ZIF14	CAIGE 2016	400011464	SHUHA-4//NS732/HER/3/TNMU/MILAN
122:ZWW11	CAIGE 2013	6280144	D67.2/P66.270//AE.SQUARROSA (320)/3/CUNNINGHAM/4/SKAUZ/BAV92
123:ZIF14	CAIGE 2016	400011463	SHUHA-4//NS732/HER/3/TNMU/MILAN
123:ZWB14	CAIGE 2016	6692023	BAVIS/4/MILAN/KAUZ//DHARWAR DRY/3/BAV92
124:ZIF14	CAIGE 2016	400011462	SHUHA-4//NS732/HER/3/TNMU/MILAN
124:ZWB11	CAIGE 2013	6177148	TRCH/6/HPO/TAN//VEE/3/2*PGO/4/MILAN/5/SSERI1
125:ZIF14	CAIGE 2016	400011461	SHUHA-4//NS732/HER/3/TNMU/MILAN
126:ZWB11	CAIGE 2013	6177174	PFAU/SERI.1B//AMAD/3/WAXWING/6/HPO/TAN//VEE/3/2*PGO/4/MILAN/5/SSERI1
129:ZIF14	CAIGE 2016	400011216	TEVEE'S'/SHUHA'S'//ICARDA-SRRL-7
12:ZIZ11	CAIGE 2013	400001765	VEE/PJN//2*KAUZ/3/PLK70/LIRA'S'//CNO79*2/PRL
135:ZWW11	CAIGE 2013	6280202	T.DICOCCON PI225332/AE.SQUARROSA (895)//WBLL1/3/2*WBLL1/4/SOKOLL
140:ZWB14	CAIGE 2016	6568597	MUTUS/2*DANPHE #1
151:ZWW11	CAIGE 2013	6280455	KRICHAUFF/2*PASTOR/4/MILAN/KAUZ//PRINIA/3/BAV92
172:ZWB11	CAIGE 2013	6175216	WAXWING/4/BL 1496/MILAN/3/CROC_1/AE.SQUARROSA (205)//KAUZ/5/FRNCLN
177:ZWW11	CAIGE 2013	6372296	KRICHAUFF/2*PASTOR//2*SOKOLL
17:ZWW11	CAIGE 2013	6278849	H45/4/KRICHAUFF/FINSI/3/URES/PRL//BAV92
182:ZWB11	CAIGE 2013	6175411	WAXWING*2/HEILO

190:ZWB14	CAIGE 2016	6692209	FALCIN/AE.SQUARROSA (312)/3/THB/CEP7780//SHA4/LIRA/4/FRET2/5/MUU/6/MILAN/KAUZ//DHARWAR DRY/3/BAV92
194:ZWB14	CAIGE 2016	6691422	SUNCO.6/FRAME//PASTOR/3/2*ATTILA/PASTOR
20:ZIF14	CAIGE 2016	400011314	CAR422/ANA//YACO/3/KAUZ*2/TRAP//KAUZ/4/BUCUR/5/BUCUR
21:ZIZ11	CAIGE 2013	400002150	HD2206/HORK'S'/3/2*NS732/HER//KAUZ'S'
21:ZWW11	CAIGE 2013	6278940	VORB/SOKOLL
22:ZWW11	CAIGE 2013	6278942	VORB/SOKOLL
23:ZWW11	CAIGE 2013	6278943	VORB/SOKOLL
24:ZIZ11	CAIGE 2013	5755188	SHA5//CARC/AUK/3/VEE#5//DOBUC'S'
24:ZIZ13	CAIGE 2015	400007763	TINAMOU-2//TEVEE-1/SHUHA-6
27:ZIF14	CAIGE 2016	400011494	DORADE-5/DUNAV
27:ZIZ11	CAIGE 2013	400001172	FOW-2//BAU/MILAN
284:ZWB13	CAIGE 2015	6691665	BAVIS*2//ATTILA/PASTOR
287:ZWB13	CAIGE 2015	6690931	SUNCO.6/FRAME//PASTOR/3/ATTILA/PASTOR/4/1447/PASTOR//KRICHAUFF
28:ZIZ11	CAIGE 2013	400000093	SHUHA-4//NS732/HER
29:ZIZ13	CAIGE 2015	400007826	NESSER/SERI//TEVEE-1/SHUHA-6
30:ZIZ11	CAIGE 2013	5791390	SHUHA-2/FOW-2
32:ZIZ13	CAIGE 2015	400008588	KARAWAN-1/TALLO 3//JADIDA-2
334:ZWB13	CAIGE 2015	6692306	SOKOLL/3/PASTOR//HXL7573/2*BAU/4/PARUS/PASTOR
33:ZIZ11	CAIGE 2013	400001814	FOW-2/PASTOR-2
34:ZWW11	CAIGE 2013	6279212	STYLET/4/SLVS/3/CROC_1/AE.SQUARROSA (224)//OPATA
37:ZIZ11	CAIGE 2013	400002091	NS732/HER//ARRIHANE/3/REGRAG-1
38:ZIZ11	CAIGE 2013	400002090	NS732/HER//ARRIHANE/3/REGRAG-1
42:ZIZ11	CAIGE 2013	400002026	ACHTAR*3//KANZ/KS85-8-4/3/ZEMAMRA-5
43:ZIF14	CAIGE 2016	400011629	IZAZ-2/3/ATTILA//VEE#5/DOBUC'S'/4/BOW #1/FENGKANG 15
44:ZIZ13	CAIGE 2015	400008575	TEVEE-1/STAR'S'/3/ACHTAR*3//KANZ/KS85-8-4
45:ZIZ11	CAIGE 2013	400002141	GARIBE//TURACO/CHIL/3/KATILA-11
45:ZIZ13	CAIGE 2015	400009700	SHUHA-7/SHUHA-14//MARHOUCH*4/SAADA
46:ZIF14	CAIGE 2016	400011290	JAWAHIR-1/3/PASTOR/SERI//PFAU
47:ZIF14	CAIGE 2016	400011289	JAWAHIR-1/3/PASTOR/SERI//PFAU
48:ZIF14	CAIGE 2016	400011288	JAWAHIR-1/3/PASTOR/SERI//PFAU
48:ZIZ11	CAIGE 2013	400001980	SISABAN-3
49:ZIF14	CAIGE 2016	400011287	JAWAHIR-1/3/PASTOR/SERI//PFAU
4:ZIZ11	CAIGE 2013	400001771	MILAN/SHA7/3/NS732/HER//SUDAN #11

51:ZIF14	CAIGE 2016	400011285	JAWAHIR-1/3/PASTOR/SERI//PFAU
52:ZIF14	CAIGE 2016	400011284	JAWAHIR-1/3/PASTOR/SERI//PFAU
52:ZWW11	CAIGE 2013	6279585	INQALAB 91*2/KUKUNA/4/TC14/2*HTG//DUCULA/3/PRINIA
55:ZIF14	CAIGE 2016	400011281	JAWAHIR-1/GIRWILL-5
55:ZIZ11	CAIGE 2013	-	WON-D 75/SHUHA-4
56:ZIZ13	CAIGE 2015	400002868	CHAM-4/SHUHA'S'/6/2*SAKER/5/RBS/ANZA/3/KVZ/HYS//YMH/TOB/4/BOW'S'
56:ZWB11	CAIGE 2013	6176308	ATTILA*2/PBW65*2//MURGA
56:ZWB13	CAIGE 2015	6681871	CHONTE//KIRITATI/2*TRCH
59:ZIF14	CAIGE 2016	400011278	JAWAHIR-1/GIRWILL-5
60:ZIF14	CAIGE 2016	400011277	JAWAHIR-1/GIRWILL-5
61:ZIF14	CAIGE 2016	400011276	JAWAHIR-1/GIRWILL-5
61:ZIZ11	CAIGE 2013	400002072	GOUBARA-1/HAAMA-1
62:ZWB14	CAIGE 2016	6565889	WBLL1*2/KURUKU//TACUPETO F2001*2/BRAMBLING
63:ZWB14	CAIGE 2016	6680725	QUAIU #1/BECARD
64:ZIZ11	CAIGE 2013	400002149	DIYAR-7
66:ZIF14	CAIGE 2016	400011738	JI5418/MARAS//SHARK/F4105W2.1
66:ZWB11	CAIGE 2013	6176409	ATTILA*2/PBW65*2//W485/HD29
6:ZIZ11	CAIGE 2013	400001994	MAYON'S'//CROW'S'/VEE'S'/3/NS732/HER
71:ZIZ11	CAIGE 2013	400002058	HAALA-37
76:ZIZ11	CAIGE 2013	400002188	AAFAQ-2
79:ZWB14	CAIGE 2016	6680917	BABAX/LR42//BABAX*2/3/PAVON 7S3, +LR47/4/ND643/2*WBLL1/5/BABAX/LR42//BABAX*2/3/PAVON 7S3, +LR47
7:ZIF14	CAIGE 2016	400011743	4WON-IR-257/5/YMH/HYS//HYS/TUR3055/3/DGA /4/ VPM / MOS
80:ZIF14	CAIGE 2016	400011259	KS85W663-2-4/3/VONA/W76-1141//THUNDERBIRD/6/JUP/4/CLLF/3/II14 53/ODIN//CI134431 /SEL 6425 /WA00477*2/5/CROC-1/AE.SQUARROSA (213)//PGO
80:ZWB11	CAIGE 2013	6176558	ATTILA*2/PBW65*2/4/BOW/NKT//CBRD/3/CBRD
82:ZIF14	CAIGE 2016	400011624	MON'S'/ALD'S'//TOWPE'S'/3/ZAIEM-10
85:ZIZ11	CAIGE 2013	400002178	WAHEED-1
88:ZIF14	CAIGE 2016	400011473	NS-307/87-461 a 63-690
8:ZIF14	CAIGE 2016	400011829	88ZHONG218//CTK/VEE/3/KVZ/GV//PR/4/KRASNOVODOPADSKAYA25/5/KS82117/MLT
90:ZIF14	CAIGE 2016	400011250	OPATA/RAYON//KAUZ/3/PFAU/MILAN
91:ZIZ13	CAIGE 2015	400007830	KAUZ//TRAP#1/BOW/3/QAFZAH-21
95:ZIF14	CAIGE 2016	400011471	PBW343*2/KUKUN//22SAWSN - 97
95:ZWB11	CAIGE 2013	6176914	MUNAL #1/FRANCOLIN #1
96:ZIF14	CAIGE 2016	400011470	PBW343*2/KUKUN//22SAWSN - 97

96:ZWB11	CAIGE 2013	6177828	FRNCLN/ROLF07
97:ZIF14	CAIGE 2016	4874402	PLK/LIRA/5/NAI60/3/14.53/ODIN//[CI13441]/4/GRK79/6/MNCH/7/CROC_1/AE.SQUARROSA (213)//PGO

Supplementary Table S1: Wheat lines used in this study - Vavilov collection.

Qcode	Year of registration in VIR	Origin Country
WLA-005	-	-
WLA-006	-	-
WLA-009	-	-
WLA-016	-	-
WLA-018	1958	Kazakhstan
WLA-104	1978	Russia
WLA-107	1977	Russia
WLA-110	1978	Russia
WLA-111	1977	Russia
WLA-115	-	Azerbaijan
WLA-118	-	Russia
WLA-124	-	Russia
WLA-125	-	Russia
WLA-135	1924	Russia
WLA-140	-	-
WLA-143	1941	USA
WLA-168	-	-
WLA-170	-	-
WLA-187	-	-
WLA-222	1989	Russia
WLA-223	1977	Kazakhstan
WLA-234	-	Armenia
WLA-235	-	Kyrgyzstan
WLA-242	-	Russia
WLA-259	-	-
WLA-266	-	Sweden
WLA-268	-	-
WLA-270	-	-
WLA-277	1933	Kazakhstan
WLA-293	-	Ukraine
WLA-294	1928	USA

WLA-301	1978	Russia
WLA-303	1986	Russia
WLA-304	1990	Russia
WLA-311	1930	China
