# Supplementary material for: GWAS analysis reveals distinct pathogenicity profiles of Australian Parastagonospora nodorum isolates and identification of marker-trait-associations to septoria nodorum blotch
Source: Sci Rep. 2021 May 12;11:10085. doi: 10.1038/s41598-021-87829-0 (PMC8115087; doi:10.1038/s41598-021-87829-0)
Supplement: Supplementary file 3 — Supplementary Table S2. [file 41598_2021_87829_MOESM3_ESM.pdf]

Supplemental Table S2: List of genes in the candidate regions spanning the SNB resistance QTLs and their functional annotations.

GWAS analysis reveals distinct pathogenicity profiles of Australian *Parastagonospora nodorum* isolates and identification of marker-trait-associations to septoria nodorum blotch

Huyen T.T Phan, Eiko Furuki, Lukas Hunziker, Kasia Rybak, Kar-Chun Tan

Table S2: List of genes in the candidate regions spanning the SNB resistance QTLs and their functional annotations.

Chromosome	Most significant SNP	Gene Id	Transcript	Description	Interpro Description
1B	AX.94950274	TraesCS1B02G339800	TraesCS1B02G339800.1		IPR032675:Leucine-rich repeat domain superfamily
		TraesCS1B02G340000	TraesCS1B02G340000.1		IPR001002:Chitin-binding, type 1 IPR018371:Chitin-binding, type 1, conserved site IPR036861:Endochitinase-like superfamily
		TraesCS1B02G340200	TraesCS1B02G340200.1	Agglutinin isolectin 3 (WGA3) (Fragment)	IPR001002:Chitin-binding, type 1 IPR018371:Chitin-binding, type 1, conserved site IPR036861:Endochitinase-like superfamily
	AX.94694976	TraesCS1D02G009406	TraesCS1D02G009406.1	Powdery mildew resistance protein (Fragment)	IPR032675:Leucine-rich repeat domain superfamily
		TraesCS1D02G009443	TraesCS1D02G009443.1	Powdery mildew resistance protein PM3 variant	IPR002182:NB-ARC IPR027417:P-loop containing nucleoside triphosphate hydrolase
		TraesCS1D02G009800	TraesCS1D02G009800.1	Protein DETOXIFICATION [Source:UniProtKB/TrEMBL;Acc:A0A1D5T4S3]	IPR002528:Multi antimicrobial extrusion protein
		TraesCS1D02G010000	TraesCS1D02G010000.1		IPR018865:Serine-threonine protein kinase 19
		TraesCS1D02G010000	TraesCS1D02G010000.2		IPR018865:Serine-threonine protein kinase 19
		TraesCS1D02G010100	TraesCS1D02G010100.1		IPR000719:Protein kinase domain IPR000742:EGF-like domain IPR001881:EGF-like calcium-binding domain IPR008271:Serine/threonine-protein kinase, active site IPR011009:Protein kinase-like domain superfamily IPR017441:Protein kinase, ATP binding site IPR018097:EGF-like calcium-binding, conserved site IPR025287:Wall-associated receptor kinase, galacturonan-binding domain
		TraesCS1D02G010800	TraesCS1D02G010800.1		IPR001611:Leucine-rich repeat IPR013210:Leucine-rich repeat-containing N-terminal, plant-type IPR032675:Leucine-rich repeat domain superfamily
		TraesCS1D02G010900	TraesCS1D02G010900.1	Serine/threonine-protein kinase [Source:UniProtKB/TrEMBL;Acc:A0A1D5T1A8]	IPR000719:Protein kinase domain IPR000858:S-locus glycoprotein domain IPR001480:Bulb-type lectin domain IPR003609:PAN/Apple domain IPR008271:Serine/threonine-protein kinase, active site IPR011009:Protein kinase-like domain superfamily IPR017441:Protein kinase, ATP binding site IPR024171:S-receptor-like serine/threonine-protein kinase IPR036426:Bulb-type lectin domain superfamily
		TraesCS1D02G011200	TraesCS1D02G011200.1		IPR002182:NB-ARC IPR027417:P-loop containing nucleoside triphosphate hydrolase IPR032675:Leucine-rich repeat domain superfamily IPR038005:Virus X resistance protein-like, coiled-coil domain IPR041118:Rx, N-terminal
		TraesCS1D02G011400	TraesCS1D02G011400.1		IPR032675:Leucine-rich repeat domain superfamily
		TraesCS1D02G012200	TraesCS1D02G012200.1		IPR001810:F-box domain IPR015915:Kelch-type beta propeller IPR017451:F-box associated interaction domain IPR036047:F-box-like domain superfamily
		TraesCS1D02G012800	TraesCS1D02G012800.1		IPR000719:Protein kinase domain IPR000858:S-locus glycoprotein domain IPR001480:Bulb-type lectin domain IPR003609:PAN/Apple domain IPR008271:Serine/threonine-protein kinase, active site IPR011009:Protein kinase-like domain superfamily IPR017441:Protein kinase, ATP binding site IPR024171:S-receptor-like serine/threonine-protein kinase IPR036426:Bulb-type lectin domain superfamily
		TraesCS1D02G012900	TraesCS1D02G012900.1	Serine/threonine-protein kinase [Source:UniProtKB/TrEMBL;Acc:A0A1D5T2E8]	IPR000719:Protein kinase domain IPR000858:S-locus glycoprotein domain IPR001480:Bulb-type lectin domain IPR003609:PAN/Apple domain IPR008271:Serine/threonine-protein kinase, active site IPR011009:Protein kinase-like domain superfamily IPR017441:Protein kinase, ATP binding site IPR024171:S-receptor-like serine/threonine-protein kinase IPR036426:Bulb-type lectin domain superfamily

1D	TraesCS1D02G013100	TraesCS1D02G013100.1	Tubulin-folding cofactor E [Source:Projected from Arabidopsis thaliana (AT1G71440) UniProtKB/Swiss-Prot;Acc:Q8GRL7]	IPR001611:Leucine-rich repeat IPR003591:Leucine-rich repeat, typical subtype IPR029071:Ubiquitin-like domain superfamily IPR032675:Leucine-rich repeat domain superfamily
	TraesCS1D02G013800	TraesCS1D02G013800.1	Serine/threonine-protein kinase [Source:UniProtKB/TrEMBL;Acc:A0A1D5T2M5]	IPR000719:Protein kinase domain IPR000858:S-locus glycoprotein domain IPR001480:Bulb-type lectin domain IPR003609:PAN/Apple domain IPR008271:Serine/threonine-protein kinase, active site IPR011009:Protein kinase-like domain superfamily IPR017441:Protein kinase, ATP binding site IPR024171:S-receptor-like serine/threonine-protein kinase IPR036426:Bulb-type lectin domain superfamily
	TraesCS1D02G014000	TraesCS1D02G014000.1		IPR011009:Protein kinase-like domain superfamily
	TraesCS1D02G014300	TraesCS1D02G014300.1		IPR000719:Protein kinase domain IPR003609:PAN/Apple domain IPR008271:Serine/threonine-protein kinase, active site IPR011009:Protein kinase-like domain superfamily IPR017441:Protein kinase, ATP binding site
	TraesCS1D02G014600	TraesCS1D02G014600.1		IPR002182:NB-ARC IPR027417:P-loop containing nucleoside triphosphate hydrolase IPR032675:Leucine-rich repeat domain superfamily IPR038005:Virus X resistance protein-like, coiled-coil domain IPR041118:Rx, N-terminal
	TraesCS1D02G014700	TraesCS1D02G014700.1		IPR032675:Leucine-rich repeat domain superfamily
	TraesCS1D02G015300	TraesCS1D02G015300.1	Receptor-like kinase 2 [Source:UniProtKB/TrEMBL;Acc:D0QEJ4] Receptor-like kinase 2	IPR000719:Protein kinase domain IPR008271:Serine/threonine-protein kinase, active site IPR011009:Protein kinase-like domain superfamily IPR017441:Protein kinase, ATP binding site
	TraesCS1D02G015400	TraesCS1D02G015400.1		IPR000719:Protein kinase domain IPR008271:Serine/threonine-protein kinase, active site IPR011009:Protein kinase-like domain superfamily IPR017441:Protein kinase, ATP binding site
	TraesCS1D02G015412	TraesCS1D02G015412.1		IPR002182:NB-ARC IPR027417:P-loop containing nucleoside triphosphate hydrolase
	TraesCS1D02G015500	TraesCS1D02G015500.1		IPR002182:NB-ARC IPR027417:P-loop containing nucleoside triphosphate hydrolase IPR032675:Leucine-rich repeat domain superfamily IPR038005:Virus X resistance protein-like, coiled-coil domain IPR041118:Rx, N-terminal
	TraesCS1D02G015800	TraesCS1D02G015800.1		IPR000719:Protein kinase domain IPR008271:Serine/threonine-protein kinase, active site IPR011009:Protein kinase-like domain superfamily IPR017441:Protein kinase, ATP binding site
	TraesCS1D02G015800	TraesCS1D02G015800.2		IPR000719:Protein kinase domain IPR008271:Serine/threonine-protein kinase, active site IPR011009:Protein kinase-like domain superfamily IPR017441:Protein kinase, ATP binding site
	TraesCS1D02G015900	TraesCS1D02G015900.1		IPR000719:Protein kinase domain IPR008271:Serine/threonine-protein kinase, active site IPR011009:Protein kinase-like domain superfamily IPR017441:Protein kinase, ATP binding site
	TraesCS1D02G016026	TraesCS1D02G016026.1	LRR14 [Source:UniProtKB/TrEMBL;Acc:Q9ATQ3] LRR14	IPR002182:NB-ARC IPR027417:P-loop containing nucleoside triphosphate hydrolase IPR032675:Leucine-rich repeat domain superfamily IPR038005:Virus X resistance protein-like, coiled-coil domain IPR041118:Rx, N-terminal
	TraesCS1D02G016100	TraesCS1D02G016100.1		IPR002182:NB-ARC IPR027417:P-loop containing nucleoside triphosphate hydrolase IPR032675:Leucine-rich repeat domain superfamily IPR038005:Virus X resistance protein-like, coiled-coil domain IPR041118:Rx, N-terminal
	TraesCS1D02G016400	TraesCS1D02G016400.1		IPR001611:Leucine-rich repeat IPR032675:Leucine-rich repeat domain superfamily

TraesCS1D02G016900	TraesCS1D02G016900.1		IPR002182:NB-ARC IPR027417:P-loop containing nucleoside triphosphate hydrolase IPR032675:Leucine-rich repeat domain superfamily IPR038005:Virus X resistance protein-like, coiled-coil domain IPR041118:Rx, N-terminal
TraesCS1D02G016983	TraesCS1D02G016983.1	Putative non-tir-NBS-LRR resistance gene analog (Fragment)	IPR002182:NB-ARC IPR027417:P-loop containing nucleoside triphosphate hydrolase
TraesCS1D02G017400	TraesCS1D02G017400.1		IPR002182:NB-ARC IPR027417:P-loop containing nucleoside triphosphate hydrolase IPR032675:Leucine-rich repeat domain superfamily IPR038005:Virus X resistance protein-like, coiled-coil domain IPR041118:Rx, N-terminal
TraesCS1D02G017600	TraesCS1D02G017600.1		IPR002182:NB-ARC IPR027417:P-loop containing nucleoside triphosphate hydrolase IPR032675:Leucine-rich repeat domain superfamily IPR038005:Virus X resistance protein-like, coiled-coil domain IPR041118:Rx, N-terminal
TraesCS1D02G017700	TraesCS1D02G017700.1		IPR000719:Protein kinase domain IPR008271:Serine/threonine-protein kinase, active site IPR011009:Protein kinase-like domain superfamily IPR017441:Protein kinase, ATP binding site
TraesCS1D02G017800	TraesCS1D02G017800.1		IPR000719:Protein kinase domain IPR008271:Serine/threonine-protein kinase, active site IPR011009:Protein kinase-like domain superfamily IPR017441:Protein kinase, ATP binding site
TraesCS1D02G017900	TraesCS1D02G017900.1		IPR000719:Protein kinase domain IPR008271:Serine/threonine-protein kinase, active site IPR011009:Protein kinase-like domain superfamily IPR017441:Protein kinase, ATP binding site
TraesCS1D02G018000	TraesCS1D02G018000.2		IPR000719:Protein kinase domain IPR001245:Serine-threonine/tyrosine-protein kinase, catalytic domain IPR011009:Protein kinase-like domain superfamily IPR017441:Protein kinase, ATP binding site IPR020635:Tyrosine-protein kinase, catalytic domain
TraesCS1D02G018000	TraesCS1D02G018000.1		IPR000719:Protein kinase domain IPR008271:Serine/threonine-protein kinase, active site IPR011009:Protein kinase-like domain superfamily
TraesCS1D02G018700	TraesCS1D02G018700.2		IPR002182:NB-ARC IPR027417:P-loop containing nucleoside triphosphate hydrolase IPR041118:Rx, N-terminal
TraesCS1D02G018700	TraesCS1D02G018700.1		IPR002182:NB-ARC IPR027417:P-loop containing nucleoside triphosphate hydrolase IPR041118:Rx, N-terminal
TraesCS1D02G018800	TraesCS1D02G018800.1		IPR002182:NB-ARC IPR027417:P-loop containing nucleoside triphosphate hydrolase IPR032675:Leucine-rich repeat domain superfamily
TraesCS1D02G019400	TraesCS1D02G019400.1		IPR001128:Cytochrome P450 IPR002401:Cytochrome P450, E-class, group I IPR017972:Cytochrome P450, conserved site IPR036396:Cytochrome P450 superfamily
TraesCS1D02G019600	TraesCS1D02G019600.1	Name: Lr10	IPR032675:Leucine-rich repeat domain superfamily
TraesCS1D02G019700	TraesCS1D02G019700.1		IPR002182:NB-ARC IPR027417:P-loop containing nucleoside triphosphate hydrolase IPR032675:Leucine-rich repeat domain superfamily IPR038005:Virus X resistance protein-like, coiled-coil domain IPR041118:Rx, N-terminal
TraesCS1D02G020400	TraesCS1D02G020400.1		IPR001128:Cytochrome P450 IPR002401:Cytochrome P450, E-class, group I IPR017972:Cytochrome P450, conserved site IPR036396:Cytochrome P450 superfamily
TraesCS1D02G020619	TraesCS1D02G020619.1		IPR032675:Leucine-rich repeat domain superfamily
TraesCS1D02G021000	TraesCS1D02G021000.1		IPR002182:NB-ARC IPR027417:P-loop containing nucleoside triphosphate hydrolase IPR038005:Virus X resistance protein-like, coiled-coil domain IPR041118:Rx, N-terminal
TraesCS1D02G021200	TraesCS1D02G021200.1		IPR002182:NB-ARC IPR027417:P-loop containing nucleoside triphosphate hydrolase
TraesCS1D02G022600	TraesCS1D02G022600.1		IPR001128:Cytochrome P450 IPR002401:Cytochrome P450, E-class, group I IPR017972:Cytochrome P450, conserved site IPR036396:Cytochrome P450 superfamily

		TraesCS1D02G023000	TraesCS1D02G023000.1	IPR001611:Leucine-rich repeat IPR003591:Leucine-rich repeat, typical subtype IPR032675:Leucine-rich repeat domain superfamily
		TraesCS1D02G023100	TraesCS1D02G023100.1	IPR003591:Leucine-rich repeat, typical subtype IPR032675:Leucine-rich repeat domain superfamily
	AX.95253275	TraesCS1D02G146700	TraesCS1D02G146700.1	IPR000719:Protein kinase domain IPR001611:Leucine-rich repeat IPR003591:Leucine-rich repeat, typical subtype IPR008266:Tyrosine-protein kinase, active site IPR011009:Protein kinase-like domain superfamily IPR013210:Leucine-rich repeat-containing N-terminal, plant-type IPR017441:Protein kinase, ATP binding site IPR032675:Leucine-rich repeat domain superfamily
1D		TraesCS1D02G146800	TraesCS1D02G146800.1	IPR000719:Protein kinase domain IPR001611:Leucine-rich repeat IPR003591:Leucine-rich repeat, typical subtype IPR008266:Tyrosine-protein kinase, active site IPR011009:Protein kinase-like domain superfamily IPR013210:Leucine-rich repeat-containing N-terminal, plant-type IPR017441:Protein kinase, ATP binding site IPR032675:Leucine-rich repeat domain superfamily
		TraesCS1D02G146900	TraesCS1D02G146900.1	IPR000719:Protein kinase domain IPR001611:Leucine-rich repeat IPR003591:Leucine-rich repeat, typical subtype IPR008266:Tyrosine-protein kinase, active site IPR011009:Protein kinase-like domain superfamily IPR013210:Leucine-rich repeat-containing N-terminal, plant-type IPR017441:Protein kinase, ATP binding site IPR032675:Leucine-rich repeat domain superfamily
		TraesCS1D02G149200	TraesCS1D02G149200.1	IPR002182:NB-ARC IPR027417:P-loop containing nucleoside triphosphate hydrolase IPR032675:Leucine-rich repeat domain superfamily
	AX.94723571	TraesCS1D02G264600	TraesCS1D02G264600.1	IPR000719:Protein kinase domain IPR008271:Serine/threonine-protein kinase, active site IPR011009:Protein kinase-like domain superfamily IPR017441:Protein kinase, ATP binding site
		TraesCS1D02G264800	TraesCS1D02G264800.1	IPR001128:Cytochrome P450 IPR002401:Cytochrome P450, E-class, group I IPR017972:Cytochrome P450, conserved site IPR036396:Cytochrome P450 superfamily
		TraesCS1D02G265100	TraesCS1D02G265100.1	IPR001611:Leucine-rich repeat IPR006553:Leucine-rich repeat, cysteine-containing subtype IPR032675:Leucine-rich repeat domain superfamily
		TraesCS1D02G265200	TraesCS1D02G265200.2	IPR011009:Protein kinase-like domain superfamily IPR015275:Actin-fragmin kinase, catalytic domain IPR035010:Dual specificity protein phosphatase PHS1 IPR036940:Phosphatidylinositol 3-/4-kinase, catalytic domain superfamily
1D		TraesCS1D02G265200	TraesCS1D02G265200.4	IPR011009:Protein kinase-like domain superfamily IPR015275:Actin-fragmin kinase, catalytic domain IPR035010:Dual specificity protein phosphatase PHS1 IPR036940:Phosphatidylinositol 3-/4-kinase, catalytic domain superfamily
		TraesCS1D02G265200	TraesCS1D02G265200.1	IPR011009:Protein kinase-like domain superfamily IPR015275:Actin-fragmin kinase, catalytic domain IPR029021:Protein-tyrosine phosphatase-like IPR035010:Dual specificity protein phosphatase PHS1 IPR036940:Phosphatidylinositol 3-/4-kinase, catalytic domain superfamily
		TraesCS1D02G265200	TraesCS1D02G265200.3	IPR011009:Protein kinase-like domain superfamily IPR015275:Actin-fragmin kinase, catalytic domain IPR035010:Dual specificity protein phosphatase PHS1 IPR036940:Phosphatidylinositol 3-/4-kinase, catalytic domain superfamily
		TraesCS1D02G267200	TraesCS1D02G267200.1	IPR002182:NB-ARC IPR027417:P-loop containing nucleoside triphosphate hydrolase IPR032675:Leucine-rich repeat domain superfamily
		TraesCS1D02G268400	TraesCS1D02G268400.1	IPR001810:F-box domain IPR006566:FBD domain IPR032675:Leucine-rich repeat domain superfamily IPR036047:F-box-like domain superfamily

2A	AX.95632742	TraesCS2A02G418200	TraesCS2A02G418200.1		IPR000719:Protein kinase domain IPR006311:Twin-arginine translocation pathway, signal sequence IPR008271:Serine/threonine-protein kinase, active site IPR011009:Protein kinase-like domain superfamily IPR017441:Protein kinase, ATP binding site IPR021720:Malectin domain IPR032675:Leucine-rich repeat domain superfamily
		TraesCS2A02G418300	TraesCS2A02G418300.1		IPR000719:Protein kinase domain IPR008271:Serine/threonine-protein kinase, active site IPR011009:Protein kinase-like domain superfamily IPR017441:Protein kinase, ATP binding site IPR021720:Malectin domain IPR032675:Leucine-rich repeat domain superfamily
		TraesCS2A02G418400	TraesCS2A02G418400.1		IPR000719:Protein kinase domain IPR008271:Serine/threonine-protein kinase, active site IPR011009:Protein kinase-like domain superfamily IPR017441:Protein kinase, ATP binding site IPR021720:Malectin domain IPR032675:Leucine-rich repeat domain superfamily
		TraesCS2A02G421000	TraesCS2A02G421000.1		IPR001611:Leucine-rich repeat IPR002182:NB-ARC IPR027417:P-loop containing nucleoside triphosphate hydrolase IPR032675:Leucine-rich repeat domain superfamily IPR036388:Winged helix-like DNA-binding domain superfamily IPR038005:Virus X resistance protein-like, coiled-coil domain IPR041118:Rx, N-terminal
		TraesCS2A02G422300	TraesCS2A02G422300.1		IPR001128:Cytochrome P450 IPR002401:Cytochrome P450, E-class, group I IPR017972:Cytochrome P450, conserved site IPR036396:Cytochrome P450 superfamily
		TraesCS2A02G423000	TraesCS2A02G423000.1		IPR001810:F-box domain IPR036047:F-box-like domain superfamily
		TraesCS2A02G423800	TraesCS2A02G423800.1		IPR001611:Leucine-rich repeat IPR013210:Leucine-rich repeat-containing N-terminal, plant-type IPR032675:Leucine-rich repeat domain superfamily
	AX.95171319	TraesCS2A02G555900	TraesCS2A02G555900.2		IPR001810:F-box domain IPR006566:FBD domain IPR032675:Leucine-rich repeat domain superfamily IPR036047:F-box-like domain superfamily
		TraesCS2A02G558000	TraesCS2A02G558000.1	WAMP-3.1, antimicrobial peptide [Source:UniProtKB/TrEMBL;Acc:H6S4F9] WAMP-3.1, antimicrobial peptide	IPR001002:Chitin-binding, type 1 IPR036861:Endochitinase-like superfamily
		TraesCS2A02G558500	TraesCS2A02G558500.1		IPR000719:Protein kinase domain IPR008271:Serine/threonine-protein kinase, active site IPR011009:Protein kinase-like domain superfamily IPR017441:Protein kinase, ATP binding site
		TraesCS2A02G559000	TraesCS2A02G559000.1		IPR000719:Protein kinase domain IPR008271:Serine/threonine-protein kinase, active site IPR011009:Protein kinase-like domain superfamily IPR017441:Protein kinase, ATP binding site
		TraesCS2A02G559400	TraesCS2A02G559400.1		IPR000719:Protein kinase domain IPR001245:Serine-threonine/tyrosine-protein kinase, catalytic domain IPR001611:Leucine-rich repeat IPR003591:Leucine-rich repeat, typical subtype IPR008271:Serine/threonine-protein kinase, active site IPR011009:Protein kinase-like domain superfamily IPR013210:Leucine-rich repeat-containing N-terminal, plant-type IPR017441:Protein kinase, ATP binding site IPR032675:Leucine-rich repeat domain superfamily
		TraesCS2A02G560600	TraesCS2A02G560600.1		IPR002182:NB-ARC IPR027417:P-loop containing nucleoside triphosphate hydrolase IPR032675:Leucine-rich repeat domain superfamily IPR041118:Rx, N-terminal
		TraesCS2A02G560700	TraesCS2A02G560700.2		IPR002182:NB-ARC IPR027417:P-loop containing nucleoside triphosphate hydrolase IPR032675:Leucine-rich repeat domain superfamily IPR041118:Rx, N-terminal
		TraesCS2A02G560700	TraesCS2A02G560700.1		IPR002182:NB-ARC IPR027417:P-loop containing nucleoside triphosphate hydrolase IPR032675:Leucine-rich repeat domain superfamily IPR041118:Rx, N-terminal
		TraesCS2A02G560900	TraesCS2A02G560900.1		IPR002182:NB-ARC IPR027417:P-loop containing nucleoside triphosphate hydrolase IPR032675:Leucine-rich repeat domain superfamily IPR041118:Rx, N-terminal

2A		TraesCS2A02G561100	TraesCS2A02G561100.1	IPR002182:NB-ARC IPR027417:P-loop containing nucleoside triphosphate hydrolase
		TraesCS2A02G562200	TraesCS2A02G562200.1	IPR001128:Cytochrome P450 IPR002401:Cytochrome P450, E-class, group I IPR017972:Cytochrome P450, conserved site IPR036396:Cytochrome P450 superfamily
		TraesCS2A02G562800	TraesCS2A02G562800.1	IPR000719:Protein kinase domain IPR001245:Serine-threonine/tyrosine-protein kinase, catalytic domain IPR002902:Gnk2-homologous domain IPR008271:Serine/threonine-protein kinase, active site IPR011009:Protein kinase-like domain superfamily IPR038408:Gnk2-homologous domain superfamily
		TraesCS2A02G563200	TraesCS2A02G563200.1	IPR032675:Leucine-rich repeat domain superfamily
		TraesCS2A02G563200	TraesCS2A02G563200.2	IPR032675:Leucine-rich repeat domain superfamily
		TraesCS2A02G563400	TraesCS2A02G563400.2	IPR000719:Protein kinase domain IPR008271:Serine/threonine-protein kinase, active site IPR011009:Protein kinase-like domain superfamily IPR017441:Protein kinase, ATP binding site IPR021094:NPR1/NIM1-like, C-terminal
		TraesCS2A02G563400	TraesCS2A02G563400.1	IPR000719:Protein kinase domain IPR008271:Serine/threonine-protein kinase, active site IPR011009:Protein kinase-like domain superfamily IPR017441:Protein kinase, ATP binding site IPR021094:NPR1/NIM1-like, C-terminal
		TraesCS2B02G158700	TraesCS2B02G158700.1	IPR000719:Protein kinase domain IPR001220:Legume lectin domain IPR008271:Serine/threonine-protein kinase, active site IPR011009:Protein kinase-like domain superfamily IPR013320:Concanavalin A-like lectin/glucanase domain superfamily IPR017441:Protein kinase, ATP binding site
		TraesCS2B02G158800	TraesCS2B02G158800.1	IPR000719:Protein kinase domain IPR001220:Legume lectin domain IPR008271:Serine/threonine-protein kinase, active site IPR011009:Protein kinase-like domain superfamily IPR013320:Concanavalin A-like lectin/glucanase domain superfamily IPR017441:Protein kinase, ATP binding site
		TraesCS2B02G163000	TraesCS2B02G163000.1	IPR000719:Protein kinase domain IPR008271:Serine/threonine-protein kinase, active site IPR011009:Protein kinase-like domain superfamily
		TraesCS2B02G163700	TraesCS2B02G163700.1	IPR001810:F-box domain IPR036047:F-box-like domain superfamily
	AX.94774467	TraesCS2B02G220900	TraesCS2B02G220900.1	IPR000719:Protein kinase domain IPR001220:Legume lectin domain IPR008271:Serine/threonine-protein kinase, active site IPR011009:Protein kinase-like domain superfamily IPR013320:Concanavalin A-like lectin/glucanase domain superfamily IPR017441:Protein kinase, ATP binding site
		TraesCS2B02G221100	TraesCS2B02G221100.1	IPR000719:Protein kinase domain IPR001220:Legume lectin domain IPR008271:Serine/threonine-protein kinase, active site IPR011009:Protein kinase-like domain superfamily IPR013320:Concanavalin A-like lectin/glucanase domain superfamily IPR017441:Protein kinase, ATP binding site
		TraesCS2B02G221200	TraesCS2B02G221200.1	IPR000719:Protein kinase domain IPR001220:Legume lectin domain IPR008271:Serine/threonine-protein kinase, active site IPR011009:Protein kinase-like domain superfamily IPR013320:Concanavalin A-like lectin/glucanase domain superfamily IPR017441:Protein kinase, ATP binding site IPR019825:Legume lectin, beta chain, Mn/Ca-binding site
2B		TraesCS2B02G222100	TraesCS2B02G222100.1	IPR000719:Protein kinase domain IPR011009:Protein kinase-like domain superfamily IPR013210:Leucine-rich repeat-containing N-terminal, plant-type IPR017441:Protein kinase, ATP binding site IPR032675:Leucine-rich repeat domain superfamily

		TraesCS2B02G222800	TraesCS2B02G222800.1		IPR002182:NB-ARC IPR027417:P-loop containing nucleoside triphosphate hydrolase IPR032675:Leucine-rich repeat domain superfamily IPR038005:Virus X resistance protein-like, coiled-coil domain IPR041118:Rx, N-terminal
		TraesCS2B02G222900	TraesCS2B02G222900.1		IPR000719:Protein kinase domain IPR011009:Protein kinase-like domain superfamily IPR017441:Protein kinase, ATP binding site
		TraesCS2B02G223600	TraesCS2B02G223600.2		IPR000719:Protein kinase domain IPR008271:Serine/threonine-protein kinase, active site IPR011009:Protein kinase-like domain superfamily
		TraesCS2B02G223600	TraesCS2B02G223600.1		IPR000719:Protein kinase domain IPR008271:Serine/threonine-protein kinase, active site IPR011009:Protein kinase-like domain superfamily
		TraesCS2B02G225000	TraesCS2B02G225000.1		IPR002182:NB-ARC IPR027417:P-loop containing nucleoside triphosphate hydrolase IPR032675:Leucine-rich repeat domain superfamily IPR038005:Virus X resistance protein-like, coiled-coil domain IPR041118:Rx, N-terminal
	AX.95165387	TraesCS2B02G549700	TraesCS2B02G549700.1		IPR001128:Cytochrome P450 IPR002401:Cytochrome P450, E-class, group I IPR017972:Cytochrome P450, conserved site IPR036396:Cytochrome P450 superfamily
		TraesCS2B02G551900	TraesCS2B02G551900.1		IPR001810:F-box domain IPR036047:F-box-like domain superfamily
		TraesCS2B02G552500	TraesCS2B02G552500.1		IPR000719:Protein kinase domain IPR008271:Serine/threonine-protein kinase, active site IPR011009:Protein kinase-like domain superfamily IPR017441:Protein kinase, ATP binding site
		TraesCS2B02G552500	TraesCS2B02G552500.2		IPR000719:Protein kinase domain IPR008271:Serine/threonine-protein kinase, active site IPR011009:Protein kinase-like domain superfamily IPR020635:Tyrosine-protein kinase, catalytic domain
		TraesCS2B02G552900	TraesCS2B02G552900.1		IPR001810:F-box domain IPR013187:F-box associated domain, type 3 IPR036047:F-box-like domain superfamily
2B		TraesCS2B02G553000	TraesCS2B02G553000.1		IPR007320:Programmed cell death protein 2, C-terminal
		TraesCS2B02G553200	TraesCS2B02G553200.1		IPR013187:F-box associated domain, type 3 IPR017451:F-box associated interaction domain
		TraesCS2B02G554700	TraesCS2B02G554700.1		IPR000719:Protein kinase domain IPR000858:S-locus glycoprotein domain IPR001480:Bulb-type lectin domain IPR003609:PAN/Apple domain IPR008271:Serine/threonine-protein kinase, active site IPR011009:Protein kinase-like domain superfamily IPR017441:Protein kinase, ATP binding site IPR024171:S-receptor-like serine/threonine-protein kinase IPR036426:Bulb-type lectin domain superfamily
		TraesCS2B02G555200	TraesCS2B02G555200.1		IPR000719:Protein kinase domain IPR011009:Protein kinase-like domain superfamily IPR017441:Protein kinase, ATP binding site
		TraesCS2B02G555200	TraesCS2B02G555200.2		IPR000719:Protein kinase domain IPR001245:Serine-threonine/tyrosine-protein kinase, catalytic domain IPR011009:Protein kinase-like domain superfamily IPR017441:Protein kinase, ATP binding site
	AX.95133018	TraesCS2D02G561100	TraesCS2D02G561100.1		IPR001611:Leucine-rich repeat IPR003591:Leucine-rich repeat, typical subtype IPR013210:Leucine-rich repeat-containing N-terminal, plant-type IPR032675:Leucine-rich repeat domain superfamily
		TraesCS2D02G561200	TraesCS2D02G561200.1		IPR000719:Protein kinase domain IPR001245:Serine-threonine/tyrosine-protein kinase, catalytic domain IPR001611:Leucine-rich repeat IPR008271:Serine/threonine-protein kinase, active site IPR011009:Protein kinase-like domain superfamily IPR017441:Protein kinase, ATP binding site IPR025875:Leucine rich repeat 4 IPR032675:Leucine-rich repeat domain superfamily
		TraesCS2D02G563800	TraesCS2D02G563800.1	Uncharacterized protein	IPR032675:Leucine-rich repeat domain superfamily
		TraesCS2D02G563900	TraesCS2D02G563900.1	Uncharacterized protein	IPR001128:Cytochrome P450 IPR002401:Cytochrome P450, E-class, group I IPR017972:Cytochrome P450, conserved site IPR036396:Cytochrome P450 superfamily

2D	TraesCS2D02G564300	TraesCS2D02G564300.1	Protein kinase domain-containing protein	IPR000719:Protein kinase domain IPR008271:Serine/threonine-protein kinase, active site IPR011009:Protein kinase-like domain superfamily IPR017441:Protein kinase, ATP binding site
	TraesCS2D02G566800	TraesCS2D02G566800.1	Peroxidase [Source:UniProtKB/TrEMBL;Acc:A0A1D5UJX6] Peroxidase (EC 1.11.1.7)	IPR000823:Plant peroxidase IPR002016:Haem peroxidase IPR010255:Haem peroxidase superfamily IPR019793:Peroxidases heam-ligand binding site IPR019794:Peroxidase, active site IPR033905:Secretory peroxidase
	TraesCS2D02G566900	TraesCS2D02G566900.1	Cytochrome c domain-containing protein	IPR002327:Cytochrome c, class IA/ IB IPR009056:Cytochrome c-like domain IPR036909:Cytochrome c-like domain superfamily
	TraesCS2D02G567200	TraesCS2D02G567200.1	NB-ARC domain-containing protein	IPR002182:NB-ARC IPR003591:Leucine-rich repeat, typical subtype IPR027417:P-loop containing nucleoside triphosphate hydrolase IPR032675:Leucine-rich repeat domain superfamily
	TraesCS2D02G567300	TraesCS2D02G567300.1		IPR032675:Leucine-rich repeat domain superfamily
	TraesCS2D02G567400	TraesCS2D02G567400.1	NB-ARC domain-containing protein	IPR002182:NB-ARC IPR027417:P-loop containing nucleoside triphosphate hydrolase
	TraesCS2D02G567500	TraesCS2D02G567500.1	Uncharacterized protein	IPR002182:NB-ARC IPR003591:Leucine-rich repeat, typical subtype IPR027417:P-loop containing nucleoside triphosphate hydrolase IPR032675:Leucine-rich repeat domain superfamily IPR038005:Virus X resistance protein-like, coiled-coil domain IPR041118:Rx, N-terminal
	TraesCS2D02G567800	TraesCS2D02G567800.1		IPR032675:Leucine-rich repeat domain superfamily IPR038005:Virus X resistance protein-like, coiled-coil domain IPR041118:Rx, N-terminal
	TraesCS2D02G568700	TraesCS2D02G568700.1		IPR002182:NB-ARC IPR027417:P-loop containing nucleoside triphosphate hydrolase IPR032675:Leucine-rich repeat domain superfamily
	TraesCS2D02G568800	TraesCS2D02G568800.1		IPR032675:Leucine-rich repeat domain superfamily
	TraesCS2D02G568900	TraesCS2D02G568900.1		IPR032675:Leucine-rich repeat domain superfamily
	TraesCS2D02G569100	TraesCS2D02G569100.1		IPR001611:Leucine-rich repeat IPR024788:Malectin-like domain IPR032675:Leucine-rich repeat domain superfamily
	TraesCS2D02G569400	TraesCS2D02G569400.1		IPR002182:NB-ARC IPR027417:P-loop containing nucleoside triphosphate hydrolase IPR032675:Leucine-rich repeat domain superfamily IPR041118:Rx, N-terminal
	TraesCS2D02G570200	TraesCS2D02G570200.1		IPR036047:F-box-like domain superfamily
	TraesCS2D02G571100	TraesCS2D02G571100.1		IPR000719:Protein kinase domain IPR001245:Serine-threonine/tyrosine-protein kinase, catalytic domain IPR001611:Leucine-rich repeat IPR003591:Leucine-rich repeat, typical subtype IPR008271:Serine/threonine-protein kinase, active site IPR011009:Protein kinase-like domain superfamily IPR013210:Leucine-rich repeat-containing N-terminal, plant-type IPR017441:Protein kinase, ATP binding site IPR032675:Leucine-rich repeat domain superfamily
	TraesCS2D02G571600	TraesCS2D02G571600.1	WAMP-1, antimicrobial peptide [Source:UniProtKB/TrEMBL;Acc:H6S4F8] WAMP-1, antimicrobial peptide	IPR001002:Chitin-binding, type 1 IPR036861:Endochitinase-like superfamily
	TraesCS2D02G572000	TraesCS2D02G572000.2		IPR000719:Protein kinase domain IPR008271:Serine/threonine-protein kinase, active site IPR011009:Protein kinase-like domain superfamily IPR017441:Protein kinase, ATP binding site IPR021094:NPR1/NIM1-like, C-terminal
	TraesCS2D02G572000	TraesCS2D02G572000.1		IPR000719:Protein kinase domain IPR008271:Serine/threonine-protein kinase, active site IPR011009:Protein kinase-like domain superfamily IPR017441:Protein kinase, ATP binding site IPR021094:NPR1/NIM1-like, C-terminal

		TraesCS2D02G572600	TraesCS2D02G572600.1		IPR000719:Protein kinase domain IPR001245:Serine-threonine/tyrosine-protein kinase, catalytic domain IPR002902:Gnk2-homologous domain IPR008271:Serine/threonine-protein kinase, active site IPR011009:Protein kinase-like domain superfamily IPR038408:Gnk2-homologous domain superfamily
3A	AX.94429024	TraesCS3A02G073700	TraesCS3A02G073700.1		IPR001611:Leucine-rich repeat IPR002182:NB-ARC IPR027417:P-loop containing nucleoside triphosphate hydrolase IPR032675:Leucine-rich repeat domain superfamily IPR038005:Virus X resistance protein-like, coiled-coil domain IPR041118:Rx, N-terminal
		TraesCS3A02G073800	TraesCS3A02G073800.1		IPR001611:Leucine-rich repeat IPR002182:NB-ARC IPR027417:P-loop containing nucleoside triphosphate hydrolase IPR032675:Leucine-rich repeat domain superfamily IPR038005:Virus X resistance protein-like, coiled-coil domain IPR041118:Rx, N-terminal
		TraesCS3A02G073900	TraesCS3A02G073900.1		IPR000719:Protein kinase domain IPR008271:Serine/threonine-protein kinase, active site IPR011009:Protein kinase-like domain superfamily
		TraesCS3A02G074300	TraesCS3A02G074300.1		IPR001128:Cytochrome P450 IPR002401:Cytochrome P450, E-class, group I IPR017972:Cytochrome P450, conserved site IPR036396:Cytochrome P450 superfamily
		TraesCS3A02G074400	TraesCS3A02G074400.1		IPR001128:Cytochrome P450 IPR036396:Cytochrome P450 superfamily
		TraesCS3A02G074400	TraesCS3A02G074400.2		IPR001128:Cytochrome P450 IPR002401:Cytochrome P450, E-class, group I IPR017972:Cytochrome P450, conserved site IPR036396:Cytochrome P450 superfamily
		TraesCS3A02G074500	TraesCS3A02G074500.1		IPR001128:Cytochrome P450 IPR002401:Cytochrome P450, E-class, group I IPR017972:Cytochrome P450, conserved site IPR036396:Cytochrome P450 superfamily
		TraesCS3A02G075300	TraesCS3A02G075300.1		IPR036047:F-box-like domain superfamily
		TraesCS3A02G076000	TraesCS3A02G076000.1		IPR002182:NB-ARC IPR027417:P-loop containing nucleoside triphosphate hydrolase IPR038005:Virus X resistance protein-like, coiled-coil domain IPR041118:Rx, N-terminal
		TraesCS3A02G077300	TraesCS3A02G077300.1		IPR001128:Cytochrome P450 IPR002401:Cytochrome P450, E-class, group I IPR017972:Cytochrome P450, conserved site IPR036396:Cytochrome P450 superfamily
3B	AX.94526408	TraesCS3B02G133000	TraesCS3B02G133000.1		IPR001810:F-box domain IPR005174:Domain unknown function DUF295 IPR036047:F-box-like domain superfamily
		TraesCS3B02G133600	TraesCS3B02G133600.1	Uncharacterized protein	IPR001128:Cytochrome P450 IPR002401:Cytochrome P450, E-class, group I IPR017972:Cytochrome P450, conserved site IPR036396:Cytochrome P450 superfamily
		TraesCS3B02G133700	TraesCS3B02G133700.1	Uncharacterized protein	IPR001128:Cytochrome P450 IPR002401:Cytochrome P450, E-class, group I IPR017972:Cytochrome P450, conserved site IPR036396:Cytochrome P450 superfamily
		TraesCS3B02G134100	TraesCS3B02G134100.1		IPR001128:Cytochrome P450 IPR002401:Cytochrome P450, E-class, group I IPR017972:Cytochrome P450, conserved site IPR036396:Cytochrome P450 superfamily
		TraesCS3B02G135000	TraesCS3B02G135000.1	Uncharacterized protein	IPR011009:Protein kinase-like domain superfamily
		TraesCS3B02G135300	TraesCS3B02G135300.1		IPR003657:WRKY domain IPR036576:WRKY domain superfamily
		TraesCS3B02G136100	TraesCS3B02G136100.1		IPR002182:NB-ARC IPR027417:P-loop containing nucleoside triphosphate hydrolase IPR032675:Leucine-rich repeat domain superfamily IPR038005:Virus X resistance protein-like, coiled-coil domain IPR041118:Rx, N-terminal
		TraesCS3B02G136200	TraesCS3B02G136200.1		IPR001128:Cytochrome P450 IPR002401:Cytochrome P450, E-class, group I IPR017972:Cytochrome P450, conserved site IPR036396:Cytochrome P450 superfamily
		TraesCS3B02G136800	TraesCS3B02G136800.1	Uncharacterized protein	IPR003439:ABC transporter-like IPR003593:AAA+ ATPase domain IPR011527:ABC transporter type 1, transmembrane domain IPR017871:ABC transporter, conserved site IPR027417:P-loop containing nucleoside triphosphate hydrolase IPR036640:ABC transporter type 1, transmembrane domain superfamily IPR039421:Type I protein exporter

	AX.94948233	TraesCS3D02G365400	TraesCS3D02G365400.1	IPR001810:F-box domain IPR032675:Leucine-rich repeat domain superfamily IPR036047:F-box-like domain superfamily
		TraesCS3D02G366300	TraesCS3D02G366300.1	IPR001611:Leucine-rich repeat IPR006553:Leucine-rich repeat, cysteine-containing subtype IPR032675:Leucine-rich repeat domain superfamily
		TraesCS3D02G366300	TraesCS3D02G366300.2	IPR001611:Leucine-rich repeat IPR006553:Leucine-rich repeat, cysteine-containing subtype IPR032675:Leucine-rich repeat domain superfamily
		TraesCS3D02G366300	TraesCS3D02G366300.3	IPR001611:Leucine-rich repeat IPR006553:Leucine-rich repeat, cysteine-containing subtype IPR032675:Leucine-rich repeat domain superfamily
3D		TraesCS3D02G367500	TraesCS3D02G367500.1	IPR001128:Cytochrome P450 IPR002397:Cytochrome P450, B-class IPR017972:Cytochrome P450, conserved site IPR036396:Cytochrome P450 superfamily
		TraesCS3D02G368300	TraesCS3D02G368300.1	IPR001810:F-box domain IPR036047:F-box-like domain superfamily
		TraesCS3D02G369100	TraesCS3D02G369100.1	IPR000719:Protein kinase domain IPR008271:Serine/threonine-protein kinase, active site IPR011009:Protein kinase-like domain superfamily IPR017441:Protein kinase, ATP binding site
		TraesCS3D02G369100	TraesCS3D02G369100.2	IPR011009:Protein kinase-like domain superfamily
		TraesCS4A02G361100	TraesCS4A02G361100.1	IPR000719:Protein kinase domain IPR008271:Serine/threonine-protein kinase, active site IPR011009:Protein kinase-like domain superfamily IPR017441:Protein kinase, ATP binding site IPR039192:Glycogen synthase kinase 3, catalytic domain
		TraesCS4A02G366300	TraesCS4A02G366300.1	IPR001810:F-box domain IPR036047:F-box-like domain superfamily
	AX.94428968	TraesCS4A02G473900	TraesCS4A02G473900.1	IPR001810:F-box domain IPR036047:F-box-like domain superfamily
		TraesCS4A02G474400	TraesCS4A02G474400.1	IPR001810:F-box domain IPR036047:F-box-like domain superfamily
		TraesCS4A02G474600	TraesCS4A02G474600.1	IPR001810:F-box domain IPR036047:F-box-like domain superfamily
		TraesCS4A02G474700	TraesCS4A02G474700.1	IPR032675:Leucine-rich repeat domain superfamily
		TraesCS4A02G474800	TraesCS4A02G474800.1	IPR000719:Protein kinase domain IPR001611:Leucine-rich repeat IPR003591:Leucine-rich repeat, typical subtype IPR008266:Tyrosine-protein kinase, active site IPR011009:Protein kinase-like domain superfamily IPR013210:Leucine-rich repeat-containing N-terminal, plant-type IPR017441:Protein kinase, ATP binding site IPR032675:Leucine-rich repeat domain superfamily
		TraesCS4A02G474800	TraesCS4A02G474800.2	IPR000719:Protein kinase domain IPR001611:Leucine-rich repeat IPR003591:Leucine-rich repeat, typical subtype IPR008266:Tyrosine-protein kinase, active site IPR011009:Protein kinase-like domain superfamily IPR013210:Leucine-rich repeat-containing N-terminal, plant-type IPR017441:Protein kinase, ATP binding site IPR032675:Leucine-rich repeat domain superfamily
		TraesCS4A02G474900	TraesCS4A02G474900.1	IPR002182:NB-ARC IPR027417:P-loop containing nucleoside triphosphate hydrolase IPR032675:Leucine-rich repeat domain superfamily IPR038005:Virus X resistance protein-like, coiled-coil domain IPR041118:Rx, N-terminal
		TraesCS4A02G474900	TraesCS4A02G474900.2	IPR002182:NB-ARC IPR027417:P-loop containing nucleoside triphosphate hydrolase IPR032675:Leucine-rich repeat domain superfamily IPR038005:Virus X resistance protein-like, coiled-coil domain IPR041118:Rx, N-terminal
		TraesCS4A02G475400	TraesCS4A02G475400.1	IPR002182:NB-ARC IPR027417:P-loop containing nucleoside triphosphate hydrolase IPR032675:Leucine-rich repeat domain superfamily IPR041118:Rx, N-terminal
		TraesCS4A02G475700	TraesCS4A02G475700.1	IPR032675:Leucine-rich repeat domain superfamily
		TraesCS4A02G475800	TraesCS4A02G475800.1	IPR000719:Protein kinase domain IPR001611:Leucine-rich repeat IPR008271:Serine/threonine-protein kinase, active site IPR011009:Protein kinase-like domain superfamily IPR017441:Protein kinase, ATP binding site IPR032675:Leucine-rich repeat domain superfamily
		TraesCS4A02G475900	TraesCS4A02G475900.1	IPR002182:NB-ARC IPR027417:P-loop containing nucleoside triphosphate hydrolase IPR032675:Leucine-rich repeat domain superfamily

	TraesCS4A02G475900	TraesCS4A02G475900.3	IPR027417:P-loop containing nucleoside triphosphate hydrolase IPR032675:Leucine-rich repeat domain superfamily
	TraesCS4A02G475900	TraesCS4A02G475900.2	IPR002182:NB-ARC IPR027417:P-loop containing nucleoside triphosphate hydrolase IPR032675:Leucine-rich repeat domain superfamily
	TraesCS4A02G476100	TraesCS4A02G476100.1	IPR002182:NB-ARC IPR027417:P-loop containing nucleoside triphosphate hydrolase IPR032675:Leucine-rich repeat domain superfamily
	TraesCS4A02G476300	TraesCS4A02G476300.1	IPR032675:Leucine-rich repeat domain superfamily
	TraesCS4A02G476200	TraesCS4A02G476200.1	IPR002182:NB-ARC IPR027417:P-loop containing nucleoside triphosphate hydrolase IPR032675:Leucine-rich repeat domain superfamily IPR041118:Rx, N-terminal
	TraesCS4A02G476400	TraesCS4A02G476400.1	IPR002182:NB-ARC IPR027417:P-loop containing nucleoside triphosphate hydrolase IPR032675:Leucine-rich repeat domain superfamily
	TraesCS4A02G476600	TraesCS4A02G476600.2	IPR002182:NB-ARC IPR027417:P-loop containing nucleoside triphosphate hydrolase IPR032675:Leucine-rich repeat domain superfamily
	TraesCS4A02G476600	TraesCS4A02G476600.1	IPR002182:NB-ARC IPR027417:P-loop containing nucleoside triphosphate hydrolase IPR032675:Leucine-rich repeat domain superfamily
	TraesCS4A02G476700	TraesCS4A02G476700.1	IPR002182:NB-ARC IPR027417:P-loop containing nucleoside triphosphate hydrolase IPR032675:Leucine-rich repeat domain superfamily
	TraesCS4A02G476800	TraesCS4A02G476800.1	IPR001128:Cytochrome P450 IPR002401:Cytochrome P450, E-class, group I IPR017972:Cytochrome P450, conserved site IPR036396:Cytochrome P450 superfamily
	TraesCS4A02G476900	TraesCS4A02G476900.1	IPR001128:Cytochrome P450 IPR002401:Cytochrome P450, E-class, group I IPR017972:Cytochrome P450, conserved site IPR036396:Cytochrome P450 superfamily
	TraesCS4A02G477300	TraesCS4A02G477300.1	IPR002182:NB-ARC IPR027417:P-loop containing nucleoside triphosphate hydrolase IPR041118:Rx, N-terminal
	TraesCS4A02G477400	TraesCS4A02G477400.1	IPR032675:Leucine-rich repeat domain superfamily
	TraesCS4A02G477600	TraesCS4A02G477600.1	IPR032675:Leucine-rich repeat domain superfamily
	TraesCS4A02G477700	TraesCS4A02G477700.1	IPR002182:NB-ARC IPR027417:P-loop containing nucleoside triphosphate hydrolase IPR041118:Rx, N-terminal
	TraesCS4A02G477800	TraesCS4A02G477800.1	IPR002182:NB-ARC IPR027417:P-loop containing nucleoside triphosphate hydrolase IPR032675:Leucine-rich repeat domain superfamily
	TraesCS4A02G478000	TraesCS4A02G478000.1	IPR032675:Leucine-rich repeat domain superfamily
	TraesCS4A02G478100	TraesCS4A02G478100.1	IPR002182:NB-ARC IPR027417:P-loop containing nucleoside triphosphate hydrolase IPR041118:Rx, N-terminal
	TraesCS4A02G478200	TraesCS4A02G478200.1	IPR002182:NB-ARC IPR027417:P-loop containing nucleoside triphosphate hydrolase IPR032675:Leucine-rich repeat domain superfamily
	TraesCS4A02G478400	TraesCS4A02G478400.1	IPR002182:NB-ARC IPR027417:P-loop containing nucleoside triphosphate hydrolase IPR032675:Leucine-rich repeat domain superfamily
	TraesCS4A02G478600	TraesCS4A02G478600.1	IPR002182:NB-ARC IPR027417:P-loop containing nucleoside triphosphate hydrolase IPR032675:Leucine-rich repeat domain superfamily IPR041118:Rx, N-terminal
4A	TraesCS4A02G478700	TraesCS4A02G478700.1	IPR000719:Protein kinase domain IPR001245:Serine-threonine/tyrosine-protein kinase, catalytic domain IPR001611:Leucine-rich repeat IPR003591:Leucine-rich repeat, typical subtype IPR008271:Serine/threonine-protein kinase, active site IPR011009:Protein kinase-like domain superfamily IPR013210:Leucine-rich repeat-containing N-terminal, plant-type IPR017441:Protein kinase, ATP binding site IPR032675:Leucine-rich repeat domain superfamily

TraesCS4A02G478800	TraesCS4A02G478800.1	IPR002182:NB-ARC IPR027417:P-loop containing nucleoside triphosphate hydrolase IPR032675:Leucine-rich repeat domain superfamily
TraesCS4A02G478900	TraesCS4A02G478900.1	IPR002182:NB-ARC IPR027417:P-loop containing nucleoside triphosphate hydrolase IPR032675:Leucine-rich repeat domain superfamily
TraesCS4A02G479100	TraesCS4A02G479100.1	IPR002182:NB-ARC IPR027417:P-loop containing nucleoside triphosphate hydrolase IPR041118:Rx, N-terminal
TraesCS4A02G479200	TraesCS4A02G479200.1	IPR001611:Leucine-rich repeat IPR013210:Leucine-rich repeat-containing N-terminal, plant-type IPR032675:Leucine-rich repeat domain superfamily
TraesCS4A02G479300	TraesCS4A02G479300.1	IPR000719:Protein kinase domain IPR001245:Serine-threonine/tyrosine-protein kinase, catalytic domain IPR008271:Serine/threonine-protein kinase, active site IPR011009:Protein kinase-like domain superfamily IPR017441:Protein kinase, ATP binding site
TraesCS4A02G479400	TraesCS4A02G479400.1	IPR002182:NB-ARC IPR027417:P-loop containing nucleoside triphosphate hydrolase IPR032675:Leucine-rich repeat domain superfamily IPR041118:Rx, N-terminal
TraesCS4A02G479700	TraesCS4A02G479700.1	IPR002182:NB-ARC IPR027417:P-loop containing nucleoside triphosphate hydrolase IPR032675:Leucine-rich repeat domain superfamily IPR041118:Rx, N-terminal
TraesCS4A02G479900	TraesCS4A02G479900.1	IPR002182:NB-ARC IPR027417:P-loop containing nucleoside triphosphate hydrolase
TraesCS4A02G480000	TraesCS4A02G480000.1	IPR032675:Leucine-rich repeat domain superfamily
TraesCS4A02G480100	TraesCS4A02G480100.1	IPR002182:NB-ARC IPR027417:P-loop containing nucleoside triphosphate hydrolase IPR032675:Leucine-rich repeat domain superfamily IPR041118:Rx, N-terminal
TraesCS4A02G480200	TraesCS4A02G480200.1	IPR002182:NB-ARC IPR027417:P-loop containing nucleoside triphosphate hydrolase IPR032675:Leucine-rich repeat domain superfamily
TraesCS4A02G480300	TraesCS4A02G480300.1	IPR032675:Leucine-rich repeat domain superfamily
TraesCS4A02G480400	TraesCS4A02G480400.1	IPR001128:Cytochrome P450 IPR002401:Cytochrome P450, E-class, group I IPR017972:Cytochrome P450, conserved site IPR036396:Cytochrome P450 superfamily
TraesCS4A02G481200	TraesCS4A02G481200.1	IPR000719:Protein kinase domain IPR011009:Protein kinase-like domain superfamily IPR017441:Protein kinase, ATP binding site
TraesCS4A02G481300	TraesCS4A02G481300.4	IPR000719:Protein kinase domain IPR008271:Serine/threonine-protein kinase, active site IPR011009:Protein kinase-like domain superfamily IPR017441:Protein kinase, ATP binding site IPR024171:S-receptor-like serine/threonine-protein kinase
TraesCS4A02G481300	TraesCS4A02G481300.1	IPR000719:Protein kinase domain IPR008271:Serine/threonine-protein kinase, active site IPR011009:Protein kinase-like domain superfamily IPR017441:Protein kinase, ATP binding site IPR024171:S-receptor-like serine/threonine-protein kinase
TraesCS4A02G481300	TraesCS4A02G481300.5	IPR000719:Protein kinase domain IPR011009:Protein kinase-like domain superfamily IPR017441:Protein kinase, ATP binding site
TraesCS4A02G481300	TraesCS4A02G481300.2	IPR000719:Protein kinase domain IPR008271:Serine/threonine-protein kinase, active site IPR011009:Protein kinase-like domain superfamily IPR017441:Protein kinase, ATP binding site IPR024171:S-receptor-like serine/threonine-protein kinase
TraesCS4A02G481300	TraesCS4A02G481300.3	IPR000719:Protein kinase domain IPR008271:Serine/threonine-protein kinase, active site IPR011009:Protein kinase-like domain superfamily IPR017441:Protein kinase, ATP binding site IPR024171:S-receptor-like serine/threonine-protein kinase
TraesCS4A02G481600	TraesCS4A02G481600.1	IPR000719:Protein kinase domain IPR008271:Serine/threonine-protein kinase, active site IPR011009:Protein kinase-like domain superfamily IPR017441:Protein kinase, ATP binding site
TraesCS4A02G482500	TraesCS4A02G482500.1	IPR000719:Protein kinase domain IPR011009:Protein kinase-like domain superfamily
TraesCS4A02G482600	TraesCS4A02G482600.1	IPR000719:Protein kinase domain IPR011009:Protein kinase-like domain superfamily

		TraesCS4A02G482700	TraesCS4A02G482700.1		IPR000719:Protein kinase domain IPR011009:Protein kinase-like domain superfamily
		TraesCS4A02G482800	TraesCS4A02G482800.1		IPR001611:Leucine-rich repeat IPR013210:Leucine-rich repeat-containing N-terminal, plant-type IPR032675:Leucine-rich repeat domain superfamily
		TraesCS4A02G482900	TraesCS4A02G482900.1		IPR000719:Protein kinase domain IPR011009:Protein kinase-like domain superfamily
		TraesCS4A02G483000	TraesCS4A02G483000.1		IPR000719:Protein kinase domain IPR011009:Protein kinase-like domain superfamily
		TraesCS4A02G483200	TraesCS4A02G483200.1		IPR001611:Leucine-rich repeat IPR003591:Leucine-rich repeat, typical subtype IPR013210:Leucine-rich repeat-containing N-terminal, plant-type IPR032675:Leucine-rich repeat domain superfamily
		TraesCS4A02G483300	TraesCS4A02G483300.1		IPR000719:Protein kinase domain IPR011009:Protein kinase-like domain superfamily
		TraesCS4A02G483300	TraesCS4A02G483300.2		IPR000719:Protein kinase domain IPR011009:Protein kinase-like domain superfamily
		TraesCS4A02G483300	TraesCS4A02G483300.3		IPR000719:Protein kinase domain IPR011009:Protein kinase-like domain superfamily
		TraesCS4A02G483400	TraesCS4A02G483400.1		IPR001611:Leucine-rich repeat
		TraesCS4A02G483500	TraesCS4A02G483500.1		IPR001611:Leucine-rich repeat IPR003591:Leucine-rich repeat, typical subtype IPR032675:Leucine-rich repeat domain superfamily
		TraesCS4A02G483600	TraesCS4A02G483600.1	Serine/threonine-protein kinase [Source:UniProtKB/TrEMBL;Acc:W5DMF5]	IPR000719:Protein kinase domain IPR000858:S-locus glycoprotein domain IPR001480:Bulb-type lectin domain IPR003609:PAN/Apple domain IPR008271:Serine/threonine-protein kinase, active site IPR011009:Protein kinase-like domain superfamily IPR017441:Protein kinase, ATP binding site IPR024171:S-receptor-like serine/threonine-protein kinase IPR036426:Bulb-type lectin domain superfamily
		TraesCS4A02G483700	TraesCS4A02G483700.1		IPR001611:Leucine-rich repeat IPR003591:Leucine-rich repeat, typical subtype IPR032675:Leucine-rich repeat domain superfamily
	AX.94828722	TraesCS4B02G384500	TraesCS4B02G384500.1		IPR036047:F-box-like domain superfamily
		TraesCS4B02G385000	TraesCS4B02G385000.1	Peroxidase [Source:UniProtKB/TrEMBL;Acc:A0A1D5XGR8]	IPR000823:Plant peroxidase IPR002016:Haem peroxidase IPR010255:Haem peroxidase superfamily IPR019793:Peroxidases heam-ligand binding site IPR019794:Peroxidase, active site IPR033905:Secretory peroxidase
		TraesCS4B02G387600	TraesCS4B02G387600.1		IPR000719:Protein kinase domain IPR008271:Serine/threonine-protein kinase, active site IPR011009:Protein kinase-like domain superfamily IPR017441:Protein kinase, ATP binding site
		TraesCS4B02G388000	TraesCS4B02G388000.1		IPR036047:F-box-like domain superfamily
		TraesCS4B02G388500	TraesCS4B02G388500.1		IPR001128:Cytochrome P450 IPR002401:Cytochrome P450, E-class, group I IPR017972:Cytochrome P450, conserved site IPR036396:Cytochrome P450 superfamily
		TraesCS4B02G388600	TraesCS4B02G388600.1		IPR001128:Cytochrome P450 IPR002401:Cytochrome P450, E-class, group I IPR017972:Cytochrome P450, conserved site IPR036396:Cytochrome P450 superfamily
4B		TraesCS4B02G388800	TraesCS4B02G388800.1	Protein DETOXIFICATION [Source:UniProtKB/TrEMBL;Acc:A0A1D6DH59]	IPR002528:Multi antimicrobial extrusion protein
		TraesCS4B02G389200	TraesCS4B02G389200.1		IPR036047:F-box-like domain superfamily
		TraesCS4B02G389300	TraesCS4B02G389300.1		IPR001128:Cytochrome P450 IPR002403:Cytochrome P450, E-class, group IV IPR017972:Cytochrome P450, conserved site IPR036396:Cytochrome P450 superfamily
		TraesCS4B02G389400	TraesCS4B02G389400.1		IPR001128:Cytochrome P450 IPR002401:Cytochrome P450, E-class, group I IPR017972:Cytochrome P450, conserved site IPR036396:Cytochrome P450 superfamily
		TraesCS4B02G389600	TraesCS4B02G389600.1		IPR036047:F-box-like domain superfamily

		TraesCS4B02G389700	TraesCS4B02G389700.1		IPR000719:Protein kinase domain IPR001245:Serine-threonine/tyrosine-protein kinase, catalytic domain IPR001611:Leucine-rich repeat IPR008271:Serine/threonine-protein kinase, active site IPR011009:Protein kinase-like domain superfamily IPR017441:Protein kinase, ATP binding site IPR024788:Malectin-like domain IPR025875:Leucine rich repeat 4 IPR032675:Leucine-rich repeat domain superfamily
		TraesCS4B02G391600	TraesCS4B02G391600.1		IPR036047:F-box-like domain superfamily
		TraesCS4B02G392900	TraesCS4B02G392900.1		IPR001810:F-box domain IPR036047:F-box-like domain superfamily
	AX.94792356	TraesCS4D02G074100	TraesCS4D02G074100.1		IPR000719:Protein kinase domain IPR001611:Leucine-rich repeat IPR003591:Leucine-rich repeat, typical subtype IPR011009:Protein kinase-like domain superfamily IPR032675:Leucine-rich repeat domain superfamily
4D		TraesCS4D02G078100	TraesCS4D02G078100.2	SNF1-type serine-threonine protein kinase [Source:UniProtKB/TrEMBL;Acc:W5ES46] SNF1-type serine-threonine protein kinase	IPR000719:Protein kinase domain IPR008271:Serine/threonine-protein kinase, active site IPR011009:Protein kinase-like domain superfamily IPR017441:Protein kinase, ATP binding site
		TraesCS4D02G078100	TraesCS4D02G078100.1	SNF1-type serine-threonine protein kinase [Source:UniProtKB/TrEMBL;Acc:W5ES46]	IPR000719:Protein kinase domain IPR008271:Serine/threonine-protein kinase, active site IPR011009:Protein kinase-like domain superfamily
		TraesCS4D02G079000	TraesCS4D02G079000.1		IPR001810:F-box domain IPR036047:F-box-like domain superfamily
	AX.94699167	TraesCS5A02G387000	TraesCS5A02G387000.1		IPR000719:Protein kinase domain IPR008271:Serine/threonine-protein kinase, active site IPR011009:Protein kinase-like domain superfamily IPR017441:Protein kinase, ATP binding site
		TraesCS5A02G387100	TraesCS5A02G387100.1		IPR000719:Protein kinase domain IPR008271:Serine/threonine-protein kinase, active site IPR011009:Protein kinase-like domain superfamily IPR017441:Protein kinase, ATP binding site
		TraesCS5A02G388600	TraesCS5A02G388600.1		IPR001810:F-box domain IPR036047:F-box-like domain superfamily
		TraesCS5A02G388800	TraesCS5A02G388800.1		IPR000719:Protein kinase domain IPR001611:Leucine-rich repeat IPR003591:Leucine-rich repeat, typical subtype IPR008271:Serine/threonine-protein kinase, active site IPR011009:Protein kinase-like domain superfamily IPR013210:Leucine-rich repeat-containing N-terminal, plant-type IPR017441:Protein kinase, ATP binding site IPR032675:Leucine-rich repeat domain superfamily
		TraesCS5A02G389200	TraesCS5A02G389200.1	Tousled-like protein kinase [Source:UniProtKB/TrEMBL;Acc:Q5I6Y0] Tousled-like protein kinase	IPR000719:Protein kinase domain IPR008271:Serine/threonine-protein kinase, active site IPR011009:Protein kinase-like domain superfamily IPR017441:Protein kinase, ATP binding site
		TraesCS5A02G389200	TraesCS5A02G389200.2	Tousled-like protein kinase [Source:UniProtKB/TrEMBL;Acc:Q5I6Y0]	IPR000719:Protein kinase domain IPR008271:Serine/threonine-protein kinase, active site IPR011009:Protein kinase-like domain superfamily IPR017441:Protein kinase, ATP binding site
5A		TraesCS5A02G390900	TraesCS5A02G390900.1	Protein TORNADO 1 [Source:Projected from Arabidopsis thaliana (AT5G55540) UniProtKB/Swiss-Prot;Acc:Q9FJ57]	IPR001611:Leucine-rich repeat IPR032675:Leucine-rich repeat domain superfamily

		TraesCS5A02G391900	TraesCS5A02G391900.1	CYB5 [Source:UniProtKB/TrEMBL;Acc:M9PJX7] CYB5	IPR001199:Cytochrome b5-like heme/steroid binding domain IPR018506:Cytochrome b5, heme-binding site IPR036400:Cytochrome b5-like heme/steroid binding domain superfamily
		TraesCS5A02G392500	TraesCS5A02G392500.1	Protein kinase superfamily protein [Source:Projected from Arabidopsis thaliana (AT1G69220) UniProtKB/TrEMBL;Acc:O24527]	IPR000719:Protein kinase domain IPR011009:Protein kinase-like domain superfamily IPR017441:Protein kinase, ATP binding site
		TraesCS5A02G393000	TraesCS5A02G393000.1		IPR000719:Protein kinase domain IPR001611:Leucine-rich repeat IPR003591:Leucine-rich repeat, typical subtype IPR008271:Serine/threonine-protein kinase, active site IPR011009:Protein kinase-like domain superfamily IPR013210:Leucine-rich repeat-containing N-terminal, plant-type IPR017441:Protein kinase, ATP binding site IPR032675:Leucine-rich repeat domain superfamily
	AX.94406039	TraesCS5B02G003200	TraesCS5B02G003200.1		IPR001611:Leucine-rich repeat IPR032675:Leucine-rich repeat domain superfamily
		TraesCS5B02G003200	TraesCS5B02G003200.2		IPR001611:Leucine-rich repeat IPR032675:Leucine-rich repeat domain superfamily
		TraesCS5B02G004300	TraesCS5B02G004300.1		IPR000719:Protein kinase domain IPR008271:Serine/threonine-protein kinase, active site IPR011009:Protein kinase-like domain superfamily IPR013210:Leucine-rich repeat-containing N-terminal, plant-type IPR017441:Protein kinase, ATP binding site IPR031071:LRR receptor-like serine/threonine-protein kinase FEI1/2 IPR032675:Leucine-rich repeat domain superfamily
		TraesCS5B02G004500	TraesCS5B02G004500.1		IPR001128:Cytochrome P450 IPR002403:Cytochrome P450, E-class, group IV IPR017972:Cytochrome P450, conserved site IPR036396:Cytochrome P450 superfamily
		TraesCS5B02G004600	TraesCS5B02G004600.1		IPR001128:Cytochrome P450 IPR002403:Cytochrome P450, E-class, group IV IPR017972:Cytochrome P450, conserved site IPR036396:Cytochrome P450 superfamily
		TraesCS5B02G004900	TraesCS5B02G004900.1		IPR001128:Cytochrome P450 IPR002403:Cytochrome P450, E-class, group IV IPR017972:Cytochrome P450, conserved site IPR036396:Cytochrome P450 superfamily
		TraesCS5B02G005400	TraesCS5B02G005400.2		IPR000719:Protein kinase domain IPR001245:Serine-threonine/tyrosine-protein kinase, catalytic domain IPR008271:Serine/threonine-protein kinase, active site IPR011009:Protein kinase-like domain superfamily IPR017441:Protein kinase, ATP binding site
5B		TraesCS5B02G005400	TraesCS5B02G005400.1		IPR000719:Protein kinase domain IPR001245:Serine-threonine/tyrosine-protein kinase, catalytic domain IPR008271:Serine/threonine-protein kinase, active site IPR011009:Protein kinase-like domain superfamily IPR017441:Protein kinase, ATP binding site
		TraesCS5B02G005400	TraesCS5B02G005400.3		IPR000719:Protein kinase domain IPR001245:Serine-threonine/tyrosine-protein kinase, catalytic domain IPR008271:Serine/threonine-protein kinase, active site IPR011009:Protein kinase-like domain superfamily IPR017441:Protein kinase, ATP binding site
		TraesCS5B02G005700	TraesCS5B02G005700.1		IPR000719:Protein kinase domain IPR002182:NB-ARC IPR008271:Serine/threonine-protein kinase, active site IPR011009:Protein kinase-like domain superfamily IPR017441:Protein kinase, ATP binding site IPR027417:P-loop containing nucleoside triphosphate hydrolase
		TraesCS5B02G007000	TraesCS5B02G007000.2		IPR001128:Cytochrome P450 IPR002401:Cytochrome P450, E-class, group I IPR017972:Cytochrome P450, conserved site IPR036396:Cytochrome P450 superfamily
		TraesCS5B02G007000	TraesCS5B02G007000.1		IPR001128:Cytochrome P450 IPR002401:Cytochrome P450, E-class, group I IPR017972:Cytochrome P450, conserved site IPR036396:Cytochrome P450 superfamily

		TraesCS5B02G007100	TraesCS5B02G007100.1	p450 [Source:UniProtKB/TrEMBL;Acc:Q58A30] P450	IPR001128:Cytochrome P450 IPR002401:Cytochrome P450, E-class, group I IPR017972:Cytochrome P450, conserved site IPR036396:Cytochrome P450 superfamily
		TraesCS5B02G007200	TraesCS5B02G007200.1	p450 [Source:UniProtKB/TrEMBL;Acc:Q58A32] P450	IPR001128:Cytochrome P450 IPR002401:Cytochrome P450, E-class, group I IPR017972:Cytochrome P450, conserved site IPR036396:Cytochrome P450 superfamily
	AX.95010548	TraesCS5B02G056700	TraesCS5B02G056700.1		IPR000719:Protein kinase domain IPR002902:Gnk2-homologous domain IPR008271:Serine/threonine-protein kinase, active site IPR011009:Protein kinase-like domain superfamily IPR017441:Protein kinase, ATP binding site IPR038408:Gnk2-homologous domain superfamily
		TraesCS5B02G056800	TraesCS5B02G056800.1		IPR000719:Protein kinase domain IPR002902:Gnk2-homologous domain IPR008271:Serine/threonine-protein kinase, active site IPR011009:Protein kinase-like domain superfamily IPR017441:Protein kinase, ATP binding site IPR038408:Gnk2-homologous domain superfamily
		TraesCS5B02G056900	TraesCS5B02G056900.1		IPR000719:Protein kinase domain IPR002902:Gnk2-homologous domain IPR008271:Serine/threonine-protein kinase, active site IPR011009:Protein kinase-like domain superfamily IPR017441:Protein kinase, ATP binding site IPR038408:Gnk2-homologous domain superfamily
		TraesCS5B02G057205	TraesCS5B02G057205.1		IPR000719:Protein kinase domain IPR008271:Serine/threonine-protein kinase, active site IPR011009:Protein kinase-like domain superfamily IPR017441:Protein kinase, ATP binding site IPR025287:Wall-associated receptor kinase, galacturonan-binding domain
		TraesCS5B02G057500	TraesCS5B02G057500.1		IPR000719:Protein kinase domain IPR008271:Serine/threonine-protein kinase, active site IPR011009:Protein kinase-like domain superfamily IPR017441:Protein kinase, ATP binding site
		TraesCS5B02G057700	TraesCS5B02G057700.1		IPR000719:Protein kinase domain IPR002902:Gnk2-homologous domain IPR008271:Serine/threonine-protein kinase, active site IPR011009:Protein kinase-like domain superfamily IPR017441:Protein kinase, ATP binding site IPR038408:Gnk2-homologous domain superfamily
		TraesCS5B02G057900	TraesCS5B02G057900.1		IPR006566:FBD domain IPR032675:Leucine-rich repeat domain superfamily IPR036047:F-box-like domain superfamily
5B		TraesCS5B02G058100	TraesCS5B02G058100.1		IPR006566:FBD domain IPR032675:Leucine-rich repeat domain superfamily IPR036047:F-box-like domain superfamily
		TraesCS5B02G058300	TraesCS5B02G058300.1		IPR001810:F-box domain IPR032675:Leucine-rich repeat domain superfamily IPR036047:F-box-like domain superfamily
		TraesCS5B02G058900	TraesCS5B02G058900.1		IPR032675:Leucine-rich repeat domain superfamily
		TraesCS5B02G059000	TraesCS5B02G059000.2		IPR000719:Protein kinase domain IPR001611:Leucine-rich repeat IPR002182:NB-ARC IPR003591:Leucine-rich repeat, typical subtype IPR008271:Serine/threonine-protein kinase, active site IPR011009:Protein kinase-like domain superfamily IPR017441:Protein kinase, ATP binding site IPR027417:P-loop containing nucleoside triphosphate hydrolase IPR032675:Leucine-rich repeat domain superfamily
		TraesCS5B02G059000	TraesCS5B02G059000.1		IPR000719:Protein kinase domain IPR001611:Leucine-rich repeat IPR002182:NB-ARC IPR003591:Leucine-rich repeat, typical subtype IPR008271:Serine/threonine-protein kinase, active site IPR011009:Protein kinase-like domain superfamily IPR017441:Protein kinase, ATP binding site IPR027417:P-loop containing nucleoside triphosphate hydrolase IPR032675:Leucine-rich repeat domain superfamily

5B	#N/A	TraesCS5B02G059300	TraesCS5B02G059300.1	Non-specific serine/threonine protein kinase [Source:UniProtKB/TrEMBL;Acc:A0A341VB71]	IPR000719:Protein kinase domain IPR004041:NAF domain IPR008271:Serine/threonine-protein kinase, active site IPR011009:Protein kinase-like domain superfamily IPR017441:Protein kinase, ATP binding site IPR018451:NAF/FISL domain
		TraesCS5B02G059500	TraesCS5B02G059500.1		IPR001810:F-box domain IPR032675:Leucine-rich repeat domain superfamily IPR036047:F-box-like domain superfamily
		TraesCS5B02G059600	TraesCS5B02G059600.1		IPR032675:Leucine-rich repeat domain superfamily
		TraesCS5B02G367300	TraesCS5B02G367300.1		IPR025287:Wall-associated receptor kinase, galacturonan-binding domain
		TraesCS5B02G367500	TraesCS5B02G367500.1		IPR000719:Protein kinase domain IPR000858:S-locus glycoprotein domain IPR001480:Bulb-type lectin domain IPR011009:Protein kinase-like domain superfamily IPR024171:S-receptor-like serine/threonine-protein kinase IPR036426:Bulb-type lectin domain superfamily
		TraesCS5B02G369200	TraesCS5B02G369200.1		IPR013989:Development/cell death domain
		TraesCS5B02G370400	TraesCS5B02G370400.1	Serine/threonine-protein kinase [Source:UniProtKB/TrEMBL;Acc:W5FIJ2]	IPR000719:Protein kinase domain IPR000858:S-locus glycoprotein domain IPR001480:Bulb-type lectin domain IPR003609:PAN/Apple domain IPR008271:Serine/threonine-protein kinase, active site IPR011009:Protein kinase-like domain superfamily IPR017441:Protein kinase, ATP binding site IPR024171:S-receptor-like serine/threonine-protein kinase IPR036426:Bulb-type lectin domain superfamily
		TraesCS5B02G371700	TraesCS5B02G371700.1		IPR001611:Leucine-rich repeat IPR011049:Serralysin-like metalloprotease, C-terminal IPR013210:Leucine-rich repeat-containing N-terminal, plant-type IPR032675:Leucine-rich repeat domain superfamily
		TraesCS5B02G371800	TraesCS5B02G371800.1		IPR000719:Protein kinase domain IPR008271:Serine/threonine-protein kinase, active site IPR011009:Protein kinase-like domain superfamily
		TraesCS5B02G371800	TraesCS5B02G371800.2		IPR000719:Protein kinase domain IPR008271:Serine/threonine-protein kinase, active site IPR011009:Protein kinase-like domain superfamily
	AX.94457982	TraesCS5B02G406400	TraesCS5B02G406400.1	Sucrose non-fermenting-1-related protein kinase 2.8 [Source:UniProtKB/TrEMBL;Acc:W5F978] Sucrose non-fermenting-1-related protein kinase 2.8	IPR000719:Protein kinase domain IPR008271:Serine/threonine-protein kinase, active site IPR011009:Protein kinase-like domain superfamily IPR017441:Protein kinase, ATP binding site
		TraesCS5B02G406600	TraesCS5B02G406600.1	Protein kinase domain-containing protein	IPR000719:Protein kinase domain IPR008271:Serine/threonine-protein kinase, active site IPR011009:Protein kinase-like domain superfamily IPR017441:Protein kinase, ATP binding site IPR030616:Aurora kinase
5B		TraesCS5B02G408000	TraesCS5B02G408000.1	Allene oxide synthase, chloroplastic [Source:Projected from Arabidopsis thaliana (AT5G42650) UniProtKB/Swiss-Prot;Acc:Q96242]	IPR001128:Cytochrome P450 IPR002403:Cytochrome P450, E-class, group IV IPR036396:Cytochrome P450 superfamily
		TraesCS5B02G408500	TraesCS5B02G408500.1	Protein DETOXIFICATION [Source:UniProtKB/TrEMBL;Acc:A0A341VJT2]	IPR002528:Multi antimicrobial extrusion protein

		TraesCS5B02G410900	TraesCS5B02G410900.1	IPR000719:Protein kinase domain IPR001220:Legume lectin domain IPR008271:Serine/threonine-protein kinase, active site IPR011009:Protein kinase-like domain superfamily IPR013320:Concanavalin A-like lectin/glucanase domain superfamily IPR017441:Protein kinase, ATP binding site
		TraesCS5B02G411200	TraesCS5B02G411200.1	IPR001128:Cytochrome P450 IPR002401:Cytochrome P450, E-class, group I IPR017972:Cytochrome P450, conserved site IPR036396:Cytochrome P450 superfamily
	AX.95126753	TraesCS5D02G003500	TraesCS5D02G003500.1	IPR000008:C2 domain IPR000719:Protein kinase domain IPR008271:Serine/threonine-protein kinase, active site IPR011009:Protein kinase-like domain superfamily IPR017441:Protein kinase, ATP binding site IPR035892:C2 domain superfamily
		TraesCS5D02G004900	TraesCS5D02G004900.1	IPR001128:Cytochrome P450 IPR002401:Cytochrome P450, E-class, group I IPR017972:Cytochrome P450, conserved site IPR036396:Cytochrome P450 superfamily
		TraesCS5D02G005300	TraesCS5D02G005300.1	IPR000719:Protein kinase domain IPR001611:Leucine-rich repeat IPR002182:NB-ARC IPR008271:Serine/threonine-protein kinase, active site IPR011009:Protein kinase-like domain superfamily IPR017441:Protein kinase, ATP binding site IPR027417:P-loop containing nucleoside triphosphate hydrolase IPR032675:Leucine-rich repeat domain superfamily
		TraesCS5D02G005400	TraesCS5D02G005400.1	IPR000719:Protein kinase domain IPR002182:NB-ARC IPR003591:Leucine-rich repeat, typical subtype IPR008271:Serine/threonine-protein kinase, active site IPR011009:Protein kinase-like domain superfamily IPR017441:Protein kinase, ATP binding site IPR027417:P-loop containing nucleoside triphosphate hydrolase IPR032675:Leucine-rich repeat domain superfamily
		TraesCS5D02G005416	TraesCS5D02G005416.1	IPR001611:Leucine-rich repeat IPR032675:Leucine-rich repeat domain superfamily
		TraesCS5D02G005600	TraesCS5D02G005600.1	IPR001611:Leucine-rich repeat IPR002182:NB-ARC IPR025875:Leucine rich repeat 4 IPR027417:P-loop containing nucleoside triphosphate hydrolase IPR032675:Leucine-rich repeat domain superfamily
		TraesCS5D02G006200	TraesCS5D02G006200.1	IPR001128:Cytochrome P450 IPR002403:Cytochrome P450, E-class, group IV IPR017972:Cytochrome P450, conserved site IPR036396:Cytochrome P450 superfamily
		TraesCS5D02G006400	TraesCS5D02G006400.1	IPR001128:Cytochrome P450 IPR002403:Cytochrome P450, E-class, group IV IPR036396:Cytochrome P450 superfamily
		TraesCS5D02G006500	TraesCS5D02G006500.1	IPR001128:Cytochrome P450 IPR002403:Cytochrome P450, E-class, group IV IPR036396:Cytochrome P450 superfamily
		TraesCS5D02G006800	TraesCS5D02G006800.1	IPR001128:Cytochrome P450 IPR002401:Cytochrome P450, E-class, group I IPR017972:Cytochrome P450, conserved site IPR036396:Cytochrome P450 superfamily
5D		TraesCS5D02G006900	TraesCS5D02G006900.1	IPR001810:F-box domain IPR032675:Leucine-rich repeat domain superfamily IPR036047:F-box-like domain superfamily
		TraesCS5D02G007100	TraesCS5D02G007100.1	IPR000719:Protein kinase domain IPR008271:Serine/threonine-protein kinase, active site IPR011009:Protein kinase-like domain superfamily IPR017441:Protein kinase, ATP binding site IPR024171:S-receptor-like serine/threonine-protein kinase
		TraesCS5D02G007100	TraesCS5D02G007100.3	IPR000719:Protein kinase domain IPR008271:Serine/threonine-protein kinase, active site IPR011009:Protein kinase-like domain superfamily IPR017441:Protein kinase, ATP binding site
		TraesCS5D02G007100	TraesCS5D02G007100.2	IPR000719:Protein kinase domain IPR008271:Serine/threonine-protein kinase, active site IPR011009:Protein kinase-like domain superfamily IPR017441:Protein kinase, ATP binding site

		TraesCS5D02G007200	TraesCS5D02G007200.1		IPR000719:Protein kinase domain IPR008271:Serine/threonine-protein kinase, active site IPR011009:Protein kinase-like domain superfamily IPR013210:Leucine-rich repeat-containing N-terminal, plant-type IPR017441:Protein kinase, ATP binding site IPR031071:LRR receptor-like serine/threonine-protein kinase FEI1/2 IPR032675:Leucine-rich repeat domain superfamily
		TraesCS5D02G009100	TraesCS5D02G009100.1		IPR005797:Cytochrome b/b6, N-terminal IPR016174:Di-haem cytochrome, transmembrane IPR027387:Cytochrome b/b6-like domain superfamily
		TraesCS5D02G011700	TraesCS5D02G011700.1		IPR001810:F-box domain IPR032675:Leucine-rich repeat domain superfamily IPR036047:F-box-like domain superfamily
		TraesCS5D02G012000	TraesCS5D02G012000.1		IPR001128:Cytochrome P450 IPR002403:Cytochrome P450, E-class, group IV IPR036396:Cytochrome P450 superfamily
		TraesCS5D02G012100	TraesCS5D02G012100.1		IPR001128:Cytochrome P450 IPR002403:Cytochrome P450, E-class, group IV IPR036396:Cytochrome P450 superfamily
		TraesCS5D02G012200	TraesCS5D02G012200.1		IPR036396:Cytochrome P450 superfamily
		TraesCS5D02G012300	TraesCS5D02G012300.1		IPR001128:Cytochrome P450 IPR002403:Cytochrome P450, E-class, group IV IPR036396:Cytochrome P450 superfamily
		TraesCS5D02G012300	TraesCS5D02G012300.2		IPR001128:Cytochrome P450 IPR002403:Cytochrome P450, E-class, group IV IPR036396:Cytochrome P450 superfamily
	AX.95086097	TraesCS6A02G077200	TraesCS6A02G077200.1		IPR001810:F-box domain IPR036047:F-box-like domain superfamily
		TraesCS6A02G077600	TraesCS6A02G077600.1		IPR001810:F-box domain IPR032675:Leucine-rich repeat domain superfamily IPR036047:F-box-like domain superfamily
		TraesCS6A02G077700	TraesCS6A02G077700.1		IPR001810:F-box domain IPR006566:FBD domain IPR032675:Leucine-rich repeat domain superfamily IPR036047:F-box-like domain superfamily
		TraesCS6A02G077900	TraesCS6A02G077900.1		IPR006566:FBD domain IPR032675:Leucine-rich repeat domain superfamily
		TraesCS6A02G078000	TraesCS6A02G078000.1		IPR001810:F-box domain IPR006566:FBD domain IPR032675:Leucine-rich repeat domain superfamily IPR036047:F-box-like domain superfamily
		TraesCS6A02G078100	TraesCS6A02G078100.1		IPR006566:FBD domain IPR032675:Leucine-rich repeat domain superfamily
		TraesCS6A02G078200	TraesCS6A02G078200.1		IPR000719:Protein kinase domain IPR008271:Serine/threonine-protein kinase, active site IPR011009:Protein kinase-like domain superfamily IPR017441:Protein kinase, ATP binding site
		TraesCS6A02G078500	TraesCS6A02G078500.1		IPR032675:Leucine-rich repeat domain superfamily IPR036047:F-box-like domain superfamily
		TraesCS6A02G078700	TraesCS6A02G078700.1		IPR000719:Protein kinase domain IPR000858:S-locus glycoprotein domain IPR001245:Serine-threonine/tyrosine-protein kinase, catalytic domain IPR001480:Bulb-type lectin domain IPR003609:PAN/Apple domain IPR011009:Protein kinase-like domain superfamily IPR017441:Protein kinase, ATP binding site IPR024171:S-receptor-like serine/threonine-protein kinase IPR036426:Bulb-type lectin domain superfamily
6A		TraesCS6A02G080100	TraesCS6A02G080100.1		IPR002182:NB-ARC IPR027417:P-loop containing nucleoside triphosphate hydrolase IPR032675:Leucine-rich repeat domain superfamily
		TraesCS6A02G080500	TraesCS6A02G080500.1	WRKY DNA-binding protein 55 [Source:Projected from Arabidopsis thaliana (AT2G40740) TAIR;Acc:AT2G40740]	IPR003657:WRKY domain IPR036576:WRKY domain superfamily
		TraesCS6A02G081800	TraesCS6A02G081800.1	Uncharacterized protein	IPR001128:Cytochrome P450 IPR002401:Cytochrome P450, E-class, group I IPR017972:Cytochrome P450, conserved site IPR036396:Cytochrome P450 superfamily

		TraesCS6A02G081900	TraesCS6A02G081900.1	Uncharacterized protein	IPR001128:Cytochrome P450 IPR002401:Cytochrome P450, E-class, group I IPR017972:Cytochrome P450, conserved site IPR036396:Cytochrome P450 superfamily
		TraesCS6A02G082000	TraesCS6A02G082000.1		IPR001128:Cytochrome P450 IPR002401:Cytochrome P450, E-class, group I IPR036396:Cytochrome P450 superfamily
		TraesCS6A02G082700	TraesCS6A02G082700.1		IPR001810:F-box domain IPR032675:Leucine-rich repeat domain superfamily IPR036047:F-box-like domain superfamily
		TraesCS6A02G083500	TraesCS6A02G083500.1		IPR001810:F-box domain IPR036047:F-box-like domain superfamily
		TraesCS6A02G083600	TraesCS6A02G083600.1		IPR001810:F-box domain IPR036047:F-box-like domain superfamily
		TraesCS6A02G083800	TraesCS6A02G083800.1		IPR001128:Cytochrome P450 IPR002401:Cytochrome P450, E-class, group I IPR017972:Cytochrome P450, conserved site IPR036396:Cytochrome P450 superfamily
	AX.95019005	TraesCS6A02G367600	TraesCS6A02G367600.1		IPR001810:F-box domain IPR036047:F-box-like domain superfamily
		TraesCS6A02G369100	TraesCS6A02G369100.2		IPR032675:Leucine-rich repeat domain superfamily IPR036047:F-box-like domain superfamily
		TraesCS6A02G369100	TraesCS6A02G369100.1		IPR032675:Leucine-rich repeat domain superfamily IPR036047:F-box-like domain superfamily
		TraesCS6A02G369600	TraesCS6A02G369600.1		IPR001810:F-box domain IPR032675:Leucine-rich repeat domain superfamily IPR036047:F-box-like domain superfamily
		TraesCS6A02G369700	TraesCS6A02G369700.1		IPR001128:Cytochrome P450 IPR002401:Cytochrome P450, E-class, group I IPR017972:Cytochrome P450, conserved site IPR036396:Cytochrome P450 superfamily
		TraesCS6A02G370000	TraesCS6A02G370000.1		IPR032675:Leucine-rich repeat domain superfamily IPR036047:F-box-like domain superfamily
		TraesCS6A02G370200	TraesCS6A02G370200.1		IPR001810:F-box domain IPR032675:Leucine-rich repeat domain superfamily IPR036047:F-box-like domain superfamily
		TraesCS6A02G370400	TraesCS6A02G370400.1		IPR032675:Leucine-rich repeat domain superfamily
		TraesCS6A02G370700	TraesCS6A02G370700.2		IPR001810:F-box domain IPR032675:Leucine-rich repeat domain superfamily IPR036047:F-box-like domain superfamily
		TraesCS6A02G371600	TraesCS6A02G371600.1		IPR001810:F-box domain IPR036047:F-box-like domain superfamily
		TraesCS6A02G371900	TraesCS6A02G371900.1		IPR032675:Leucine-rich repeat domain superfamily
6A		TraesCS6A02G372800	TraesCS6A02G372800.1		IPR032675:Leucine-rich repeat domain superfamily
		TraesCS6A02G373100	TraesCS6A02G373100.1		IPR001810:F-box domain IPR032675:Leucine-rich repeat domain superfamily IPR036047:F-box-like domain superfamily
		TraesCS6A02G373600	TraesCS6A02G373600.1		IPR013187:F-box associated domain, type 3 IPR017451:F-box associated interaction domain IPR036047:F-box-like domain superfamily
		TraesCS6A02G374100	TraesCS6A02G374100.1	Protein kinase superfamily protein [Source:Projected from Arabidopsis thaliana (AT2G39190) UniProtKB/TrEMBL;Acc:O80962]	IPR000719:Protein kinase domain IPR002575:Aminoglycoside phosphotransferase IPR004147:UbiB domain IPR011009:Protein kinase-like domain superfamily
		TraesCS6A02G374700	TraesCS6A02G374700.1	IRE1 [Source:UniProtKB/TrEMBL;Acc:A0A2C9PG52] IRE1	IPR000719:Protein kinase domain IPR008271:Serine/threonine-protein kinase, active site IPR010513:KEN domain IPR011009:Protein kinase-like domain superfamily IPR011047:Quinoprotein alcohol dehydrogenase-like superfamily IPR015943:WD40/YVTN repeat-like-containing domain superfamily IPR018997:PUB domain IPR038357:KEN domain superfamily
	AX.95097189	TraesCS6B02G067400	TraesCS6B02G067400.1		IPR001810:F-box domain IPR036047:F-box-like domain superfamily
		TraesCS6B02G067500	TraesCS6B02G067500.1		IPR032675:Leucine-rich repeat domain superfamily
		TraesCS6B02G067700	TraesCS6B02G067700.1		IPR001810:F-box domain IPR032675:Leucine-rich repeat domain superfamily IPR036047:F-box-like domain superfamily

	TraesCS6B02G067800	TraesCS6B02G067800.1	IPR000719:Protein kinase domain IPR008271:Serine/threonine-protein kinase, active site IPR011009:Protein kinase-like domain superfamily IPR017441:Protein kinase, ATP binding site IPR036537:Adaptor protein Cbl, N-terminal domain superfamily
	TraesCS6B02G069300	TraesCS6B02G069300.2	IPR000719:Protein kinase domain IPR001245:Serine-threonine/tyrosine-protein kinase, catalytic domain IPR011009:Protein kinase-like domain superfamily
	TraesCS6B02G069300	TraesCS6B02G069300.1	IPR000719:Protein kinase domain IPR001245:Serine-threonine/tyrosine-protein kinase, catalytic domain IPR011009:Protein kinase-like domain superfamily
	TraesCS6B02G069300	TraesCS6B02G069300.3	IPR000719:Protein kinase domain IPR001245:Serine-threonine/tyrosine-protein kinase, catalytic domain IPR011009:Protein kinase-like domain superfamily
	TraesCS6B02G069300	TraesCS6B02G069300.4	IPR000719:Protein kinase domain IPR001245:Serine-threonine/tyrosine-protein kinase, catalytic domain IPR011009:Protein kinase-like domain superfamily
	TraesCS6B02G069400	TraesCS6B02G069400.2	IPR002182:NB-ARC IPR027417:P-loop containing nucleoside triphosphate hydrolase IPR027806:Harbinger transposase-derived nuclease domain IPR032675:Leucine-rich repeat domain superfamily IPR038005:Virus X resistance protein-like, coiled-coil domain IPR041118:Rx, N-terminal
	TraesCS6B02G069400	TraesCS6B02G069400.3	IPR002182:NB-ARC IPR027417:P-loop containing nucleoside triphosphate hydrolase IPR027806:Harbinger transposase-derived nuclease domain IPR032675:Leucine-rich repeat domain superfamily
	TraesCS6B02G069400	TraesCS6B02G069400.1	IPR002182:NB-ARC IPR027417:P-loop containing nucleoside triphosphate hydrolase IPR032675:Leucine-rich repeat domain superfamily IPR038005:Virus X resistance protein-like, coiled-coil domain IPR041118:Rx, N-terminal
	TraesCS6B02G069500	TraesCS6B02G069500.1	IPR002182:NB-ARC IPR003591:Leucine-rich repeat, typical subtype IPR027417:P-loop containing nucleoside triphosphate hydrolase IPR027806:Harbinger transposase-derived nuclease domain IPR032675:Leucine-rich repeat domain superfamily IPR038005:Virus X resistance protein-like, coiled-coil domain IPR041118:Rx, N-terminal
	TraesCS6B02G069700	TraesCS6B02G069700.1	IPR002182:NB-ARC IPR027417:P-loop containing nucleoside triphosphate hydrolase IPR038005:Virus X resistance protein-like, coiled-coil domain IPR041118:Rx, N-terminal
	TraesCS6B02G069700	TraesCS6B02G069700.2	IPR002182:NB-ARC IPR027417:P-loop containing nucleoside triphosphate hydrolase IPR038005:Virus X resistance protein-like, coiled-coil domain IPR041118:Rx, N-terminal
	TraesCS6B02G069800	TraesCS6B02G069800.1	IPR000719:Protein kinase domain IPR001245:Serine-threonine/tyrosine-protein kinase, catalytic domain IPR008271:Serine/threonine-protein kinase, active site IPR011009:Protein kinase-like domain superfamily IPR017441:Protein kinase, ATP binding site
	TraesCS6B02G069900	TraesCS6B02G069900.2	IPR002182:NB-ARC IPR003591:Leucine-rich repeat, typical subtype IPR027417:P-loop containing nucleoside triphosphate hydrolase IPR027806:Harbinger transposase-derived nuclease domain IPR032675:Leucine-rich repeat domain superfamily IPR038005:Virus X resistance protein-like, coiled-coil domain IPR041118:Rx, N-terminal
	TraesCS6B02G069900	TraesCS6B02G069900.1	IPR002182:NB-ARC IPR003591:Leucine-rich repeat, typical subtype IPR027417:P-loop containing nucleoside triphosphate hydrolase IPR027806:Harbinger transposase-derived nuclease domain IPR032675:Leucine-rich repeat domain superfamily IPR038005:Virus X resistance protein-like, coiled-coil domain IPR041118:Rx, N-terminal
6B	TraesCS6B02G070200	TraesCS6B02G070200.1	IPR002182:NB-ARC IPR003591:Leucine-rich repeat, typical subtype IPR027417:P-loop containing nucleoside triphosphate hydrolase IPR032675:Leucine-rich repeat domain superfamily IPR038005:Virus X resistance protein-like, coiled-coil domain IPR041118:Rx, N-terminal

TraesCS6B02G070300	TraesCS6B02G070300.1		IPR002182:NB-ARC IPR027417:P-loop containing nucleoside triphosphate hydrolase IPR032675:Leucine-rich repeat domain superfamily
TraesCS6B02G070400	TraesCS6B02G070400.1		IPR002182:NB-ARC IPR027417:P-loop containing nucleoside triphosphate hydrolase IPR027806:Harbinger transposase-derived nuclease domain IPR032675:Leucine-rich repeat domain superfamily IPR038005:Virus X resistance protein-like, coiled-coil domain IPR041118:Rx, N-terminal
TraesCS6B02G070500	TraesCS6B02G070500.2		IPR002182:NB-ARC IPR003591:Leucine-rich repeat, typical subtype IPR027417:P-loop containing nucleoside triphosphate hydrolase IPR027806:Harbinger transposase-derived nuclease domain IPR032675:Leucine-rich repeat domain superfamily IPR038005:Virus X resistance protein-like, coiled-coil domain IPR041118:Rx, N-terminal
TraesCS6B02G070500	TraesCS6B02G070500.1		IPR002182:NB-ARC IPR003591:Leucine-rich repeat, typical subtype IPR027417:P-loop containing nucleoside triphosphate hydrolase IPR027806:Harbinger transposase-derived nuclease domain IPR032675:Leucine-rich repeat domain superfamily IPR038005:Virus X resistance protein-like, coiled-coil domain IPR041118:Rx, N-terminal
TraesCS6B02G070700	TraesCS6B02G070700.1		IPR002182:NB-ARC IPR003591:Leucine-rich repeat, typical subtype IPR027417:P-loop containing nucleoside triphosphate hydrolase IPR027806:Harbinger transposase-derived nuclease domain IPR032675:Leucine-rich repeat domain superfamily
TraesCS6B02G070800	TraesCS6B02G070800.1		IPR038005:Virus X resistance protein-like, coiled-coil domain IPR041118:Rx, N-terminal
TraesCS6B02G070900	TraesCS6B02G070900.3		IPR002182:NB-ARC IPR003591:Leucine-rich repeat, typical subtype IPR027417:P-loop containing nucleoside triphosphate hydrolase IPR032675:Leucine-rich repeat domain superfamily
TraesCS6B02G070900	TraesCS6B02G070900.1		IPR002182:NB-ARC IPR003591:Leucine-rich repeat, typical subtype IPR027417:P-loop containing nucleoside triphosphate hydrolase IPR027806:Harbinger transposase-derived nuclease domain IPR032675:Leucine-rich repeat domain superfamily IPR038005:Virus X resistance protein-like, coiled-coil domain IPR041118:Rx, N-terminal
TraesCS6B02G070900	TraesCS6B02G070900.2		IPR002182:NB-ARC IPR003591:Leucine-rich repeat, typical subtype IPR027417:P-loop containing nucleoside triphosphate hydrolase IPR027806:Harbinger transposase-derived nuclease domain IPR032675:Leucine-rich repeat domain superfamily IPR038005:Virus X resistance protein-like, coiled-coil domain IPR041118:Rx, N-terminal
TraesCS6B02G071000	TraesCS6B02G071000.1		IPR000719:Protein kinase domain IPR001611:Leucine-rich repeat IPR003591:Leucine-rich repeat, typical subtype IPR008271:Serine/threonine-protein kinase, active site IPR011009:Protein kinase-like domain superfamily IPR013210:Leucine-rich repeat-containing N-terminal, plant-type IPR017441:Protein kinase, ATP binding site IPR032675:Leucine-rich repeat domain superfamily
TraesCS6B02G071200	TraesCS6B02G071200.1	Peroxidase [Source:UniProtKB/TrEMBL;Acc:A0A1D6B2B4]	IPR000823:Plant peroxidase IPR002016:Haem peroxidase IPR010255:Haem peroxidase superfamily IPR019793:Peroxidases heam-ligand binding site IPR019794:Peroxidase, active site IPR033905:Secretory peroxidase
TraesCS6B02G072700	TraesCS6B02G072700.1		IPR001810:F-box domain IPR036047:F-box-like domain superfamily
TraesCS6B02G073000	TraesCS6B02G073000.1		IPR000719:Protein kinase domain IPR000742:EGF-like domain IPR001881:EGF-like calcium-binding domain IPR008271:Serine/threonine-protein kinase, active site IPR009030:Growth factor receptor cysteine-rich domain superfamily IPR011009:Protein kinase-like domain superfamily IPR017441:Protein kinase, ATP binding site IPR025287:Wall-associated receptor kinase, galacturonan-binding domain

6B	AX.94407285	TraesCS6B02G199500	TraesCS6B02G199500.1		IPR000719:Protein kinase domain IPR001611:Leucine-rich repeat IPR003591:Leucine-rich repeat, typical subtype IPR011009:Protein kinase-like domain superfamily IPR013210:Leucine-rich repeat-containing N-terminal, plant-type IPR032675:Leucine-rich repeat domain superfamily
		TraesCS6B02G200100	TraesCS6B02G200100.1		IPR000719:Protein kinase domain IPR001245:Serine-threonine/tyrosine-protein kinase, catalytic domain IPR008271:Serine/threonine-protein kinase, active site IPR011009:Protein kinase-like domain superfamily
		TraesCS6B02G200700	TraesCS6B02G200700.1		IPR001810:F-box domain IPR006566:FBD domain IPR036047:F-box-like domain superfamily
7A	AX.94978939	TraesCS7A02G455300	TraesCS7A02G455300.1	Cytochrome P450 like protein [Source:Projected from Arabidopsis thaliana (AT1G11600) UniProtKB/TrEMBL;Acc:Q9SAB7]	IPR001128:Cytochrome P450 IPR002401:Cytochrome P450, E-class, group I IPR017972:Cytochrome P450, conserved site IPR036396:Cytochrome P450 superfamily
		TraesCS7A02G455700	TraesCS7A02G455700.1		IPR000719:Protein kinase domain IPR008271:Serine/threonine-protein kinase, active site IPR011009:Protein kinase-like domain superfamily
		TraesCS7A02G455800	TraesCS7A02G455800.1	Serine/threonine-protein kinase [Source:UniProtKB/TrEMBL;Acc:A0A1D6BQI9]	IPR000719:Protein kinase domain IPR000858:S-locus glycoprotein domain IPR001480:Bulb-type lectin domain IPR003609:PAN/Apple domain IPR008271:Serine/threonine-protein kinase, active site IPR011009:Protein kinase-like domain superfamily IPR017441:Protein kinase, ATP binding site IPR024171:S-receptor-like serine/threonine-protein kinase IPR036426:Bulb-type lectin domain superfamily
		TraesCS7A02G456100	TraesCS7A02G456100.1		IPR000719:Protein kinase domain IPR000858:S-locus glycoprotein domain IPR001245:Serine-threonine/tyrosine-protein kinase, catalytic domain IPR001480:Bulb-type lectin domain IPR003609:PAN/Apple domain IPR011009:Protein kinase-like domain superfamily IPR017441:Protein kinase, ATP binding site IPR036426:Bulb-type lectin domain superfamily
		TraesCS7A02G456200	TraesCS7A02G456200.1		IPR000719:Protein kinase domain IPR008271:Serine/threonine-protein kinase, active site IPR011009:Protein kinase-like domain superfamily
		TraesCS7A02G456300	TraesCS7A02G456300.1		IPR000719:Protein kinase domain IPR000858:S-locus glycoprotein domain IPR001480:Bulb-type lectin domain IPR003609:PAN/Apple domain IPR008271:Serine/threonine-protein kinase, active site IPR011009:Protein kinase-like domain superfamily IPR017441:Protein kinase, ATP binding site IPR024171:S-receptor-like serine/threonine-protein kinase IPR036426:Bulb-type lectin domain superfamily
		TraesCS7A02G456800	TraesCS7A02G456800.1		IPR000719:Protein kinase domain IPR000858:S-locus glycoprotein domain IPR001245:Serine-threonine/tyrosine-protein kinase, catalytic domain IPR001480:Bulb-type lectin domain IPR003609:PAN/Apple domain IPR011009:Protein kinase-like domain superfamily IPR036426:Bulb-type lectin domain superfamily
		TraesCS7A02G456900	TraesCS7A02G456900.1		IPR011009:Protein kinase-like domain superfamily
		TraesCS7A02G457500	TraesCS7A02G457500.1		IPR032675:Leucine-rich repeat domain superfamily
	AX.94535772	TraesCS7B02G045500	TraesCS7B02G045500.1		IPR001611:Leucine-rich repeat IPR003591:Leucine-rich repeat, typical subtype IPR013210:Leucine-rich repeat-containing N-terminal, plant-type IPR032675:Leucine-rich repeat domain superfamily
		TraesCS7B02G045600	TraesCS7B02G045600.1		IPR000719:Protein kinase domain IPR001611:Leucine-rich repeat IPR003591:Leucine-rich repeat, typical subtype IPR008271:Serine/threonine-protein kinase, active site IPR011009:Protein kinase-like domain superfamily IPR013210:Leucine-rich repeat-containing N-terminal, plant-type IPR032675:Leucine-rich repeat domain superfamily

7B		TraesCS7B02G047300	TraesCS7B02G047300.4		IPR000719:Protein kinase domain IPR001245:Serine-threonine/tyrosine-protein kinase, catalytic domain IPR008271:Serine/threonine-protein kinase, active site IPR011009:Protein kinase-like domain superfamily IPR017441:Protein kinase, ATP binding site
		TraesCS7B02G047300	TraesCS7B02G047300.1		IPR000270:PB1 domain IPR000719:Protein kinase domain IPR001245:Serine-threonine/tyrosine-protein kinase, catalytic domain IPR008271:Serine/threonine-protein kinase, active site IPR011009:Protein kinase-like domain superfamily IPR017441:Protein kinase, ATP binding site
		TraesCS7B02G047300	TraesCS7B02G047300.3		IPR000719:Protein kinase domain IPR001245:Serine-threonine/tyrosine-protein kinase, catalytic domain IPR008271:Serine/threonine-protein kinase, active site IPR011009:Protein kinase-like domain superfamily IPR017441:Protein kinase, ATP binding site
		TraesCS7B02G047300	TraesCS7B02G047300.2		IPR000270:PB1 domain IPR000719:Protein kinase domain IPR001245:Serine-threonine/tyrosine-protein kinase, catalytic domain IPR008271:Serine/threonine-protein kinase, active site IPR011009:Protein kinase-like domain superfamily IPR017441:Protein kinase, ATP binding site
		TraesCS7B02G048900	TraesCS7B02G048900.1		IPR000719:Protein kinase domain IPR001245:Serine-threonine/tyrosine-protein kinase, catalytic domain IPR008271:Serine/threonine-protein kinase, active site IPR011009:Protein kinase-like domain superfamily IPR017441:Protein kinase, ATP binding site
	#N/A	TraesCS7B02G434800	TraesCS7B02G434800.1		IPR001810:F-box domain IPR006566:FBD domain IPR032675:Leucine-rich repeat domain superfamily IPR036047:F-box-like domain superfamily
		TraesCS7B02G434900	TraesCS7B02G434900.1		IPR006566:FBD domain IPR032675:Leucine-rich repeat domain superfamily IPR036047:F-box-like domain superfamily
		TraesCS7B02G435000	TraesCS7B02G435000.1		IPR032675:Leucine-rich repeat domain superfamily IPR036047:F-box-like domain superfamily
		TraesCS7B02G435100	TraesCS7B02G435100.1		IPR006566:FBD domain IPR032675:Leucine-rich repeat domain superfamily IPR036047:F-box-like domain superfamily
		TraesCS7B02G435200	TraesCS7B02G435200.1		IPR006566:FBD domain IPR032675:Leucine-rich repeat domain superfamily IPR036047:F-box-like domain superfamily
		TraesCS7B02G436300	TraesCS7B02G436300.1		IPR032675:Leucine-rich repeat domain superfamily
		TraesCS7B02G437300	TraesCS7B02G437300.1		IPR002182:NB-ARC IPR008480:Protein of unknown function DUF761, plant IPR027417:P-loop containing nucleoside triphosphate hydrolase IPR032675:Leucine-rich repeat domain superfamily IPR038005:Virus X resistance protein-like, coiled-coil domain IPR041118:Rx, N-terminal
		TraesCS7B02G437300	TraesCS7B02G437300.2		IPR002182:NB-ARC IPR027417:P-loop containing nucleoside triphosphate hydrolase IPR032675:Leucine-rich repeat domain superfamily IPR038005:Virus X resistance protein-like, coiled-coil domain IPR041118:Rx, N-terminal
		TraesCS7B02G437300	TraesCS7B02G437300.3		IPR002182:NB-ARC IPR027417:P-loop containing nucleoside triphosphate hydrolase IPR032675:Leucine-rich repeat domain superfamily IPR038005:Virus X resistance protein-like, coiled-coil domain IPR041118:Rx, N-terminal
		TraesCS7B02G437400	TraesCS7B02G437400.1		IPR002182:NB-ARC IPR027417:P-loop containing nucleoside triphosphate hydrolase IPR032675:Leucine-rich repeat domain superfamily IPR041118:Rx, N-terminal
		TraesCS7B02G437800	TraesCS7B02G437800.1	Cytochrome P450 [Source:UniProtKB/TrEMBL;Acc:Q9AVM3] Cytochrome P450	IPR001128:Cytochrome P450 IPR002401:Cytochrome P450, E-class, group I IPR017972:Cytochrome P450, conserved site IPR036396:Cytochrome P450 superfamily
		TraesCS7B02G438400	TraesCS7B02G438400.1		IPR001128:Cytochrome P450 IPR002401:Cytochrome P450, E-class, group I IPR017972:Cytochrome P450, conserved site IPR036396:Cytochrome P450 superfamily
		TraesCS7B02G438500	TraesCS7B02G438500.1		IPR001128:Cytochrome P450 IPR002401:Cytochrome P450, E-class, group I IPR017972:Cytochrome P450, conserved site IPR036396:Cytochrome P450 superfamily

7B		TraesCS7B02G438600	TraesCS7B02G438600.1	IPR001128:Cytochrome P450 IPR002401:Cytochrome P450, E-class, group I IPR017972:Cytochrome P450, conserved site IPR036396:Cytochrome P450 superfamily
		TraesCS7B02G438700	TraesCS7B02G438700.1	IPR001128:Cytochrome P450 IPR002401:Cytochrome P450, E-class, group I IPR017972:Cytochrome P450, conserved site IPR036396:Cytochrome P450 superfamily
		TraesCS7B02G438900	TraesCS7B02G438900.1	IPR001128:Cytochrome P450 IPR002401:Cytochrome P450, E-class, group I IPR017972:Cytochrome P450, conserved site IPR036396:Cytochrome P450 superfamily
		TraesCS7B02G439000	TraesCS7B02G439000.1	IPR001128:Cytochrome P450 IPR002401:Cytochrome P450, E-class, group I IPR017972:Cytochrome P450, conserved site IPR036396:Cytochrome P450 superfamily
		TraesCS7B02G439300	TraesCS7B02G439300.1	IPR001128:Cytochrome P450 IPR002401:Cytochrome P450, E-class, group I IPR017972:Cytochrome P450, conserved site IPR036396:Cytochrome P450 superfamily
		TraesCS7B02G439400	TraesCS7B02G439400.1	IPR001128:Cytochrome P450 IPR002401:Cytochrome P450, E-class, group I IPR017972:Cytochrome P450, conserved site IPR036396:Cytochrome P450 superfamily
		TraesCS7B02G439700	TraesCS7B02G439700.1	IPR001128:Cytochrome P450 IPR002401:Cytochrome P450, E-class, group I IPR017972:Cytochrome P450, conserved site IPR036396:Cytochrome P450 superfamily
		TraesCS7B02G439800	TraesCS7B02G439800.1	IPR001128:Cytochrome P450 IPR002401:Cytochrome P450, E-class, group I IPR017972:Cytochrome P450, conserved site IPR036396:Cytochrome P450 superfamily
		TraesCS7B02G440000	TraesCS7B02G440000.2	IPR000719:Protein kinase domain IPR001245:Serine-threonine/tyrosine-protein kinase, catalytic domain IPR011009:Protein kinase-like domain superfamily
		TraesCS7B02G440000	TraesCS7B02G440000.1	IPR000719:Protein kinase domain IPR001245:Serine-threonine/tyrosine-protein kinase, catalytic domain IPR011009:Protein kinase-like domain superfamily
		TraesCS7B02G440600	TraesCS7B02G440600.1	IPR001128:Cytochrome P450 IPR002401:Cytochrome P450, E-class, group I IPR017972:Cytochrome P450, conserved site IPR036396:Cytochrome P450 superfamily
		TraesCS7B02G440700	TraesCS7B02G440700.1	IPR001128:Cytochrome P450 IPR036396:Cytochrome P450 superfamily
		TraesCS7B02G440800	TraesCS7B02G440800.2	IPR032675:Leucine-rich repeat domain superfamily
		TraesCS7B02G440800	TraesCS7B02G440800.1	IPR032675:Leucine-rich repeat domain superfamily
		TraesCS7B02G440900	TraesCS7B02G440900.1	IPR001128:Cytochrome P450 IPR002401:Cytochrome P450, E-class, group I IPR017972:Cytochrome P450, conserved site IPR036396:Cytochrome P450 superfamily
		TraesCS7B02G441100	TraesCS7B02G441100.1	IPR001128:Cytochrome P450 IPR002401:Cytochrome P450, E-class, group I IPR017972:Cytochrome P450, conserved site IPR036396:Cytochrome P450 superfamily
		TraesCS7B02G441200	TraesCS7B02G441200.1	IPR000719:Protein kinase domain IPR001611:Leucine-rich repeat IPR011009:Protein kinase-like domain superfamily IPR013210:Leucine-rich repeat-containing N-terminal, plant-type IPR032675:Leucine-rich repeat domain superfamily
	AX.94860625	TraesCS7D02G539900	TraesCS7D02G539900.1	IPR001810:F-box domain IPR005174:Domain unknown function DUF295 IPR036047:F-box-like domain superfamily
		TraesCS7D02G540000	TraesCS7D02G540000.1	IPR038005:Virus X resistance protein-like, coiled-coil domain IPR041118:Rx, N-terminal
		TraesCS7D02G540100	TraesCS7D02G540100.1	IPR001810:F-box domain IPR005174:Domain unknown function DUF295 IPR036047:F-box-like domain superfamily
		TraesCS7D02G540500	TraesCS7D02G540500.1	IPR002182:NB-ARC IPR027417:P-loop containing nucleoside triphosphate hydrolase IPR032675:Leucine-rich repeat domain superfamily IPR038005:Virus X resistance protein-like, coiled-coil domain IPR041118:Rx, N-terminal
		TraesCS7D02G540600	TraesCS7D02G540600.1	IPR002182:NB-ARC IPR027417:P-loop containing nucleoside triphosphate hydrolase IPR038005:Virus X resistance protein-like, coiled-coil domain IPR041118:Rx, N-terminal

	TraesCS7D02G541200	TraesCS7D02G541200.1	Protein DETOXIFICATION [Source:UniProtKB/TrEMBL;Acc:W5HZB8]	IPR002528:Multi antimicrobial extrusion protein
	TraesCS7D02G541800	TraesCS7D02G541800.1	Protein DETOXIFICATION [Source:UniProtKB/TrEMBL;Acc:A0A341Z7X7]	IPR002528:Multi antimicrobial extrusion protein
	TraesCS7D02G542100	TraesCS7D02G542100.1		IPR032675:Leucine-rich repeat domain superfamily
	TraesCS7D02G542200	TraesCS7D02G542200.1		IPR002182:NB-ARC IPR027417:P-loop containing nucleoside triphosphate hydrolase IPR032675:Leucine-rich repeat domain superfamily IPR036388:Winged helix-like DNA-binding domain superfamily
	TraesCS7D02G542300	TraesCS7D02G542300.1		IPR002182:NB-ARC IPR027417:P-loop containing nucleoside triphosphate hydrolase IPR038005:Virus X resistance protein-like, coiled-coil domain IPR041118:Rx, N-terminal
	TraesCS7D02G542500	TraesCS7D02G542500.1		IPR032675:Leucine-rich repeat domain superfamily
	TraesCS7D02G542600	TraesCS7D02G542600.1		IPR001611:Leucine-rich repeat IPR003591:Leucine-rich repeat, typical subtype IPR013210:Leucine-rich repeat-containing N-terminal, plant-type IPR032675:Leucine-rich repeat domain superfamily
	TraesCS7D02G542700	TraesCS7D02G542700.1		IPR000719:Protein kinase domain IPR001611:Leucine-rich repeat IPR003591:Leucine-rich repeat, typical subtype IPR008266:Tyrosine-protein kinase, active site IPR011009:Protein kinase-like domain superfamily IPR017441:Protein kinase, ATP binding site IPR032675:Leucine-rich repeat domain superfamily
	TraesCS7D02G543900	TraesCS7D02G543900.1		IPR002182:NB-ARC IPR027417:P-loop containing nucleoside triphosphate hydrolase IPR038005:Virus X resistance protein-like, coiled-coil domain IPR041118:Rx, N-terminal
	TraesCS7D02G544100	TraesCS7D02G544100.1		IPR002182:NB-ARC IPR027417:P-loop containing nucleoside triphosphate hydrolase IPR032675:Leucine-rich repeat domain superfamily IPR038005:Virus X resistance protein-like, coiled-coil domain IPR041118:Rx, N-terminal
7D	TraesCS7D02G544300	TraesCS7D02G544300.1		IPR002182:NB-ARC IPR027417:P-loop containing nucleoside triphosphate hydrolase IPR027806:Harbinger transposase-derived nuclease domain IPR032675:Leucine-rich repeat domain superfamily IPR038005:Virus X resistance protein-like, coiled-coil domain IPR041118:Rx, N-terminal
	TraesCS7D02G544500	TraesCS7D02G544500.1		IPR002182:NB-ARC IPR003591:Leucine-rich repeat, typical subtype IPR027417:P-loop containing nucleoside triphosphate hydrolase IPR027806:Harbinger transposase-derived nuclease domain IPR032675:Leucine-rich repeat domain superfamily IPR038005:Virus X resistance protein-like, coiled-coil domain IPR041118:Rx, N-terminal
	TraesCS7D02G544600	TraesCS7D02G544600.1		IPR002182:NB-ARC IPR027417:P-loop containing nucleoside triphosphate hydrolase IPR032675:Leucine-rich repeat domain superfamily IPR038005:Virus X resistance protein-like, coiled-coil domain IPR041118:Rx, N-terminal
	TraesCS7D02G546100	TraesCS7D02G546100.1		IPR002182:NB-ARC IPR003593:AAA+ ATPase domain IPR027417:P-loop containing nucleoside triphosphate hydrolase IPR032675:Leucine-rich repeat domain superfamily IPR041118:Rx, N-terminal
	TraesCS7D02G546400	TraesCS7D02G546400.1		IPR002182:NB-ARC IPR003593:AAA+ ATPase domain IPR027417:P-loop containing nucleoside triphosphate hydrolase IPR032675:Leucine-rich repeat domain superfamily IPR041118:Rx, N-terminal
	TraesCS7D02G546500	TraesCS7D02G546500.1		IPR002182:NB-ARC IPR003593:AAA+ ATPase domain IPR027417:P-loop containing nucleoside triphosphate hydrolase IPR041118:Rx, N-terminal

TraesCS7D02G546800	TraesCS7D02G546800.1		IPR036047:F-box-like domain superfamily
TraesCS7D02G547100	TraesCS7D02G547100.1		IPR002182:NB-ARC IPR027417:P-loop containing nucleoside triphosphate hydrolase IPR032675:Leucine-rich repeat domain superfamily IPR038005:Virus X resistance protein-like, coiled-coil domain IPR041118:Rx, N-terminal
TraesCS7D02G548100	TraesCS7D02G548100.1		IPR002182:NB-ARC IPR027417:P-loop containing nucleoside triphosphate hydrolase IPR032675:Leucine-rich repeat domain superfamily IPR038005:Virus X resistance protein-like, coiled-coil domain IPR041118:Rx, N-terminal
TraesCS7D02G549000	TraesCS7D02G549000.1		IPR001611:Leucine-rich repeat IPR003591:Leucine-rich repeat, typical subtype IPR013210:Leucine-rich repeat-containing N-terminal, plant-type IPR032675:Leucine-rich repeat domain superfamily
TraesCS7D02G549200	TraesCS7D02G549200.1		IPR002182:NB-ARC IPR027417:P-loop containing nucleoside triphosphate hydrolase IPR032675:Leucine-rich repeat domain superfamily
TraesCS7D02G549500	TraesCS7D02G549500.1		IPR001611:Leucine-rich repeat IPR003591:Leucine-rich repeat, typical subtype IPR013210:Leucine-rich repeat-containing N-terminal, plant-type IPR032675:Leucine-rich repeat domain superfamily
TraesCS7D02G549700	TraesCS7D02G549700.1		IPR002182:NB-ARC IPR027417:P-loop containing nucleoside triphosphate hydrolase IPR038005:Virus X resistance protein-like, coiled-coil domain IPR041118:Rx, N-terminal
TraesCS7D02G550000	TraesCS7D02G550000.1		IPR036047:F-box-like domain superfamily
TraesCS7D02G550100	TraesCS7D02G550100.1	F-box domain-containing protein	IPR001810:F-box domain IPR036047:F-box-like domain superfamily
TraesCS7D02G550300	TraesCS7D02G550300.1		IPR001810:F-box domain IPR036047:F-box-like domain superfamily
TraesCS7D02G550400	TraesCS7D02G550400.1		IPR001810:F-box domain IPR036047:F-box-like domain superfamily
TraesCS7D02G551100	TraesCS7D02G551100.1		IPR001810:F-box domain IPR036047:F-box-like domain superfamily
