# Supplementary material for: GWAS analysis reveals distinct pathogenicity profiles of Australian Parastagonospora nodorum isolates and identification of marker-trait-associations to septoria nodorum blotch
Source: Sci Rep. 2021 May 12;11:10085. doi: 10.1038/s41598-021-87829-0 (PMC8115087; doi:10.1038/s41598-021-87829-0)
Supplement: Supplementary file 4 — Supplementary Table S3. [file 41598_2021_87829_MOESM4_ESM.pdf]

Supplemental Table S3: List of SnTox3 sensitive wheat lines carrying *Snn3-D1* locus.

GWAS analysis reveals distinct pathogenicity profiles of Australian *Parastagonospora nodorum* isolates and identification of marker-trait-associations to septoria nodorum blotch

Huyen T.T Phan, Eiko Furuki, Lukas Hunziker, Kasia Rybak, Kar-Chun Tan

Supplemental Table S3: List of wheat lines carrying *Snn3-D1* locus.

Collection	Qcode	Collection	GID	Pedigree	synthetic wheat
**CAIGE**	115:ZWW11	CAIGE 2013	6280103	PBW343*2/KUKUNA//WBLL1*2/KUKUNA	
	121:ZWB11	CAIGE 2013	6174903	ALTAR 84/AE.SQUARROSA (221)//3*BORL95/3/URES/JUN//KAUZ/4/WBLL1/5/MILAN/S87230//BAV92	synthetic
	124:ZWB11	CAIGE 2013	6177148	TRCH/6/HPO/TAN//VEE/3/2*PGO/4/MILAN/5/SSERI1	
	126:ZWB11	CAIGE 2013	6177174	PFAU/SERI.1B//AMAD/3/WAXWING/6/HPO/TAN//VEE/3/2*PGO/4/MILAN/5/SSERI1	
	172:ZWB11	CAIGE 2013	6175216	WAXWING/4/BL 1496/MILAN/3/CROC_1/AE.SQUARROSA (205)//KAUZ/5/FRNCLN	synthetic
	182:ZWB11	CAIGE 2013	6175411	WAXWING*2/HEILO	
	24:ZIZ11	CAIGE 2013	5755188	SHA5//CARC/AUK/3/VEE#5//DOBUC'S'	
	34:ZWW11	CAIGE 2013	6279212	STYLET/4/SLVS/3/CROC_1/AE.SQUARROSA (224)//OPATA	synthetic
	45:ZIZ11	CAIGE 2013	400002141	GARIBE//TURACO/CHIL/3/KATILA-11	
	56:ZWB11	CAIGE 2013	6176308	ATTILA*2/PBW65*2//MURGA	
	66:ZWB11	CAIGE 2013	6176409	ATTILA*2/PBW65*2//W485/HD29	
	71:ZIZ11	CAIGE 2013	400002058	HAALA-37	
	80:ZWB11	CAIGE 2013	6176558	ATTILA*2/PBW65*2/4/BOW/NKT//CBRD/3/CBRD	
	95:ZWB11	CAIGE 2013	6176914	MUNAL #1/FRANCOLIN #1	
	96:ZWB11	CAIGE 2013	6177828	FRNCLN/ROLF07	
**Vavilov**	**Name**	**Year of collec**	**Origin**	**Status of accession**	
	WLA-016	-	-	-	
	WLA-304	1990	Russia	Breeding line	
